# Real‐World Effectiveness and Safety of Triple‐Mite SCIT With *Dermatophagoides* and *Blomia tropicalis*

**DOI:** 10.1002/clt2.70217

**Published:** 2026-10-10

**Authors:** José Carlos García‐Robaina, Cristina González‐Colino, Guacimara Lucia Hernández‐Santana, Elena Rodríguez‐Plata, Mónica González‐Afonso, Ariel Callero, Juan Antonio Martínez‐Tadeo, María José Menés, Miguel Casanovas, Yara Ruiz García, Judith Díaz‐García

**Affiliations:** ^1^ Allergy Department Hospital Universitario Nuestra Señora de Candelaria Tenerife Spain; ^2^ Inmunotek, S.L. Alcalá de Henares Spain

**Keywords:** allergen immunotherapy, *Blomia tropicalis*, house dust mite, real‐world evidence, subcutaneous immunotherapy

## Abstract

**Background:**

House dust mite (HDM)‐induced respiratory allergy is a major global health problem. Species distribution varies by region and climate; *Blomia tropicalis* is prevalent in tropical and subtropical areas and often coexists with *Dermatophagoides* spp. Although allergen immunotherapy (AIT) reduces symptoms and medication use, real‐world evidence for formulations including *B. tropicalis* remains limited.

**Objectives:**

To evaluate the real‐world effectiveness and safety of triple‐mite subcutaneous immunotherapy (SCIT) with glutaraldehyde‐polymerized extracts of *Dermatophagoides pteronyssinus*, *Dermatophagoides farinae*, and *B. tropicalis*.

**Methods:**

Clinical records of 208 patients (5–65 years) with mite‐induced allergic rhinoconjunctivitis, with or without asthma, sensitized to these mites were analyzed. All received ≥ 12 months of SCIT. Follow‐up extended up to 5 years. Effectiveness was assessed annually using mixed models for repeated measures based on combined symptom and medication scores for rhinoconjunctivitis (RCSMS) and asthma (ACSMS), and individual symptom and medication scores. Safety was evaluated by recording adverse reactions.

**Results:**

Significant and sustained improvements were observed. Adjusted LS‐mean RCSMS decreased from 3.97 at baseline to 2.26 at 1 year and 1.24 at 5 years (−43.1% and −68.8%; *p* < 0.001). ACSMS decreased from 2.74 to 1.36 and 0.46 (−50.5% and −83.3%). Symptom improvement was early and accompanied by progressive reductions in medication use. Safety was favourable, with one mild grade 1 systemic reaction (0.48% of patients; 0.015% per injection), resolving without treatment modification.

**Conclusions:**

Triple‐mite SCIT was associated with sustained, clinically meaningful reductions in symptoms and medication use, and showed a favourable safety profile, supporting a favourable benefit–risk balance in regions where these mites coexist.

## 1 Introduction

House dust mites (HDM) are a major trigger of allergic rhinitis and asthma worldwide, affecting up to 50% of patients with asthma [1]. Allergic rhinitis also represents a substantial socio‐economic burden in Europe [2].

The distribution of mite species varies across geographic regions and climates, influencing sensitization patterns and clinical manifestations. While *Dermatophagoides pteronyssinus* and *Dermatophagoides farinae* are the predominant sources of HDM allergens in temperate climates, *Blomia tropicalis* has clinical relevance in tropical and subtropical regions, where it frequently coexists with *Dermatophagoides* spp [3, 4].

The clinical relevance of *B. tropicalis* has been described in Latin American and Asian countries, where it represents a major cause of sensitization and allergic disease [3, 4]. In Spain, it has traditionally been reported in the Canary Islands [5], where early faunistic studies identified *B. tropicalis* in domestic dust samples together with a nearly universal presence of *D. pteronyssinus* [6]. This region also shows the highest proportion of mite sensitization nationwide [7], and recent reports suggest increasing sensitization to HDM, particularly to *B. tropicalis*, in other areas such as Santander [8].

This geographic expansion is likely driven by climatic conditions and climate change [3, 4], underlining the importance of considering *B. tropicalis* in diagnostic and therapeutic strategies beyond traditionally tropical regions.

Importantly, polysensitization to multiple mite species has been associated with increased disease severity and symptom persistence, and with a higher likelihood of concomitant asthma [3, 4, 9]. In such settings, incomplete allergenic coverage may contribute to suboptimal responses to single‐mite immunotherapy, supporting combined‐mite vaccines.

Allergen immunotherapy (AIT) is the only disease‐modifying treatment available for allergic rhinitis and asthma [10]. HDM immunotherapy has demonstrated efficacy and effectiveness in improving symptoms, reducing medication use, and inducing long‐term tolerance [9, 11, 12, 13, 14]. However, clinical data and real‐world evidence regarding *Dermatophagoides* and *B. tropicalis* immunotherapy remain scarce.

We aimed to evaluate the real‐world effectiveness and safety of subcutaneous immunotherapy with a mixture of individually glutaraldehyde‐polymerized extracts of *D. pteronyssinus*, *D. farinae* and *B. tropicalis* in patients with mite‐induced allergic respiratory disease in Tenerife, Spain.

## 2 Methods

### 2.1 Study Design

This was a two‐center, observational, retrospective real‐world study based on the review of patients' medical records. The study evaluated the effectiveness and safety of a mixture of individually polymerized extracts of *Dermatophagoides* spp. and *B. tropicalis* administered in routine clinical practice. Data were collected under routine clinical conditions. The study was registered at ClinicalTrials.gov (NCT07427576).

### 2.2 Patient Population

Patients aged 5–65 years with allergic respiratory diseases (rhinoconjunctivitis with/without asthma) who had received ≥ 12 months of allergen immunotherapy with these Inmunotek's *Dermatophagoides*/*B. tropicalis* preparation were included. Patients were attended at either Hospital Universitario Ntra. Sra. de Candelaria or Clínica de Alergia Dr. García Robaina (Santa Cruz de Tenerife, Spain) from 2015 to 2025.

Patients with prior AIT with aeroallergens before the course evaluated in this study were excluded.

Allergy was clinically relevant in each patient and was documented by positive skin prick test (SPT ≥ 3 mm) and/or by serum specific IgE determination by ImmunoCAP (Thermo Fisher Scientific, Uppsala, Sweden) and/or ALEX^2^ (Macro Array Diagnostics GmbH, Vienna, Austria). Treatment with the triple‐mite preparation was indicated by the prescribing physician according to each patient's individual, clinically relevant mite sensitization pattern and routine clinical practice.

### 2.3 Immunotherapy Preparation and Treatment Schedule

Patients received subcutaneous allergen immunotherapy (SCIT) with *Dermatophagoides*/*B. tropicalis*, containing a mixture of individually glutaraldehyde‐polymerized extracts of *Dermatophagoides* spp. (*D. pteronyssinus* and *D. farinae*) and *B. tropicalis*, adsorbed onto aluminum hydroxide, with phenol and sodium chloride as excipients, developed and manufactured by Inmunotek S.L (Alcalá de Henares, Spain).

In Spain, the study product is marketed under the trade name Clustoid/Alxoid, whereas outside Spain it is marketed as Clustek/Alxoid.

Dosing schedules and dose adjustments followed usual clinical practice. The rush schedule consisted of 0.2 mL and, 30 minutes later, 0.3 mL, followed by 0.5 mL maintenance doses every 30 days; the minimum treatment duration for inclusion was 12 months.

### 2.4 Outcomes

Effectiveness endpoint was evaluated by the Combined Symptom and Medication Score (CSMS) for both rhinoconjunctivitis and asthma as primary outcome. The evolution of Symptom (SS) and Medication Scores (MS) were also evaluated.

Rhinoconjunctivitis symptoms considered for the study were itchy nose, sneezing, runny nose, blocked nose, itchy/red eyes, and watery eyes; whereas asthma symptoms were cough, dyspnoea, chest tightness and wheezing.

Rhinoconjunctivitis CSMS (RCSMS), SS (RSS), and MS (RMS) were assessed according to the European Association of Allergy and Immunology (EAACI) position paper [15]. RSS was assigned in a scale from 0 to 3 according to the intensity of each of the six symptoms mentioned above: no symptoms, mild, moderate, or severe symptoms, respectively. RSS was calculated using the sum of the individual scores for the six symptoms, divided by six. In the case of RMS, a criterion from 0 to 3 was used according to the need for medication: no medication, oral or topical antihistamines (eyes and/or nose), intranasal corticosteroids with or without antihistamines, and oral corticosteroids with or without antihistamines, respectively. RCSMS was calculated as the sum of RSS and RMS.

Regarding asthma SS (ASS), the same 0‐3 scoring criteria as described for RSS was followed according to the intensity of each of the four symptoms mentioned above; and SS was calculated using the sum of the individual scores for the four symptoms, divided by four. For Asthma MS (AMS), the scoring was based on the therapeutic steps for maintenance treatment of asthma published in the Spanish Guidelines for Asthma Management (GEMA v. 5.5) with minor modifications [16], as follows: 0: No medication; 0.5: Short‐Acting β‐2 Agonists (SABA) on demand; 1: Low‐dose inhaled corticosteroids (IC)/leukotriene receptor antagonists (LTRA); 1.5: Low‐dose IC + Long‐Acting β‐2 Agonists (LABA)/mid‐dose IC with or without LTRA with or without SABA on demand; 2: Medium‐dose IC + LABA with or without LTRA with or without SABA on demand; 2.5: High‐dose IC + LABA with or without LTRA with or without SABA on demand; 3: High‐dose IC + LABA + Systemic corticosteroids (SC) with or without LTRA with or without SABA on demand. Asthma CSMS (ACSMS) was calculated as the sum of ASS and AMS [17].

Effectiveness was assessed at baseline (T0, before the start of immunotherapy) and annually thereafter for at least 1 year (T1), up to a maximum of 5 years (T5) of follow‐up.

Safety endpoint was assessed through the recording and description of adverse reactions (ARs) during the observation period. ARs were categorized as local AR when occurring at the injection site (immediate ≤ 30 min, or late > 30 min), and were considered clinically relevant if they exceed 5 cm in diameter and occur within 30 minutes of administration, or exceed 10 cm in diameter when they occur later. ARs were categorized as systemic AR when occurring away from the injection site, typically within minutes and rarely after 30 min; and graded according to World Allergy Organization (WAO) guidance (five‐grade scale) [18]. All adverse reactions reported by the treating physicians were recorded. However, because safety data were collected retrospectively from routine clinical records, the systematic capture of all minor, non‐clinically relevant local reactions could not be guaranteed. Therefore, incidence calculations focused on clinically relevant adverse reactions.

### 2.5 Data Analysis

Data were collected from the clinical records at both centers using a common study data collection booklet, and the scoring of symptoms and medication were assigned by the prescribing physicians, based on their clinical interpretation of the patient's clinical records, using the same predefined scoring criteria described above. Although the source records were generated under routine clinical practice and were not prospectively standardized across centers, the use of a common data collection booklet and uniform scoring criteria supported a consistent approach to score derivation. No clinical intervention was derived from this study.

Baseline characteristics were summarized descriptively. Qualitative variables (gender and asthma diagnosis) were presented as frequencies with exact 95% confidence intervals (CIs). Age was summarized as mean (and standard deviation, SD) and median (and interquartile range, IQR) for the overall cohort and stratified by gender and asthma status. Contingency tables were performed using the χ2 test to determine whether there was a relationship between age range and gender with the diagnosis of asthma.

To assess effectiveness, the primary analysis was conducted using Mixed Models for Repeated Measures (MMRM) to evaluate longitudinal changes while accounting for within‐subject correlations across repeated measurements [19, 20].

For RCSMS, RSS and RMS, fixed effects included time (categorical), gender, age (continuous), and asthma status (yes/no). For ACSMS, ASS and AMS, analyses were restricted to patients with asthma and included time, gender and age as fixed effects.

An autoregressive covariance structure of order 1 (AR [1]) was specified to model within‐subject correlations. Parameters were estimated using restricted maximum likelihood (REML). Least‐squares means (LS‐means) with standard errors and 95% CIs were estimated for each timepoint. Pairwise comparisons versus baseline were adjusted for multiple testing using the Sidak method.

To explore potential effect modification in the primary combined endpoints, interaction terms were examined. For RCSMS, both time‐by‐age and time‐by‐asthma status interactions were tested. For ACSMS, a time‐by‐age interaction was assessed within the asthma subgroup.

All patients contributed baseline (T0) and first‐year (T1) assessments. Beyond T1, the number of observations at subsequent timepoints decreased because treatment duration varied across patients within the study period. Consequently, later timepoints (T2–T5) correspond to patients with longer treatment exposure rather than to losses to follow‐up. To provide distribution‐free confirmation of within‐subject changes, complementary non‐parametric analyses were performed for all six scores. Paired comparisons between baseline (T0) and first‐year follow‐up (T1) were conducted using the Wilcoxon signed‐rank test, and the corresponding effect size (*r*) was calculated. For patients with data available at three or more consecutive timepoints, within‐subject comparisons across time were assessed using the Friedman test. When significant, post hoc Nemenyi pairwise comparisons were performed. For Friedman analyses, effect size was expressed as Kendall's W. Long‐term within‐subject analyses were performed in available‐case subsets of patients with complete data at each follow‐up time point.

In addition, changes in the distribution of symptom severity categories and medication score categories were evaluated using Pearson's χ^2^ test, with effect size quantified using the Phi coefficient (*φ*). These categorical comparisons were performed for T0 versus T1, and for T0 versus later time points when applicable.

The interpretation of effect sizes followed established conventions for correlation‐based metrics, applying thresholds as follows: small = 0.10, moderate = 0.30 and large ≥ 0.50, for all r‐derived statistics, including Wilcoxon *r*, Phi (φ), and Kendall's W [21, 22]. Statistical significance was set at *α* < 0.05. Statistical analyses were conducted using IBM SPSS Statistics Version 20.0, XLSTAT Version 2025.1.3, or Epidat Version 4.2, and Microsoft Excel.

To assess safety ARs were descriptively summarized.

### 2.6 Ethical Considerations

The study was conducted in accordance with the principles of the Declaration of Helsinki and applicable data protection regulations (LOPDGDD 3/2018) [23]. As this was a retrospective and observational study, no influence was exerted on the investigator's prescription, and all data were obtained under routine clinical conditions, without any subjective bias for either patients or investigators. Given this design and the exclusive use of anonymized data, obtaining individual informed consent was not required. The study was approved by the Ethics Committee of Hospital Universitario Ntra. Sra. de Candelaria.

## 3 Results

### 3.1 Study Population

A total of 238 patients were identified in the clinical records. After applying the predefined eligibility criteria, 30 were excluded due to missing baseline or 1‐year symptom/medication data, absence of rhinoconjunctivitis symptoms at baseline, incomplete treatment duration, data outside the pre‐specified study window, or insufficient key variables for reliable evaluation. The final study cohort therefore comprised 208 patients.

### 3.2 Baseline Characteristics

Among the 208 included patients, the mean age was 23.8 years (SD 14.8; 95% CI 21.8–25.8; range 5–65 years). Males represented 53.8% of the cohort, and asthma was present in 59.6% of patients (*n* = 124/208).

Regarding age categories, 36 patients (17.3%) were children, 66 (31.7%) adolescents, and 106 (51.0%) adults. Therefore, the pediatric population (children and adolescents combined) accounted for 49.0% of the study cohort (*n* = 102/208). A significant association was observed between age category and asthma status, with asthma being more frequent in children (80.6%) than in adolescents (56.1%) or adults (54.7%) (*p* = 0.019). In contrast, asthma prevalence did not significantly differ between genders (*p* = 0.230).

The mean duration of allergen immunotherapy was 2.6 years. Assuming one injection per month, each patient received an estimated mean of 31.2 injections, and therefore, this corresponds to an estimated total of 6490 injections in the cohort.

### 3.3 Effectiveness Endpoint

To evaluate longitudinal changes while appropriately accounting for within‐subject correlation across repeated measurements, Mixed Models for Repeated Measures (MMRM) were fitted for all quantitative outcomes. Overall, clinically meaningful and statistically significant reductions in symptoms and medication use were observed throughout treatment.

#### 3.3.1 Rhinoconjunctivitis

When RCSMS was analyzed as the dependent variable, a highly significant main effect of time was observed (*F* (5, 544.975) = 212.499; *p* < 0.001), confirming a sustained reduction throughout follow‐up. Adjusted LS‐means decreased from 3.97 (SE 0.06) at T0 to 2.26 (SE 0.06) at T1 and 1.24 (SE 0.17) at T5, corresponding to relative reductions of 43.1% and 68.8%, respectively (Table 1; Figure 1a). All post‐baseline timepoints were significantly different from baseline after Sidak adjustment (*p* < 0.001). Pairwise comparisons indicated that the largest incremental reductions occurred between T0–T1 and T1–T2, whereas differences between later timepoints (T3–T4 and T4–T5) were not statistically significant, suggesting maintenance of benefit with a stabilization of the observed improvement from approximately year 3 onwards. Gender showed a significant association with adjusted RCSMS levels (*F* (1, 230.543) = 11.426; *p* = 0.001), whereas age and asthma status were not independently associated with overall RCSMS values (*p* > 0.05).

**TABLE 1:** Longitudinal adjusted outcomes for RCSMS and ACSMS estimated using mixed models for repeated measures (MMRM).

	T0	T1	T2	T3	T4	T5
RCSMS						
LS‐mean (SE)	3.97 (0.06)	2.26 (0.06)	1.68 (0.08)	1.35 (0.09)	1.08 (0.11)	1.24 (0.17)
95% CI	3.84–4.09	2.13–2.38	1.53–1.84	1.18–1.52	0.87–1.29	0.90–1.58
*n*	208	208	126	104	65	24
Δ versus T0	—	−1.71	−2.29	−2.62	−2.89	−2.73
% reduction	—	43.1	57.6	66.0	72.8	68.8
ACSMS						
LS‐mean (SE)	2.74 (0.09)	1.36 (0.09)	0.80 (0.11)	0.64 (0.13)	0.48 (0.16)	0.46 (0.22)
95% CI	2.56–2.93	1.18–1.54	0.59–1.02	0.39–0.89	0.17–0.79	0.02–0.89
*n*	124	124	73	61	36	17
*Δ* versus T0	—	−1.38	−1.94	−2.10	−2.26	−2.28
% reduction	—	50.5	70.7	76.7	82.4	83.3

*Note:* LS‐means were estimated from MMRM with time (categorical) as fixed effect. For rhinoconjunctivitis outcomes, models included age, gender and asthma status as fixed effects. For asthma outcomes, analyses were restricted to patients with asthma and included age and gender as fixed effects. *Δ* versus T0 represents the adjusted mean difference relative to baseline. All post‐baseline comparisons versus T0 were statistically significant (Sidak‐adjusted *p* < 0.001). The decrease in sample size beyond T1 reflects differences in treatment duration within the study period; later timepoints represent subsets of patients with longer treatment exposure and do not indicate attrition or loss to follow‐up.

Abbreviations: Δ = adjusted mean difference, ACSMS = Asthma Combined Symptom and Medication Score, CI = confidence interval, LS‐mean = least‐squares mean, RCSMS = Rhinoconjunctivitis Combined Symptom and Medication Score, SE = standard error.

**FIGURE 1:**
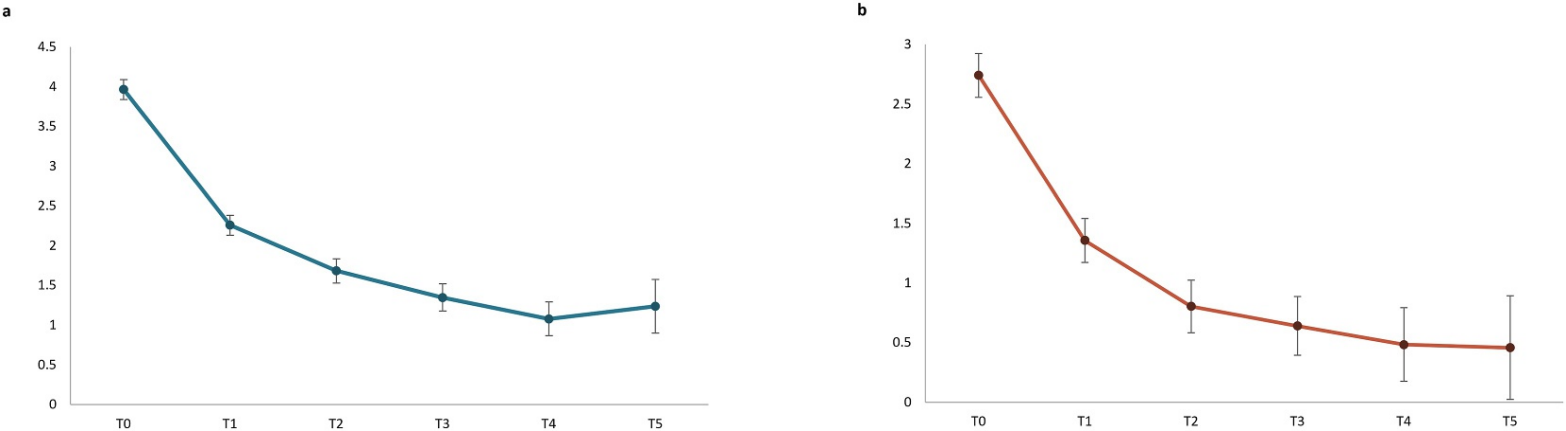
Adjusted LS‐means (95% CI) for (a) RCSMS and (b) ACSMS estimated from mixed models for repeated measures (MMRM) adjusted for age and gender. All post‐baseline timepoints were significantly different from baseline (Sidak‐adjusted *p* < 0.001).

To explore potential effect modification, interaction terms were incorporated into the model. The time‐by‐age interaction was not statistically significant (*F* (5, 546.196) = 1.285; *p* = 0.269), indicating that the magnitude and trajectory of RCSMS reduction over time did not differ according to patient group (pediatrics vs. adults). Similarly, the time‐by‐asthma status interaction was not significant (*F* (5, 544.962) = 0.246; *p* = 0.942), demonstrating that longitudinal improvements in rhinoconjunctivitis outcome were comparable between patients with and without asthma. These findings indicate that the observed longitudinal changes were consistent across clinically relevant subgroups.

Consistent findings were observed for the symptoms and medication scores analyzed separately (Figure 2a; Supporting Information S1: Appendix, Table S1‐S2). For RSS, a significant main effect of time was identified (*F* (5, 539.610) = 307.664; *p* < 0.001). Adjusted LS‐means declined from 1.86 (SE 0.03; 95% CI 1.79–1.92) at T0 to 0.91 (SE 0.03; 95% CI 0.84–0.97) at T1 and reaching 0.43 (SE 0.08; 95% CI 0.27–0.59) at T5. Relative reductions versus baseline were 51.3% at T1, 67.5% at T2, 76.1% at T3, 79.5% at T4 and 77.1% at T5. All post‐baseline comparisons versus T0 were statistically significant (*p* < 0.001). For RMS, the MMRM similarly demonstrated a significant longitudinal effect (*F* (5, 562.551) = 95.719; *p* < 0.001). Adjusted LS‐means decreased from 2.11 (SE 0.04; 95% CI 2.02–2.19) at baseline to 1.35 (SE 0.04; 95% CI 1.27–1.44) at T1, and to 0.81 (SE 0.12; 95% CI 0.57–1.05) at T5. Relative reductions were 35.8% at T1, 48.5% at T2, 56.9% at T3, 66.7% at T4 and 61.6% at T5. Reductions at all post‐baseline visits were statistically significant compared with T0. Overall, symptom improvement occurred early, while medication reductions followed a more gradual pattern; both domains were maintained long term.

**FIGURE 2:**
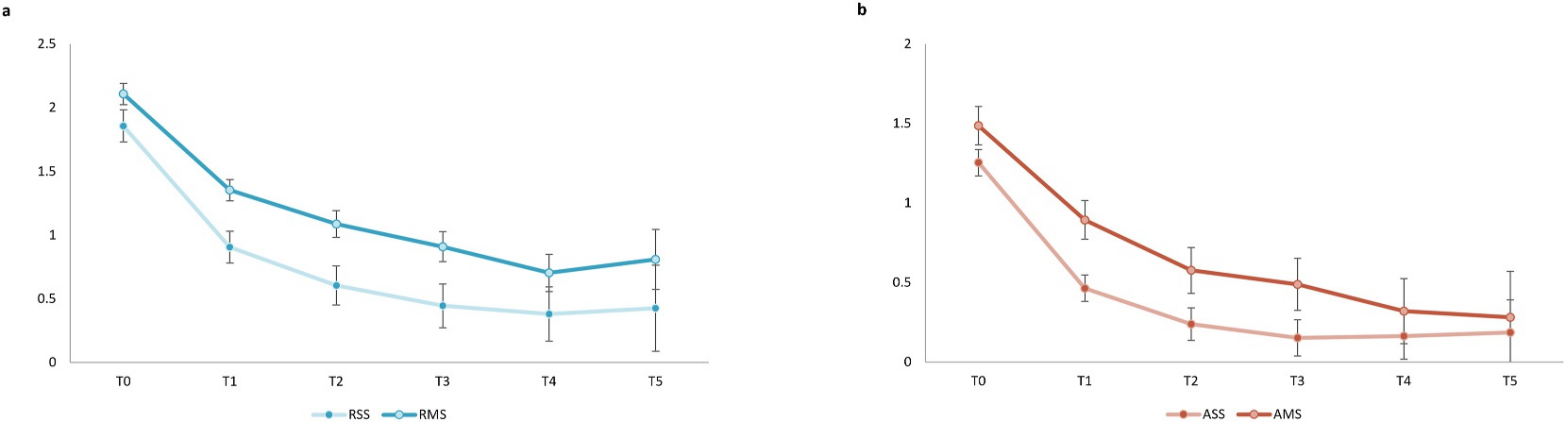
Adjusted LS‐means (95% CI) over time for (a) RSS and RMS and (b) ASS and AMS estimated from mixed models for repeated measures (MMRM) adjusted for age and gender.

#### 3.3.2 Asthma

In the asthma subgroup, MMRM analyses confirmed a robust longitudinal treatment effect for the combined asthma endpoint. When ACSMS was analyzed as the dependent variable, a significant main effect of time was observed (*F* (5, 317.921) = 79.308; *p* < 0.001), indicating sustained improvement across follow‐up. Adjusted LS‐means decreased from 2.74 (SE 0.09; 95% CI 2.56–2.93) at T0 to 1.36 (SE 0.09; 95% CI 1.18–1.54) at T1 and 0.46 (SE 0.22; 95% CI 0.02–0.89) at T5, corresponding to relative reductions of 50.5% and 83.3%, respectively (Table 1; Figure 1b). All post‐baseline comparisons versus T0 remained statistically significant after Sidak adjustment (*p* ≤ 0.001). The majority of the adjusted improvement accrued during the first 2 years, followed by smaller incremental changes and a plateau thereafter.

Age showed a significant independent association with adjusted ACSMS levels (F(1, 126.773) = 12.973; *p* < 0.001), reflecting differences in overall asthma burden across age groups, whereas gender was not significantly associated (*p* = 0.806).

Regarding potential effect modification, the time‐by‐age interaction was not statistically significant (*F* (5, 315.149) = 0.697; *p* = 0.626), indicating that the magnitude and trajectory of longitudinal improvement were comparable between pediatric and adult patients despite baseline differences in severity.

Consistent patterns were observed for the individual asthma components (Figure 2b; Supporting Information S1: Appendix, Table S1‐S2). For ASS, a significant main effect of time was identified (*F* (5, 313.877) = 96.747; *p* < 0.001), with adjusted LS‐means decreasing from 1.25 (SE 0.04; 95% CI 1.17–1.34) at baseline to 0.46 (SE 0.04; 95% CI 0.38–0.55) at T1 and 0.19 (SE 0.11; 95% CI −0.02–0.39) at T5. Relative reductions reached 63.0% at T1 and exceeded 85% from T3 onwards. Similarly, AMS showed a significant longitudinal effect (*F* (5, 320.870) = 39.121; *p* < 0.001). Adjusted LS‐means declined from 1.49 (SE 0.06; 95% CI 1.37–1.61) at T0 to 0.89 (SE 0.06; 95% CI 0.77–1.02) at T1 and 0.28 (SE 0.15; 95% CI −0.01 to 0.57) at T5, corresponding to reductions of 39.9% and 81.1%, respectively. Overall, asthma‐related outcomes showed a pronounced early response followed by progressive consolidation and long‐term stabilization, with parallel reductions in both symptom burden and medication requirements.

The number of observations at later annual timepoints decreased after T1 (RCSMS: *n* = 208 at T0 and T1, 126 at T2, 104 at T3, 65 at T4, and 24 at T5; ACSMS: *n* = 124 at T0 and T1, 73 at T2, 61 at T3, 36 at T4, and 17 at T5). This decrease reflects differences in treatment duration within the study period, rather than treatment discontinuation or loss to follow‐up. Accordingly, later timepoints represent subsets of patients with longer treatment exposure.

### 3.4 Complementary Non‐Parametric Analyses

To further assess robustness independently of distributional assumptions, complementary non‐parametric analyses were performed using the Wilcoxon signed‐rank test for paired comparisons (T0–T1) and the Friedman test for repeated measures across available follow‐up (T0–Tx).

Regarding rhinoconjunctivitis, median RCSMS values decreased significantly from 4.0 (IQR 3.5–4.7) at baseline to 2.2 (IQR 1.7–2.8) after the first year of treatment (45.8% reduction; *p* < 0.001; large effect size), with sustained improvements across subsequent follow‐up timepoints (Table 2; Figure 3a). Median RSS decreased from 1.8 (IQR 1.5–2.2) to 0.8 (IQR 0.7–1.2) at T1, with progressive decline to 0.3 (IQR 0.2–0.5) at T5 (*p* < 0.001; large effect size) (Figure 4a; Supporting Information S1: Appendix, Table S3). These quantitative reductions were mirrored by categorical severity shifts, with the proportion of patients presenting moderate‐to‐severe rhinoconjunctivitis decreasing from 95.2% at baseline to 28.3% at T1 and to < 5% at long‐term follow‐up, while no patients remained in the severe category at T5 (Table 3). In parallel, RMS decreased from 2.0 (IQR 2.0–2.0) at baseline to 1.0 (IQR 1.0–2.0) at T1 (*p* < 0.001), and remained reduced thereafter, with a shift from intranasal corticosteroid‐based regimens and oral corticosteroid rescue toward antihistamines only or no medication during follow‐up (Table 4).

**TABLE 2:** Evolution of combined symptom and medication scores across the study period.

	T0	T1	T2	T3	T4	T5
RCSMS						
Mean (SD)	3.9 (0.8)	2.2 (1.0)	1.7 (0.9)	1.3 (0.9)	1.0 (0.9)	1.2 (0.7)
Median (Q1, Q3)	4.0 (3.5, 4.7)	2.2 (1.7, 2.8)	1.6 (1.3, 2.0)	1.3 (1.0, 1.5)	1.2 (0.2, 1.4)	1.3 (1.0, 1.5)
*n*	208	208	126	104	65	24
%Reduction versus T0_Median_	—	45.8	60.4	66.7	70.8	66.7
*p* (wilcoxon T0‐T1)	—	< 0.0001^a^	—	—	—	—
*p* (friedman T0‐T5)	—	—	< 0.0001^b^	< 0.0001^c^	< 0.0001^d^	< 0.0001^e^
Effect size	—	0.84^a^	0.82^b^	0.78^c^	0.74^d^	0.71^e^
Interpretation effect size	—	Large^a^	Large^b^	Large^c^	Large^d^	Large^e^
ACSMS						
Mean (SD)	2.7 (1.2)	1.4 (1.1)	0.8 (0.9)	0.6 (0.9)	0.5 (0.7)	0.7 (0.9)
Median (Q1, Q3)	3.0 (1.5, 3.8)	1.0 (0.5, 2.0)	0.8 (0.1, 1.0)	0.5 (0.0, 0.8)	0.0 (0.0, 1.0)	0.0 (0.0, 1.5)
*n*	124	124	73	61	36	17
%Reduction versus T0 _Median_	—	66.7	75.0	83.3	100.0	100.0
*p* (Wilcoxon)	—	< 0.0001^a^	—	—	—	—
*p* (Friedman)	—	—	< 0.0001^b^	< 0.0001^c^	< 0.0001^d^	< 0.0001^e^
Effect size	—	0.80^a^	0.68^b^	0.69^c^	0.69^d^	0.59^e^
Interpretation effect size	—	Large^a^	Large^b^	Large^c^	Large^d^	Large^e^

*Note:* Effect sizes correspond to the Wilcoxon signed‐rank test (Wilcoxon *r*; T0‐T1) or the Friedman test (Kendall's W; T0‐T5) within each respective subgroup.

Abbreviations: ACSMS = Asthma Symptom and Medication Score, RCSMS: Rhinoconjunctivitis Symptom and Medication Score, SD = Standard Deviation, Q1 = First quartile, Q3 = Third quartile.

^a^ Comparison performed in patients with available data at baseline and year 1.

^b^ Comparison performed in patients with available data at baseline and year 2.

^c^ Comparison performed in patients with available data at baseline and year 3.

^d^ Comparison performed in patients with available data at baseline and year 4.

^e^ Comparison performed in patients with available data at baseline and year 5.

**FIGURE 3:**
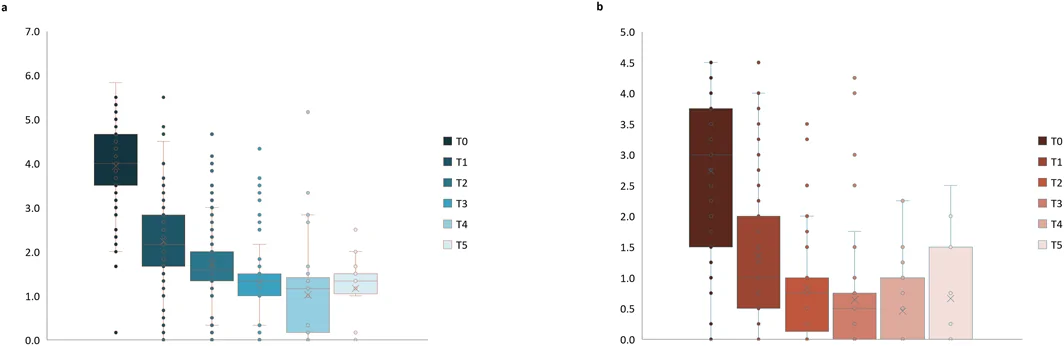
Distribution of Combined Symptom and Medication Scores of (a) Rhinoconjunctivitis (RCSMS) and (b) Asthma (ACSMS), across the study period. Boxplots show the median and interquartile range, with whiskers indicating the spread of the data.

**FIGURE 4:**
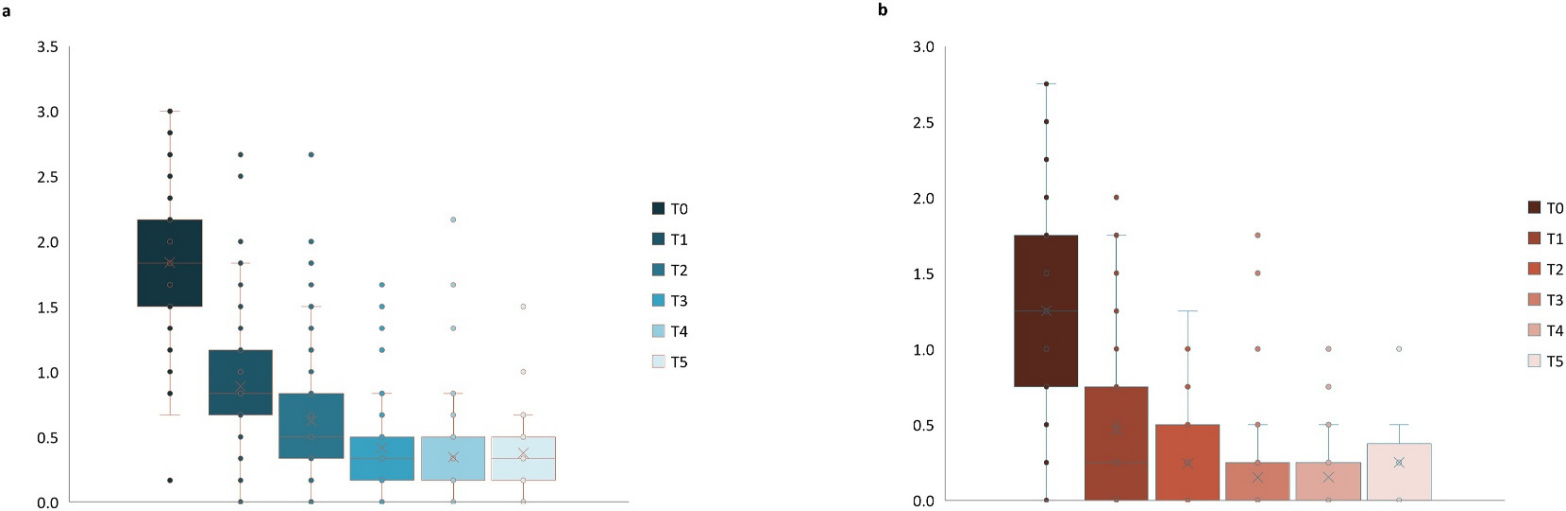
Distribution of Symptom Scores of (a) Rhinoconjunctivitis (RSS) and (b) Asthma (ASS), across the study period.

**TABLE 3:** Distribution of symptom severity categories over the study period.

	T0	T1	T2	T3	T4	T5
RSS classification, *n* (%)						
No symptoms	0 (0)	7 (3.4)	11 (8.7)	18 (17.3)	14 (21.6)	5 (20.8)
Mild	10 (4.8)	142 (68.3)	99 (78.6)	77 (74)	47 (72.3)	18 (75)
Moderate	137 (65.9)	57 (27.4)	15 (11.9)	9 (8.7)	3 (4.6)	1 (4.2)
Severe	61 (29.3)	2 (0.9)	1 (0.8)	0 (0)	1 (1.5)	0 (0)
*n*	208	208	126	104	65	24
ASS classification, *n* (%)						
No symptoms	3 (2.4)	43 (34.7)	38 (52.8)	44 (72.1)	26 (72.2)	10 (58.8)
Mild	53 (42.8)	68 (54.8)	33 (45.8)	15 (24.6)	10 (27.8)	7 (41.2)
Moderate	63 (50.8)	13 (10.5)	1 (1.4)	2 (3.3)	0 (0)	0 (0)
Severe	5 (4)	0 (0)	0 (0)	0 (0)	0 (0)	0 (0)
*n*	124	124	72	61	36	17

Abbreviations: ASS = Asthma Symptom Score, RSS = Rhinoconjunctivitis Symptom Score.

**TABLE 4:** Distribution of patients across medication scores during the study period.

		T0	T1	T2	T3	T4	T5
Score	Interpretation	RMS, *n* (%)					
0	No medication	1 (0.5)	20 (9.6)	13 (10.3)	23 (22.1)	26 (40)	5 (20.8)
1	Oral/topical antihistamines	21 (10.1)	103 (49.5)	88 (69.8)	70 (67.3)	35 (53.8)	19 (79.2)
2	Intranasal corticosteroids w/ or w/o antihistamines	142 (68.3)	78 (37.5)	23 (18.3)	10 (9.6)	3 (4.6)	0 (0)
3	Oral corticosteroids w/ or w/o antihistamines	44 (21.2)	7 (3.4)	2 (1.6)	1 (1.0)	1 (1.5)	0 (0)
*n*		208	208	126	104	65	24

**Score**	**Interpretation**	**AMS, *n* (%)**					
0	No medication	3 (2.4)	19 (15.3)	20 (27.4)	27 (44.3)	23 (63.9)	10 (58.8)
0.5	SABA on demand	33 (26.6)	54 (43.5)	35 (47.9)	23 (37.7)	7 (19.4)	2 (11.8)
1	Low‐dose IC/LTRA	11 (8.9)	17 (13.7)	10 (13.7)	2 (3.3)	3 (8.3)	3 (17.6)
1.5	Low‐dose IC + LABA/Medium‐dose IC w/ or w/o LTRA w/ or w/o SABA on demand	22 (17.7)	14 (11.3)	3 (4.1)	6 (9.8)	3 (8.3)	2 (11.8)
2	Medium‐dose IC + LABA w/ or w/o LTRA w/ or w/o SABA on demand	28 (22.6)	9 (7.3)	2 (2.7)	0 (0)	0 (0)	0 (0)
2.5	High‐dose IC + LABA w/ or w/o LTRA w/ or w/o SABA on demand	27 (21.8)	11 (8.9)	3 (4.1)	3 (4.9)	0 (0)	0 (0)
3	High‐dose IC + LABA + SC w/ or w/o LTRA w/ or w/o SABA on demand	0 (0)	0 (0)	0 (0)	0 (0)	0 (0)	0 (0)
*n*		124	124	73	61	36	17

Abbreviations: AMS = Asthma Medication Score, IC = Inhaled Corticosteroids, LABA = Low‐dose IC + Long‐Acting β‐2 Agonists, LTRA = Leukotriene Receptor Antagonists, RMS = Rhinoconjunctivitis Medication Score, SABA = Short‐Acting β‐2 Agonists, w/ = with, w/o = without.

Asthma outcomes showed a comparable pattern. Median ACSMS declined from 3.0 (IQR 1.5–3.8) at baseline to 1.0 (IQR 0.5–2.0) at T1 (66.7% reduction; *p* < 0.0001; large effect size), with further improvement over time and median values reaching 0.0 from T4 onwards (*p* < 0.001; large effect size) (Table 2; Figure 3b). Median ASS decreased from 1.3 (IQR 0.8–1.8) to 0.3 (IQR 0.0–0.8) at T1 and reached 0.0 from T2 onwards in most patients (*p* < 0.001) (Figure 4b; Supporting Information S1: Appendix, Table S3), accompanied by a progressive increase in asymptomatic patients and a marked reduction in moderate‐to‐severe symptom categories (*p* ≤ 0.001) (Table 3). Finally, median AMS declined from 1.5 (IQR 0.5–2.0) at T0 to 0.5 (IQR 0.5–1.5) at T1 and remained low thereafter, with a shift toward lower‐intensity controller therapy or no medication at later timepoints (*p* < 0.05) (Table 4).

Descriptive summaries and boxplots by timepoint for all 6 scores are provided in the Supporting Information S1: Appendix (Tables S4‐S41, Figures S1‐S30).

Importantly, the magnitude and direction of improvement remained consistent in patients with longer follow‐up durations despite the progressive reduction in sample size over time, supporting the robustness of the observed effects.

### 3.5 Safety Endpoint

Three non‐serious adverse reactions were reported during the study; of these, only one was clinically relevant, and occurred in a 7‐year‐old male patient, who had completed 1 year of AIT. The reaction was a mild, systemic reaction (persistent cough; WAO grade 1) classified as non‐serious, with possible causality, managed with pharmacological treatment only, and resolved completely without requiring modification of the immunotherapy regimen. No hospitalization, life‐threatening reactions, disability, congenital anomaly, or other unexpected safety issues were reported.

Considering the estimated total exposure of 6490 injections administered in the study population, the incidence of clinically relevant adverse reactions was 0.48% per patient (*n* = 1/208) and 0.015% per injection (*n* = 1/6490). Among patients with asthma, the incidence of ARs was 0.82% (*n* = 1/124), and per age group, incidence in children was 2.78% (*n* = 1/36).

## 4 Discussion

In this real‐world study, subcutaneous immunotherapy with a mixture of individually glutaraldehyde polymerized extracts of *Dermatophagoides*/*B. tropicalis* was associated with substantial and sustained clinical improvement in patients with allergic rhinoconjunctivitis and asthma. The key finding is the marked and durable decrease in combined symptom‐medication scores for both rhinoconjunctivitis (RCSMS) and asthma (ACSMS). Current EAACI recommendations consider combined scores the preferred primary endpoint in allergen immunotherapy because they capture both symptom burden and pharmacological needs in a single measure [15]. In our cohort, adjusted LS‐mean RCSMS decreased from 3.97 at baseline to 2.26 after the first year of treatment and to 1.24 at long‐term follow‐up, corresponding to relative reductions of 43.1% and 68.8%, respectively. Similarly, ACSMS decreased from 2.74 at baseline to 1.36 at 1 year and to 0.46 at the longest follow‐up, corresponding to reductions of 50.5% and 83.3%. These improvements were already evident after the first year and remained stable or further improved thereafter, indicating sustained clinical improvement during SCIT. However, given the observational design and absence of a parallel control group, causality cannot be established and a disease‐modifying effect cannot be confirmed [12, 24]. The wider confidence intervals observed at later timepoints likely reflect the reduced sample size at extended follow‐up, but the direction and magnitude of benefit remained stable.

Recent studies have proposed a minimal clinically important difference (MCID) of approximately −0.21 to −0.23 points for the EAACI‐recommended CSMS [25]. In our cohort, the adjusted LS‐mean reduction in RCSMS at T1 was −1.71 points, clearly exceeding these thresholds and supporting the clinical relevance of the observed change, although these estimates were derived from studies using different allergens and treatment platforms. While a formal MCID has not yet been established for asthma CSMS, the magnitude of change observed in ACSMS (−1.38 points at year one) was similarly large, further reinforcing the clinical relevance of the observed change.

An additional relevant finding was the absence of significant time‐by‐age and time‐by‐asthma interactions in the longitudinal models. This indicates that the magnitude and trajectory of improvement in rhinoconjunctivitis outcomes were similar across age groups and were not modified by the presence of concomitant asthma. These findings suggest that the clinical benefits of SCIT were broadly consistent across pediatric and adult patients and were comparable in patients with isolated rhinoconjunctivitis and those with combined respiratory allergic disease [26, 27]. Importantly, rhinoconjunctivitis improvement was comparable in patients with and without asthma, addressing the clinically relevant question of whether concomitant asthma attenuates response.

Complementary non‐parametric analyses were consistent with the MMRM findings and added clinically intuitive evidence based on medians and severity categories. Median RCSMS and ACSMS decreased significantly at T1 and across available follow‐up with large effect sizes, confirming the direction and magnitude of the longitudinal improvements observed in the primary MMRM analyses. Importantly, severity shifted in parallel with score reductions: the proportion of patients with moderate‐to‐severe rhinoconjunctivitis symptoms decreased sharply after year 1 and severe disease was no longer observed at year 5, while asthma symptom categories similarly moved toward asymptomatic or mild states after treatment initiation. Beyond statistical significance and median‐based analyses, the magnitude of change observed in the present study also supports the robustness of the treatment effect. Effect size metrics provide an estimate of the magnitude of within‐patient improvement that is less dependent on sample size and therefore complement conventional hypothesis testing. In this context, the large effect sizes observed in the non‐parametric analyses further support the magnitude and clinical relevance of the observed improvements [21, 22].

The magnitude of change in combined scores was broadly consistent with that reported in other real‐world series of HDM SCIT. In a Colombian prospective study using a glutaraldehyde‐modified three‐mite mixture, Cardona‐Villa et al. reported CSMS reductions of around 40% at 12 months [9]. More recently, a real‐world pediatric study in Colombia with polymerized HDM extracts (Alxoid) confirmed clinically meaningful reductions in CSMS from the first year of treatment onwards, supporting the effectiveness of allergoid‐based SCIT in routine practice [28]. Placebo‐controlled and observational studies with depigmented‐polymerized HDM extracts have described CSMS decreases in the range of 50%–60% after 2–3 years of treatment [14, 29]. The adjusted reductions observed here for RCSMS and ACSMS were within the range of these reported effects. However, these indirect comparisons should be interpreted cautiously because of differences. despite differences in study design, patient populations, and outcome assessment.

Rhinoconjunctivitis and asthma outcomes, when analyzed separately as symptom and medication scores, provide further support for the robustness of the observed treatment effect. RSS and ASS alone showed substantial and sustained reductions, with median decreases of more than 50% after the first year and reaching very low values at later timepoints. Medication scores also declined progressively over time, reflecting a gradual reduction in pharmacological requirements. This pattern was characterized by early symptom improvement followed by more gradual reductions in medication scores, a clinically plausible trajectory in which improved disease control enables progressive step‐down of pharmacotherapy. Similar longitudinal patterns of sustained symptom control with HDM SCIT have been reported in long‐term real‐world cohorts and observational studies [30, 31].

The observed reduction in medication use constitutes a clinically relevant outcome in its own right. Long‐term pharmacological treatment is associated with cumulative costs, adherence challenges, and potential adverse effects, particularly with corticosteroid‐based therapies. Previous real‐world studies have shown that HDM SCIT enables step‐down of controller therapy while maintaining stable clinical control [32, 33]. The shift toward lower‐intensity treatment or complete medication withdrawal may translate into simplified disease management, reduced treatment burden, and improved long‐term sustainability of care [34].

Notably, asthma outcomes in the present study are particularly favourable, with marked improvements in ASS and ACSMS and a progressive decline in the proportion of patients requiring high‐intensity maintenance therapy. These observations align with meta‐analyses and long‐term cohorts showing that HDM SCIT improves asthma control, reduces exacerbations, and facilitates step‐down of inhaled corticosteroids and other controllers [12, 35, 36, 37]. These findings are also consistent with recent real‐world pediatric data using polymerized HDM extracts, which demonstrated significant improvements not only in rhinitis outcomes but also in asthma control and medication scores [28]. The concordance between adult and pediatric real‐world data supports the applicability of allergoid‐based SCIT across age groups, a particularly relevant aspect given the early onset of mite sensitization in regions with perennial exposure. The depth and stability of the reductions observed in asthma‐related scores support the role of SCIT as a disease‐modifying intervention in this population.

An additional distinctive feature of this study is the inclusion of *B*. *tropicalis* in the vaccine composition. In the Canary Islands and other subtropical or transitional regions, *B. tropicalis* sensitization is highly prevalent and frequently coexists with sensitization to *Dermatophagoides* spp., contributing significantly to persistent rhinoconjunctivitis and asthma symptoms [3, 4, 5, 8, 38, 39]. In such settings, immunotherapy based only on *D. pteronyssinus*/*D. farinae* may provide incomplete allergenic coverage. Clinical data with *B. tropicalis*‐containing HDM formulations are still relatively limited but increasingly supportive. SCIT using combined *D. pteronyssinus*/*D. farinae*/*B. tropicalis* mixtures has demonstrated significant improvements in CSMS and asthma control with good tolerability in Latin American real‐world cohorts [9]. In addition, a recent prospective study evaluating the diagnostic and therapeutic relevance of *B. tropicalis* using nasal challenge testing (NCT) demonstrated that AIT significantly increased tolerance thresholds in NCT‐positive patients, further supporting the role of *B. tropicalis* as a clinically relevant therapeutic target [40]. The present results add to this evidence by showing that treatment with a triple‐mite, glutaraldehyde‐polymerized preparation was associated with substantial and durable reductions in both rhinoconjunctivitis‐ and asthma‐related combined scores under routine clinical conditions in a region where *B. tropicalis* is clearly clinically relevant. To our knowledge, this represents one of the largest real‐world cohorts evaluating allergen immunotherapy including *B. tropicalis*.

Safety findings in this study are also reassuring. Only one clinically significant non‐serious AR was reported corresponding to 0.48% of patients and 0.015% of injections, with no serious reactions, no hospitalizations, and no treatment discontinuations. These figures appear favourable in relation to historical data for SCIT with native extracts and are consistent with those reported for other glutaraldehyde‐modified allergoid preparations [41, 42], although direct comparisons are limited by differences in study design, allergen preparations, duration of exposure, and safety‐reporting procedures. In a large multicenter safety study of Inmunotek's glutaraldehyde‐modified Clustoid preparations, Guzmán‐Fulgencio et al. reported systemic reaction rates of 0.06% per injection, all mild or moderate [41]. Similarly low rates have been documented for other Inmunotek's formulations, such as Clustoid *Alternaria alternata* allergoid [43], or Alxoid *D. pteronyssinus*/*D. farinae* [28], and for HDM allergoids in randomized and observational studies [44, 45, 46]. Within this context, the very low incidence of systemic reactions and the absence of severe events observed here are consistent with the established safety profile of depigmented‐polymerized extracts and support a favourable benefit–risk balance for this glutaraldehyde‐polymerized *Dermatophagoides*/*B. tropicalis* extract in everyday practice.

Several limitations should be considered. First, the retrospective design and absence of a parallel control group limit causal inference. Second, symptom and medication scores were reconstructed from routine clinical records rather than collected prospectively using standardized diaries, which may introduce measurement variability and potential between‐center differences in the completeness of source documentation. In addition, because safety data were obtained retrospectively from routine clinical records, systematic recording of all minor, non‐clinically relevant local reactions could not be guaranteed. Finally, follow‐up beyond the first year was not uniform because treatment duration differed across patients; consequently, later timepoints reflect patients with longer treatment duration and therefore involve smaller available‐case subsets, although the direction and magnitude of change were consistent across timepoints.

## 5 Conclusion

This real‐world study found that triple‐mite subcutaneous immunotherapy with glutaraldehyde‐polymerized extracts of *Dermatophagoides* spp. and *B*. *tropicalis* was associated with sustained improvements in symptoms and medication use for allergic rhinoconjunctivitis and asthma. These improvements were observed from the first year and maintained over long‐term follow‐up, with consistently large effect sizes. The treatment showed a favourable safety profile, with only one clinically relevant non‐serious adverse reaction. Overall, these findings support a positive benefit–risk balance for this triple‐mite SCIT, particularly in regions where *Dermatophagoides* spp. and *B. tropicalis* coexist.

## Author Contributions

C.G.C., A.C., G.L.H.S., M.G.A., E.R.P., J.A.M.T., J.C.G.R. performed the review of the clinical history of patients, the completion of the data booklet, and reviewed the manuscript. M.J.M. was responsible for the safety review. M.C., Y.R.G., and J.D.G. were responsible for the conception and design of the study. M.C. performed the statistical analysis and reviewed the manuscript. Y.R.G. reviewed the manuscript. J.D.G. performed the statistical analysis, interpreted the results, and wrote the manuscript. All authors approved the final version.

## Ethics Statement

The study was conducted in accordance with the principles of the Declaration of Helsinki and applicable data protection regulations (LOPDGDD 3/2018). As this was a retrospective and observational study, no influence was exerted on the investigator's prescription, and all data were obtained under routine clinical conditions, without any subjective bias for either patients or investigators. Given this design and the exclusive use of anonymized data, obtaining individual informed consent was not required. The study was approved by the Ethics Committee of Hospital Universitario Ntra. Sra. de Candelaria. The study was registered at ClinicalTrials.gov (Identifier: NCT07427576).

## Conflicts of Interest

M.C. is shareholder and employee of Inmunotek S.L. M.J.M., Y.R.G. and J.D.G. are employees of Inmunotek S.L. J.C.G.R. reports grants for his institution from Inmunotek S.L. during the conduct of the study; grants from AstraZeneca; and personal fees for lectures or educational activities from AstraZeneca, L.E.T.I. and G.S.K. C.G.C, G.L.H.S., E.R.P., M.G.A., A.C., and J.A.M.T report grants for their institution from Inmunotek S.L. during the conduct of the study.

## Supporting information


Supporting Information S1

## Data Availability

The data that support the findings of this study are available on request from the corresponding author. The data are not publicly available due to privacy or ethical restrictions.
