# Supplementary material for: Real‐World Effectiveness and Safety of Triple‐Mite SCIT With Dermatophagoides and Blomia tropicalis
Source: Clin Transl Allergy. 2026 Oct 10;16(10):e70217. doi: 10.1002/clt2.70217 (PMC13653539; doi:10.1002/clt2.70217)
Supplement: Supplementary file 1 [file CLT2-16-e70217-s001.pdf]

SUPPLEMENTAL APPENDIX

This supplementary appendix provides additional analyses supporting the main results. It includes complementary mixed models for repeated measures (MMRM) analyses for the individual symptom and medication scores—Rhinoconjunctivitis Symptom Score (RSS), Rhinoconjunctivitis Medication Score (RMS), Asthma Symptom Score (ASS), and Asthma Medication Score (AMS).

Descriptive tables and boxplots from non-parametrical analyses are also provided for subgroups of patients with complete follow-up at each time point for Rhinoconjunctivitis Symptom Score (RSS), Asthma Symptom Score (ASS), Rhinoconjunctivitis Medication Score (RMS), Asthma Medication Score (AMS), Rhinoconjunctivitis Combined Symptom and Medication Score (RCSMS), and Asthma Combined Symptom and Medication Score (ACSMS).

Figure S1. Box plot of the RSS at baseline and after one year of immunotherapy. 9
Figure S2. Box plot of the RSS at baseline, after one year, and after two years of immunotherapy in patients with complete two-year AIT data. 9
Figure S3. Box plot of the RSS at baseline, after one year, two years, and three years of immunotherapy in patients with complete three-year AIT data. 10
Figure S4. Box plot of the RSS at baseline and after up to four years of immunotherapy, in patients with complete four-year AIT data. 10
Figure S5. Box plot of the RSS from baseline to five years of immunotherapy, in patients with complete five-year AIT data. 11
Figure S6. Box plot of the ASS at baseline and after one year of immunotherapy. 11
Figure S7. Box plot of the ASS at baseline, and after one and two years of immunotherapy in patients with complete two-year AIT data. 12
Figure S8. Box plot of the ASS at baseline, and after one, two, and three years of immunotherapy in patients with complete three-year AIT data. 12
Figure S9. Box plot of the ASS at baseline and after one to four years of immunotherapy in patients with complete four-year AIT data. 13
Figure S10. Box plot of the ASS from baseline to five years of immunotherapy, in patients with complete five-year AIT data. 13
Figure S11. Distribution of patients according to RMS at baseline and after one year of treatment. 14
Figure S12. Distribution of patients according to RMS at baseline and after one and years of treatment. 16
Figure S13. Distribution of patients according to RMS at baseline and after up to three years of treatment in patients with complete three-year AIT data. 18
Figure S14. Distribution of patients according to RMS at baseline and after up to four years of treatment in patients with complete four-year AIT data. 20
Figure S15. Distribution of patients according to RMS at baseline and after up to five years of treatment in patients with complete five-year AIT data. 22
Figure S16. Distribution of patients according to AMS at baseline and after one year of AIT. 24

Figure S17. Distribution of patients according to AMS at baseline and after one and two years of AIT, in patients with complete two-year AIT data.. 26
Figure S18. Distribution of patients according to AMS at baseline and after up to three years of AIT in patients with complete three-year AIT data. 28
Figure S19. Distribution of patients according to AMS at baseline and after up to four years of AIT in patients with complete four-year AIT data. 30
Figure S20. Distribution of patients according to AMS at baseline and after up to five years of AIT in patients with complete five-year AIT data. 32
Figure S21. Evolution of the RCSMS during the first year of AIT. 34
Figure S22. Evolution of the RCSMS over two years of AIT. 36
Figure S23. Evolution of the RCSMS over three years of AIT....................................... 38
Figure S24. Evolution of the RCSMS over four years of AIT... 40
Figure S25. Evolution of the RCSMS over five years of AIT.. 42
Figure S26. Evolution of the ACSMS during the first year of AIT. 44
Figure S27. Evolution of the ACSMS over two years of AIT. 46
Figure S28. Evolution of the ACSMS over three years of AIT....................................... 48
Figure S29. Evolution of the ACSMS over four years of AIT... 50
Figure S30. Evolution of the ACSMS over five years of AIT.. 52

Table S1. Longitudinal adjusted outcomes for RSS and RMS, estimated using mixed models for repeated measures (MMRM)... 5
Table S2. Longitudinal adjusted outcomes for ASS and AMS, estimated using mixed models for repeated measures (MMRM)... 6
Table S3. Evolution of Symptom Scores across the study period.................................. 7
Table S4. Distribution of patients across RMS levels and statistical comparison between baseline and one year of AIT. 15
Table S5. Descriptive statistics of the RMS in the subset of patients who completed two years of AIT. 16
Table S6. Distribution of patients across RMS levels and statistical comparison between baseline and follow-up at one and two years of AIT... 17
Table S7. Descriptive statistics of the RMS in the subset of patients who completed three years of AIT. 18
Table S8. Distribution of patients across RMS levels and statistical comparison between baseline and follow-up at one, two and three years of AIT... 19
Table S9. Descriptive statistics of the RMS in the subset of patients who completed four years of AIT. 20
Table S10. Distribution of patients across RMS levels and statistical comparison between baseline and follow-up at one, two, three and four years of AIT. 21
Table S11. Descriptive statistics of the RMS in the subset of patients who completed five years of AIT. 22
Table S12. Distribution of patients across RMS levels and statistical comparison between baseline and follow-up at one, two, three, four and five years of AIT. 23
Table S13. Distribution of patients across AMS levels and statistical comparison between baseline and follow-up at one year of AIT. 25
Table S14. Descriptive statistics of the AMS in the subset of patients who completed two years of AIT. 26

Table S15. Distribution of patients across AMS levels and statistical comparison between baseline and follow-up at one and two years of AIT. 27
Table S16. Descriptive statistics of the AMS in the subset of patients who completed three years of AIT. 28
Table S17. Distribution of patients across AMS levels and statistical comparison between baseline and follow-up at one, two and three years of AIT. 29
Table S18. Descriptive statistics of the AMS in the subset of patients who completed four years of AIT. 30
Table S19. Distribution of patients across AMS levels and statistical comparison between baseline and follow-up at one, two, three and four years of AIT. 31
Table S20. Descriptive statistics of the AMS in the subset of patients who completed five years of AIT. 32
Table S21. Distribution of patients across AMS levels and statistical comparison between baseline and follow-up at one, two, three, four and five years of AIT. 33
Table S22. Descriptive statistics of the RCSMS after one year of AIT. 34
Table S23. Distribution of patients across RCSMS levels and statistical comparison between baseline and follow-up at one year of AIT. 35
Table S24. Descriptive statistics of the RCSMS in the subset of patients who completed two years of AIT. 36
Table S25. Distribution of patients across RCSMS levels and statistical comparison between baseline and follow-up at one and two years of AIT. 37
Table S26. Descriptive statistics of the RCSMS in the subset of patients who completed three years of AIT. 38
Table S27. Distribution of patients across RCSMS levels and statistical comparison between baseline and follow-up at one, two and three years of AIT. 39
Table S28. Descriptive statistics of the RCSMS in the subset of patients who completed four years of AIT. 40
Table S29. Distribution of patients across RCSMS levels and statistical comparison between baseline and follow-up at one, two, three and four years of AIT. 41
Table S30. Descriptive statistics of the RCSMS in the subset of patients who completed five years of AIT. 42
Table S31. Distribution of patients across RCSMS levels and statistical comparison between baseline and follow-up at one, two, three, four and five years of AIT. 43
Table S32. Descriptive statistics of the ACSMS after one year of AIT. 44
Table S33. Distribution of patients across ACSMS levels and statistical comparison between baseline and follow-up at one year of AIT. 45
Table S34. Descriptive statistics of the ACSMS in the subset of patients who completed two years of AIT. 46
Table S35. Distribution of patients across ACSMS levels and statistical comparison between baseline and follow-up at one and two years of AIT. 47
Table S36. Descriptive statistics of the ACSMS in the subset of patients who completed three years of AIT. 48
Table S37. Distribution of patients across ACSMS levels and statistical comparison between baseline and follow-up at one, two and three years of AIT. 49
Table S38. Descriptive statistics of the ACSMS in the subset of patients who completed four years of AIT. 50

Table S39. Distribution of patients across ACSMS levels and statistical comparison between baseline and follow-up at one, two, three and four years of AIT. 51
Table S40. Descriptive statistics of the ACSMS in the subset of patients who completed five years of AIT. 52
Table S41. Distribution of patients across ACSMS levels and statistical comparison between baseline and follow-up at one, two, three, four and five years of AIT. 53

Table S1. Longitudinal adjusted outcomes for RSS and RMS, estimated using mixed models for repeated measures (MMRM).

Time	RSS				RMS			
	**LS-mean**	**SE**	**95% CI**	**% Reduction *versus* T0**	**LS-mean**	**SE**	**95% CI**	**% Reduction *versus* T0**
T0	1.86	0.03	1.79 – 1.92	-	2.11	0.04	2.02 – 2.19	-
T1	0.91	0.03	0.84 – 0.97	51.3	1.35	0.04	1.27 – 1.44	35.8
T2	0.60	0.04	0.53 – 0.68	67.5	1.09	0.05	0.98 – 1.19	48.5
T3	0.44	0.04	0.36 – 0.53	76.1	0.91	0.06	0.79 – 1.03	56.9
T4	0.38	0.05	0.28 – 0.48	79.5	0.70	0.07	0.56 – 0.85	66.7
T5	0.43	0.08	0.27 – 0.59	77.1	0.81	0.12	0.57 – 1.05	61.6

LS-mean: least-squares mean; SE: standard error; CI: confidence interval. RSS: Rhinoconjunctivitis Symptom Score; RMS: Rhinoconjunctivitis Medication Score. LS-means were estimated using mixed models for repeated measures (MMRM) including time (categorical) as a fixed effect. For rhinoconjunctivitis outcomes, models additionally included age, gender and asthma status as fixed effects. Percent reduction represents the relative change in LS-mean values compared with baseline (T0).

Table S2. Longitudinal adjusted outcomes for ASS and AMS, estimated using mixed models for repeated measures (MMRM).

Time	ASS				AMS			
	**LS-mean**	**SE**	**95% CI**	**% Reduction *versus* T0**	**LS-mean**	**SE**	**95% CI**	**% Reduction *versus* T0**
T0	1.25	0.04	1.17 – 1.34	-	1.49	0.06	1.37 – 1.61	-
T1	0.46	0.04	0.38 – 0.55	63.0	0.89	0.06	0.77 – 1.02	39.9
T2	0.24	0.05	0.14 – 0.34	81.0	0.58	0.07	0.43 – 0.72	61.3
T3	0.15	0.06	0.04 – 0.27	87.9	0.49	0.08	0.33 – 0.65	67.2
T4	0.16	0.07	0.02 – 0.31	87.1	0.32	0.10	0.12 – 0.52	78.5
T5	0.19	0.11	- .02 – 0.39	85.3	0.28	0.15	-0.01 – 0.57	81.1

LS-mean: least-squares mean; SE: standard error; CI: confidence interval. ASS: Asthma Symptom Score; AMS: Asthma Medication Score. LS-means were estimated using mixed models for repeated measures (MMRM) including time (categorical) as a fixed effect. Asthma outcomes were analysed in the asthma subgroup and included age and gender as fixed effects. Percent reduction represents the relative change in LS-mean values compared with baseline (T0).

Table S3. Evolution of Symptom Scores across the study period.

	T0	T1	T2	T3	T4	T5
			**RSS**			
**Mean (SD)**	1.8 (0.5)	0.9 (0.5)	0.6 (0.5)	0.4 (0.4)	0.3 (0.4)	0.4 (0.4)
**Median (Q1, Q3)**	1.8 (1.5, 2.2)	0.8 (0.7, 1.2)	0.5 (0.3, 0.8)	0.3 (0.2, 0.5)	0.2 (0.2, 0.5)	0.3 (0.2, 0.5)
**n**	208	208	126	104	65	24
**%Reduction *vs.* T0 $_{Median}$**	-	54.5	72.7	81.8	90.9	81.8
***p* (Wilcoxon)**	-	<0.0001[a]	-	-	-	-
***p* (Friedman)**	-	-	<0.0001[b]	<0.0001[c]	<0.0001[d]	<0.0001[e]
**Effect size**	-	0.84[a]	0.85[b]	0.82[c]	0.76[d]	0.69[e]
**Interpretation effect size**	-	Large[a]	Large[b]	Large[c]	Large[d]	Large[e]
			**ASS**			
**Mean (SD)**	1.3 (0.6)	0.5 (0.5)	0.2 (0.3)	0.2 (0.3)	0.2 (0.3)	0.3 (0.4)
**Median (Q1, Q3)**	1.3 (0.8, 1.8)	0.3 (0.0, 0.8)	0.0 (0.0, 0.5)	0.0 (0.0, 0.3)	0.0 (0.0, 0.3)	0.0 (0.0, 0.4)
**n**	124	124	72	61	36	17
**%Reduction *vs.* T0 $_{Median}$**	-	80.0	100.0	100.0	100.0	100.0
***p* (Wilcoxon)**	-	<0.0001[a]	-	-	-	-
***p* (Friedman)**	-	-	<0.0001[b]	<0.0001[c]	<0.0001[d]	<0.0001[e]
**Effect size**	-	0.81[a]	0.72[b]	0.72[c]	0.71[d]	0.58[e]
**Interpretation effect size**	-	Large[a]	Large[b]	Large[c]	Large[d]	Large[e]

[a]Comparison performed in patients with available data at baseline and year 1. [b]Comparison performed in patients with available data at baseline and year 2. [c]Comparison performed in patients with available data at baseline and year 3. [d]Comparison performed in patients with available data at baseline and year 4. [e]Comparison performed in patients with available data at baseline and year 5. Effect sizes correspond to the Wilcoxon signed-rank test (Wilcoxon r; T0-T1) or the Friedman test (Kendall's W; T0-T5) within each respective subgroup. RSS: Rhinoconjunctivitis Symptom Score. ASS: Asthma Symptom Score. SD: Standard Deviation. Q1: First quartile. Q3: Third quartile.

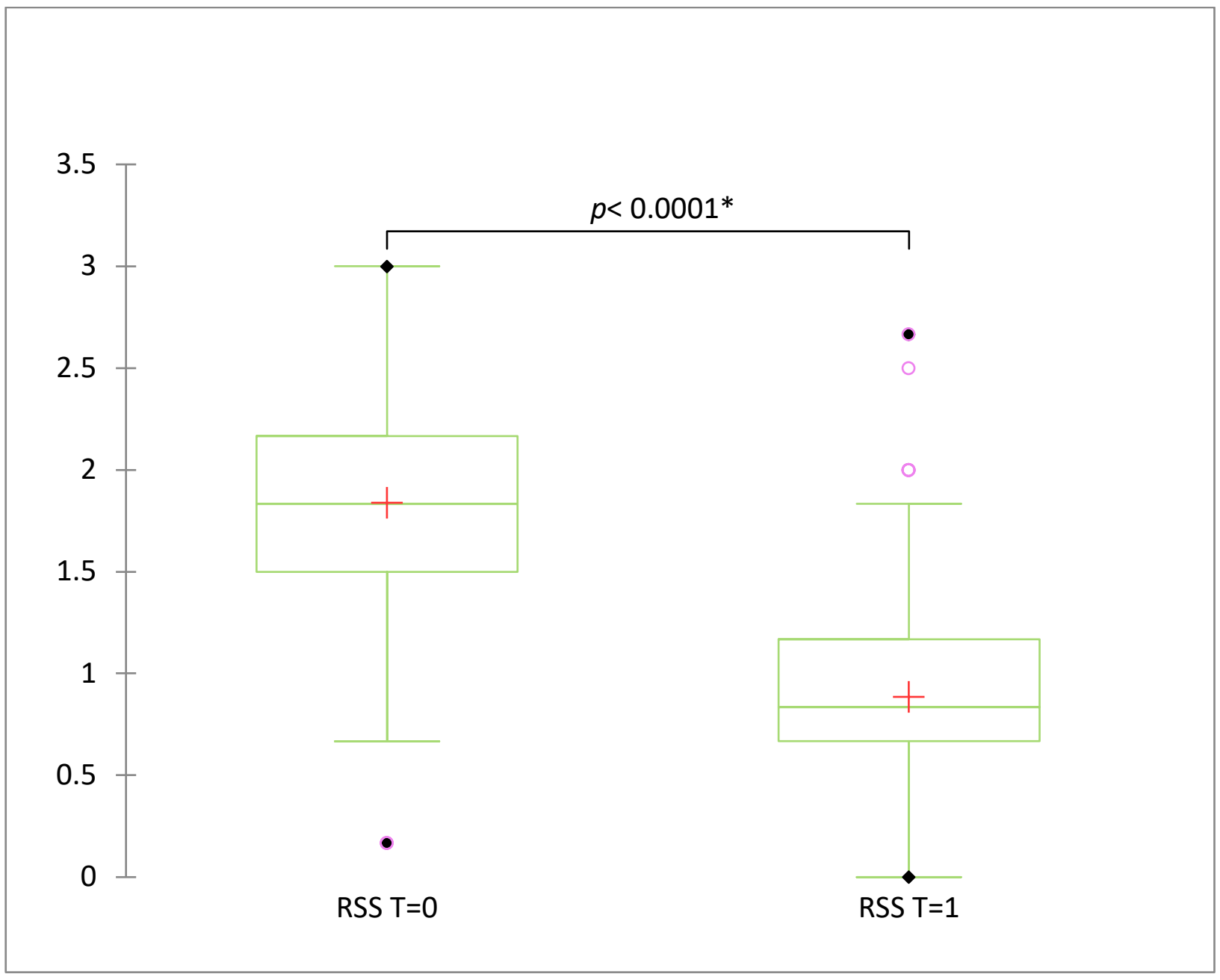

Figure S1. Box plot of the RSS at baseline and after one year of immunotherapy.
Boxes represent the interquartile range (IQR), horizontal lines indicate medians, and whiskers denote minimum and maximum values. Red crosses represent mean values. Pink symbols represent atypical values. *Reaching statistical significance at level α≤0.05. RSS: Rhinoconjunctivitis Symptom Score.

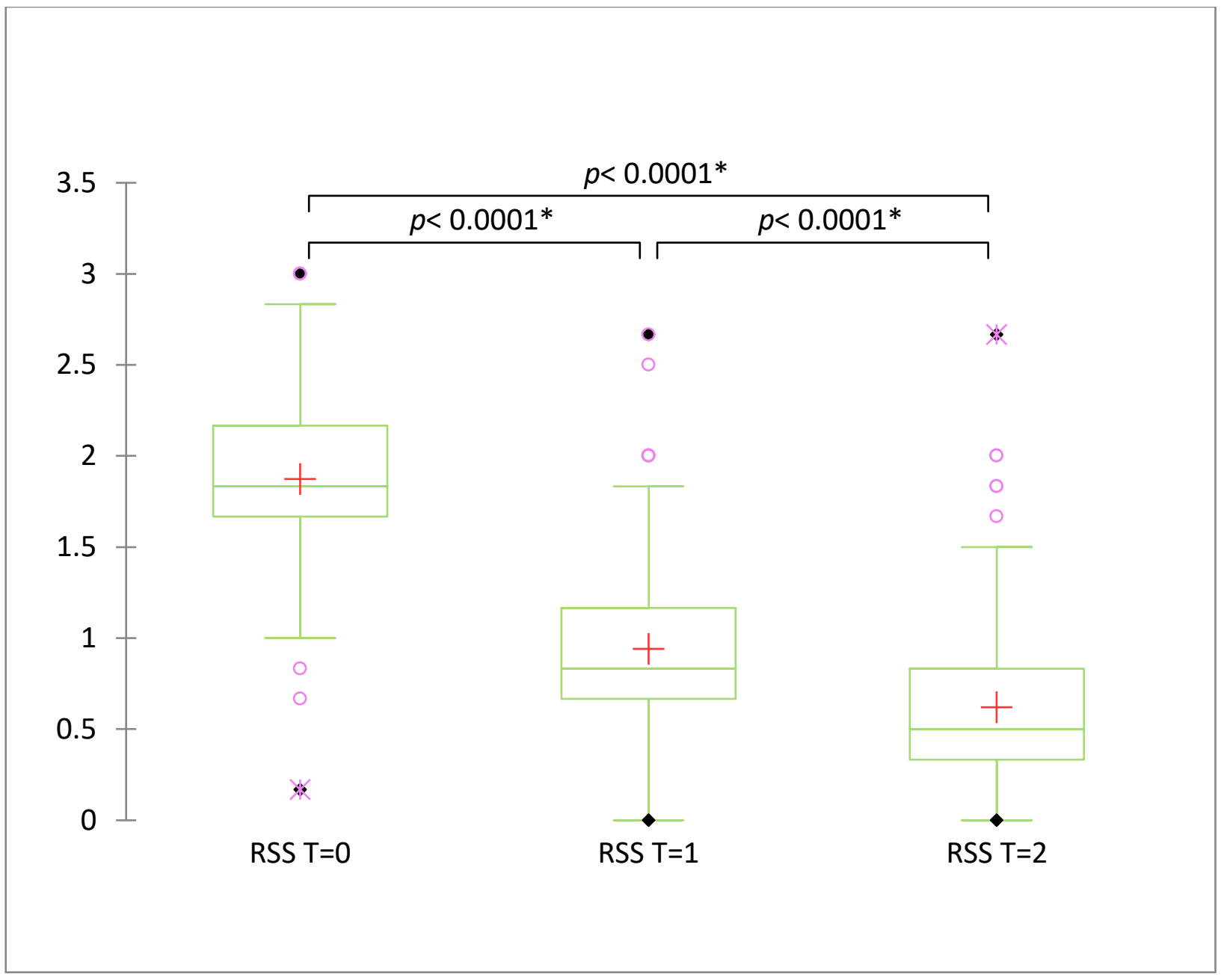

Figure S2. Box plot of the RSS at baseline, after one year, and after two years of immunotherapy in patients with complete two-year AIT data.
Boxes represent the interquartile range (IQR), horizontal lines indicate medians, and whiskers denote minimum and maximum values. Red crosses represent mean values. Pink symbols represent atypical values. *Reaching statistical significance at level α≤0.05. RSS: Rhinoconjunctivitis Symptom Score.

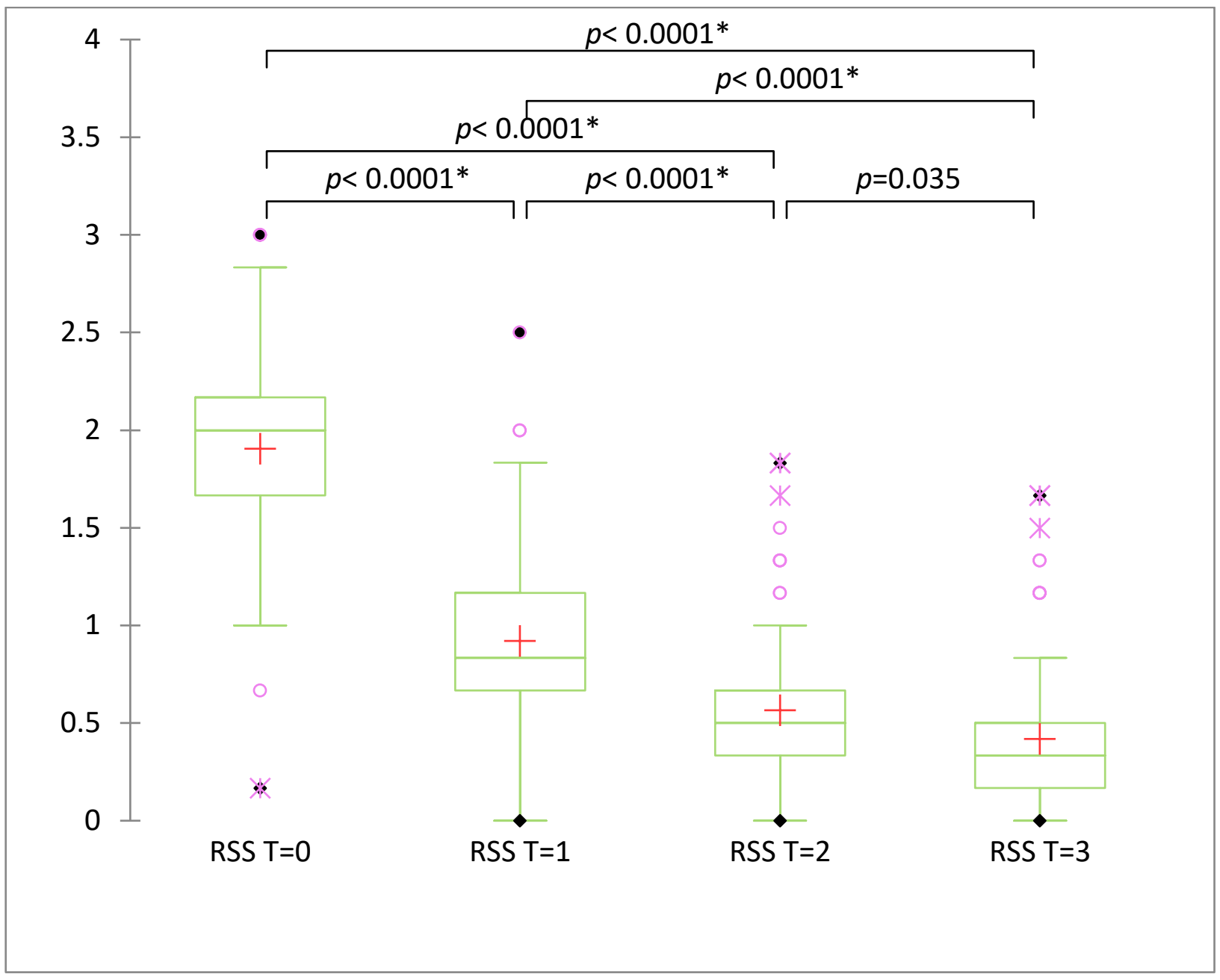

Figure S3. Box plot of the RSS at baseline, after one year, two years, and three years of immunotherapy in patients with complete three-year AIT data.

Boxes represent the interquartile range (IQR), horizontal lines indicate medians, and whiskers denote minimum and maximum values. Red crosses represent mean values. Pink symbols represent atypical values. *Reaching statistical significance at level α≤0.05. RSS: Rhinoconjunctivitis Symptom Score.

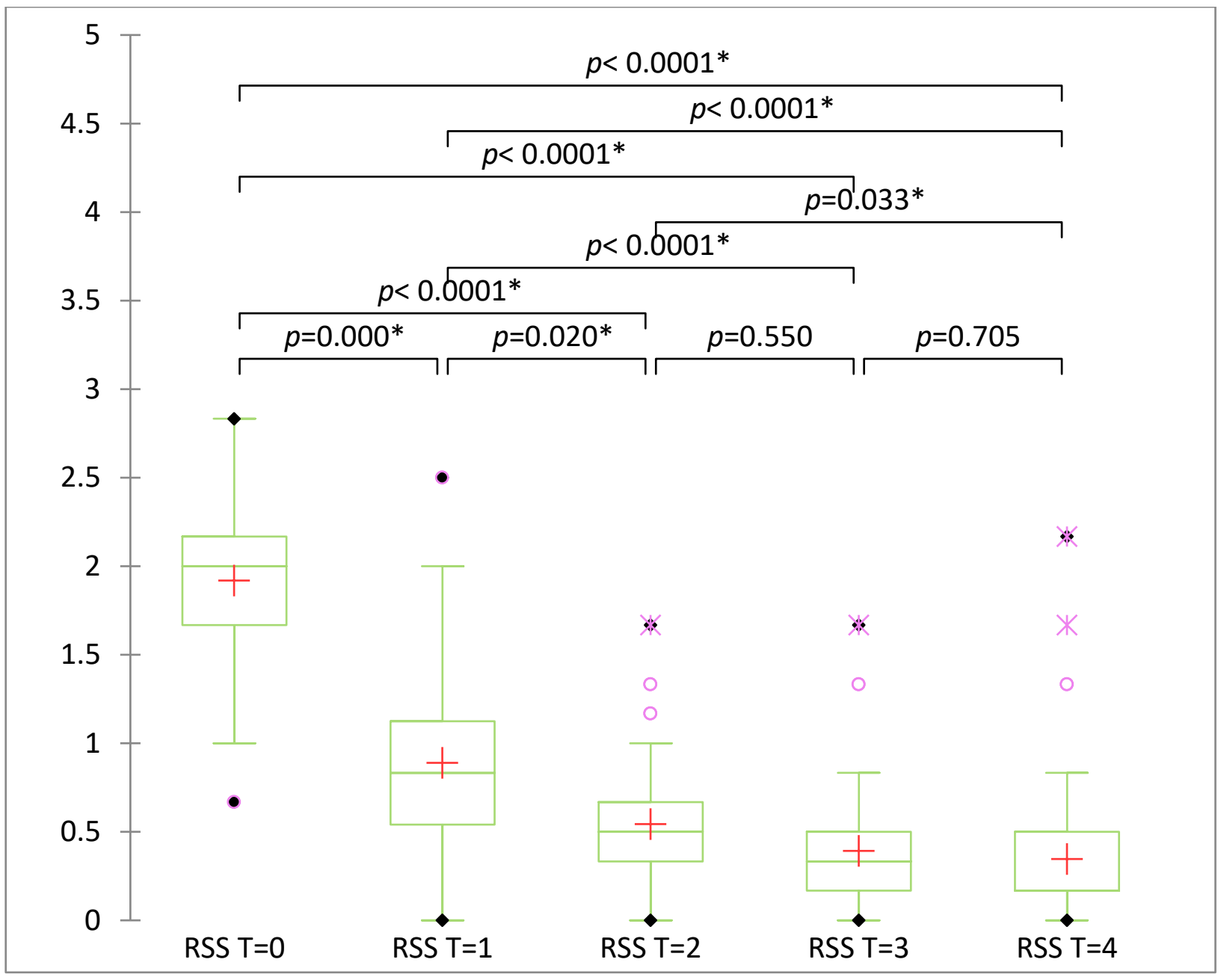

Figure S4. Box plot of the RSS at baseline and after up to four years of immunotherapy, in patients with complete four-year AIT data.

Boxes represent the interquartile range (IQR), horizontal lines indicate medians, and whiskers denote minimum and maximum values. Red crosses represent mean values. Pink symbols represent atypical values. *Reaching statistical significance at level α≤0.05. RSS: Rhinoconjunctivitis Symptom Score.

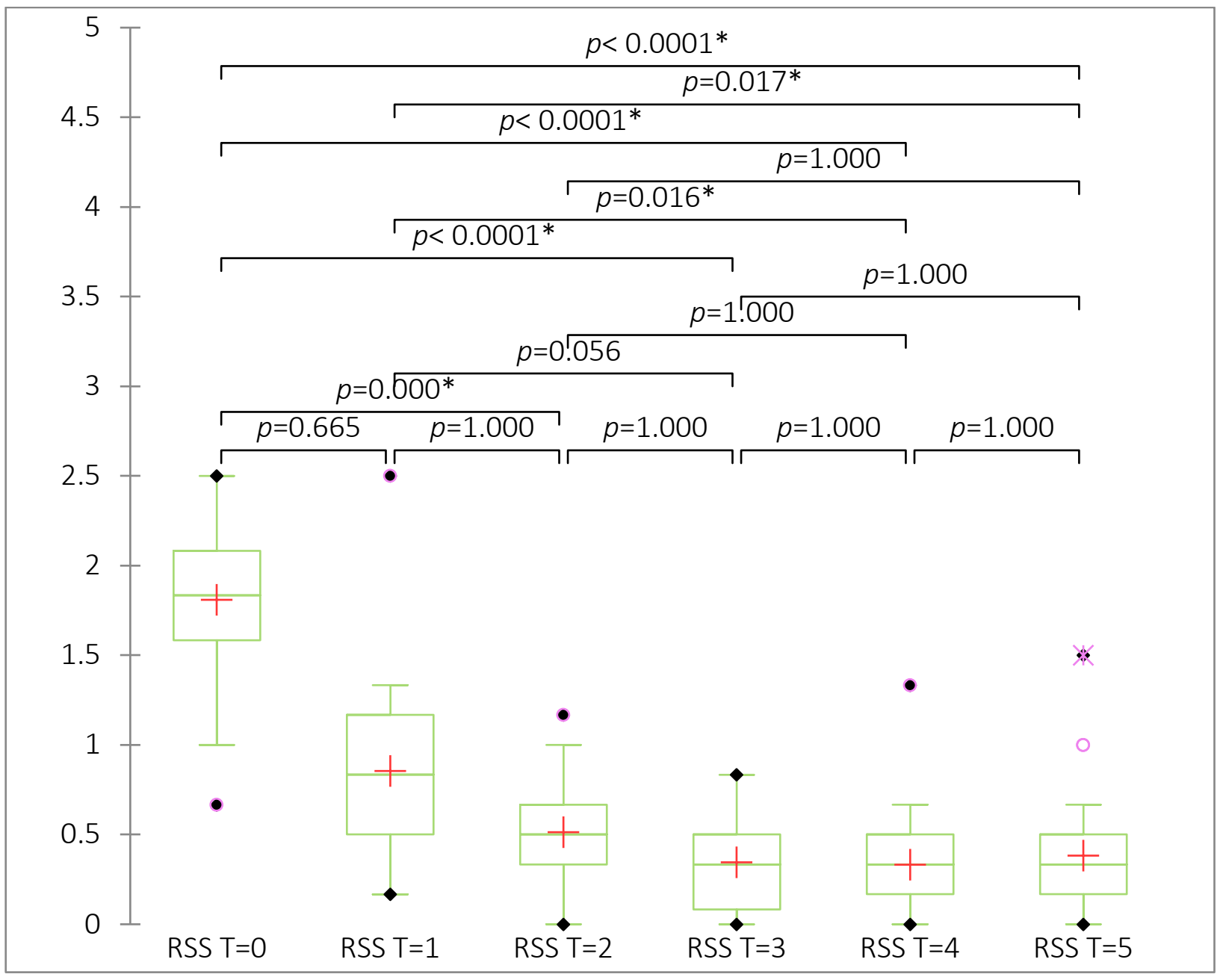

Figure S5. Box plot of the RSS from baseline to five years of immunotherapy, in patients with complete five-year AIT data.

Boxes represent the interquartile range (IQR), horizontal lines indicate medians, and whiskers denote minimum and maximum values. Red crosses represent mean values. Pink symbols represent atypical values. *Reaching statistical significance at level α≤0.05. RSS: Rhinoconjunctivitis Symptom Score.

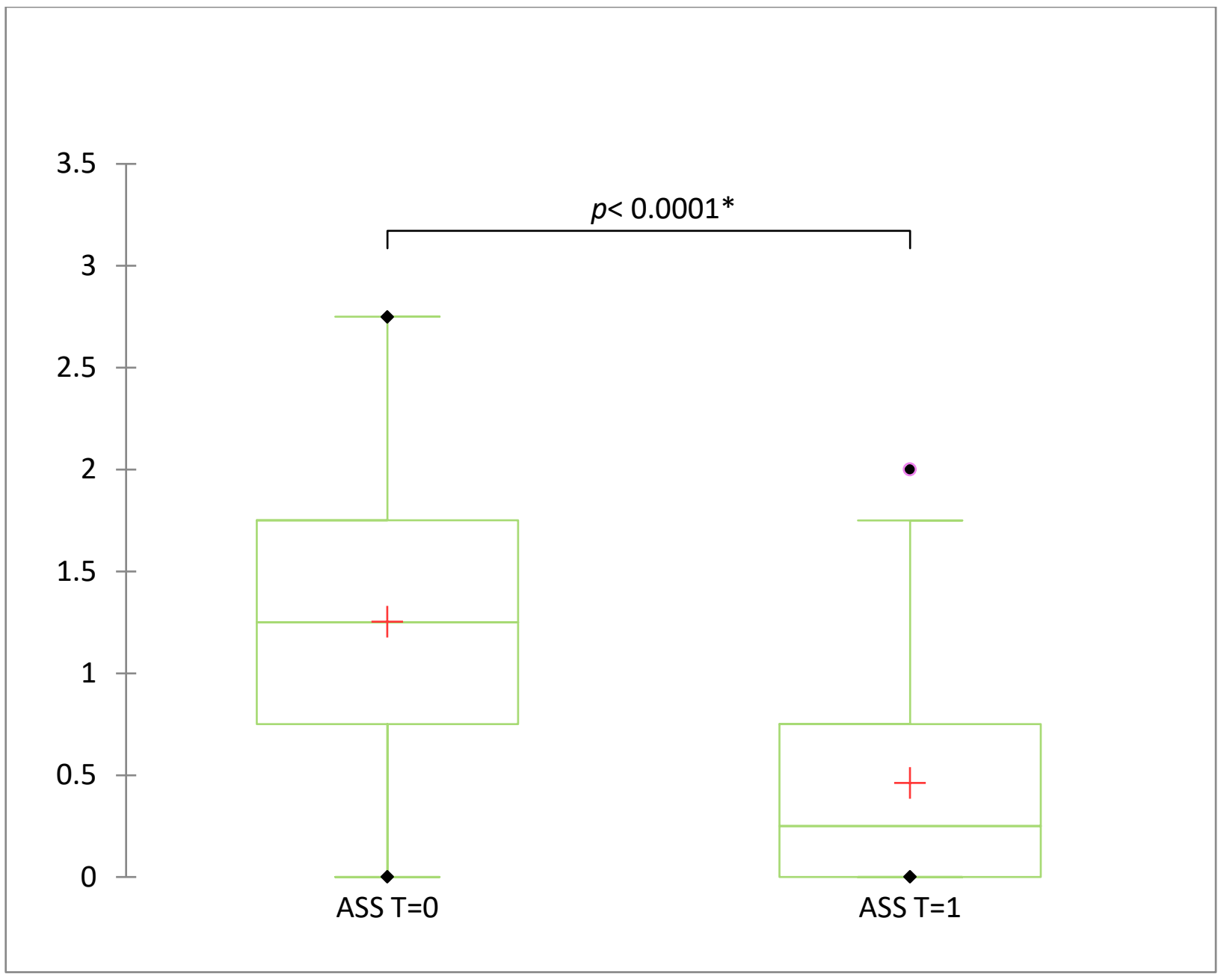

Figure S6. Box plot of the ASS at baseline and after one year of immunotherapy.

Boxes represent the interquartile range (IQR), horizontal lines indicate medians, and whiskers denote minimum and maximum values. Red crosses represent mean values. Pink symbols represent atypical values. *Reaching statistical significance at level α≤0.05. ASS: Asthma Symptom Score.

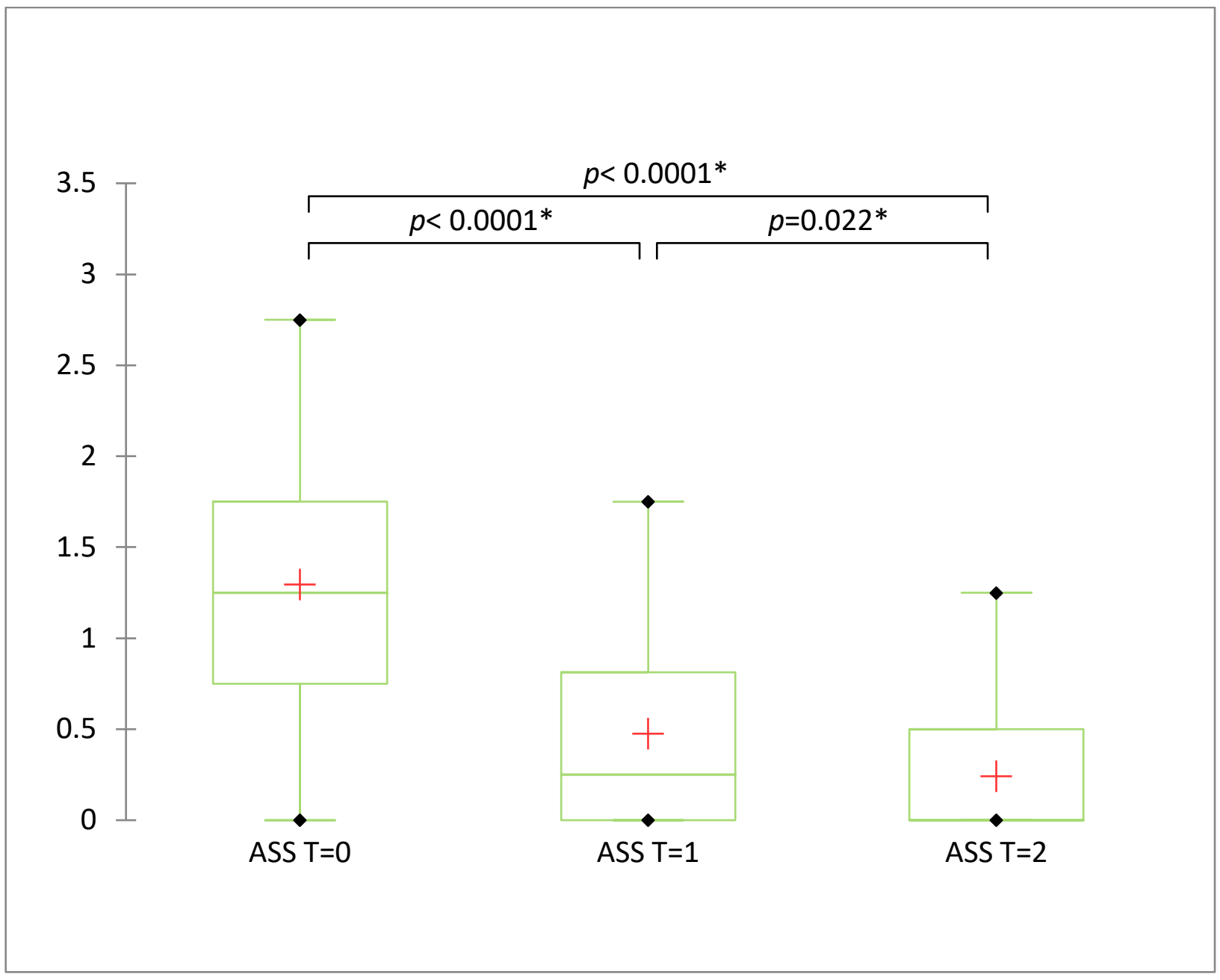

Figure S7. Box plot of the ASS at baseline, and after one and two years of immunotherapy in patients with complete two-year AIT data.
Boxes represent the interquartile range (IQR), horizontal lines indicate medians, and whiskers denote minimum and maximum values. Red crosses represent mean values. Pink symbols represent atypical values. *Reaching statistical significance at level α≤0.05. ASS: Asthma Symptom Score.

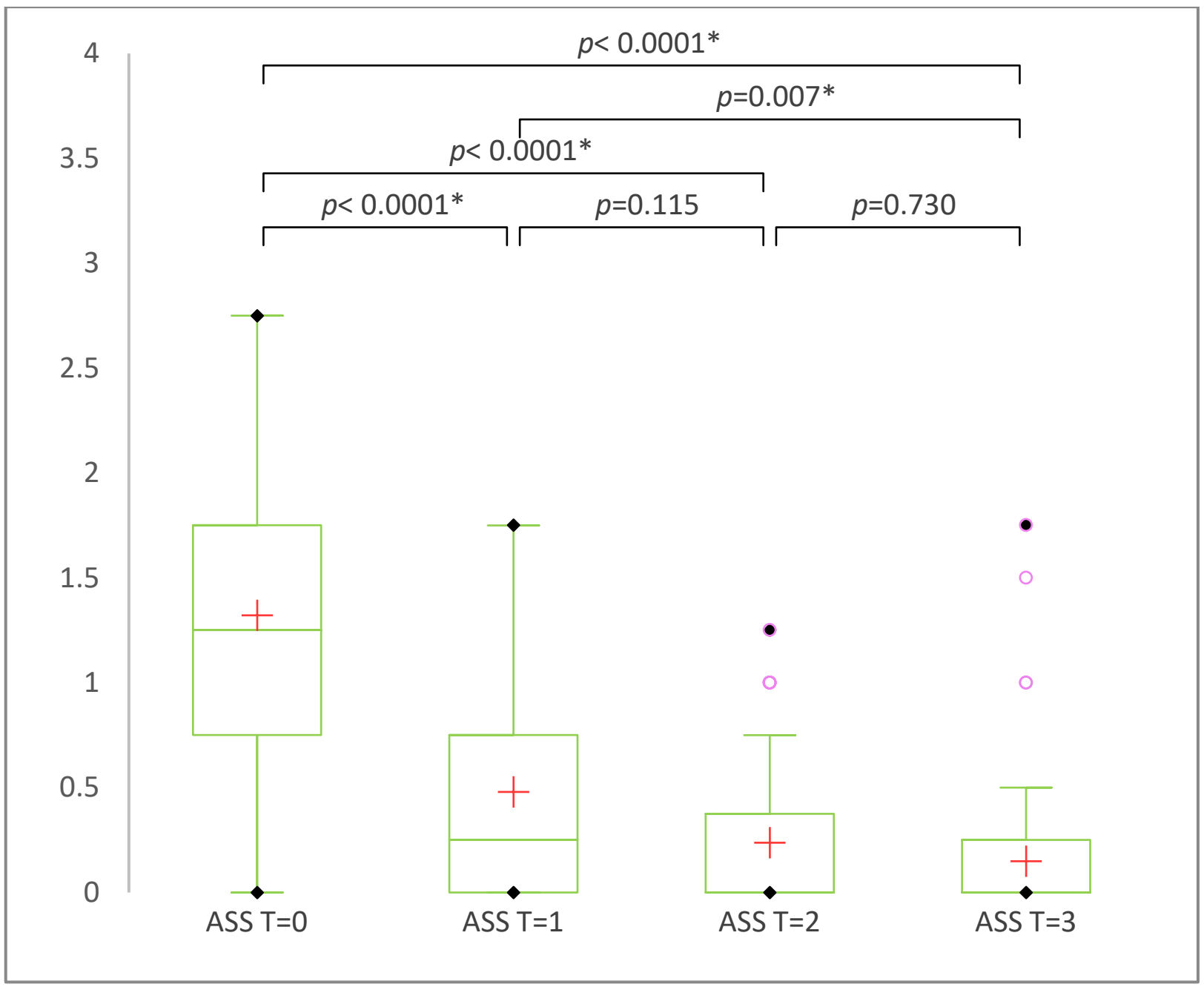

Figure S8. Box plot of the ASS at baseline, and after one, two, and three years of immunotherapy in patients with complete three-year AIT data.
Boxes represent the interquartile range (IQR), horizontal lines indicate medians, and whiskers denote minimum and maximum values. Red crosses represent mean values. Pink symbols represent atypical values. *Reaching statistical significance at level α≤0.05. ASS: Asthma Symptom Score.

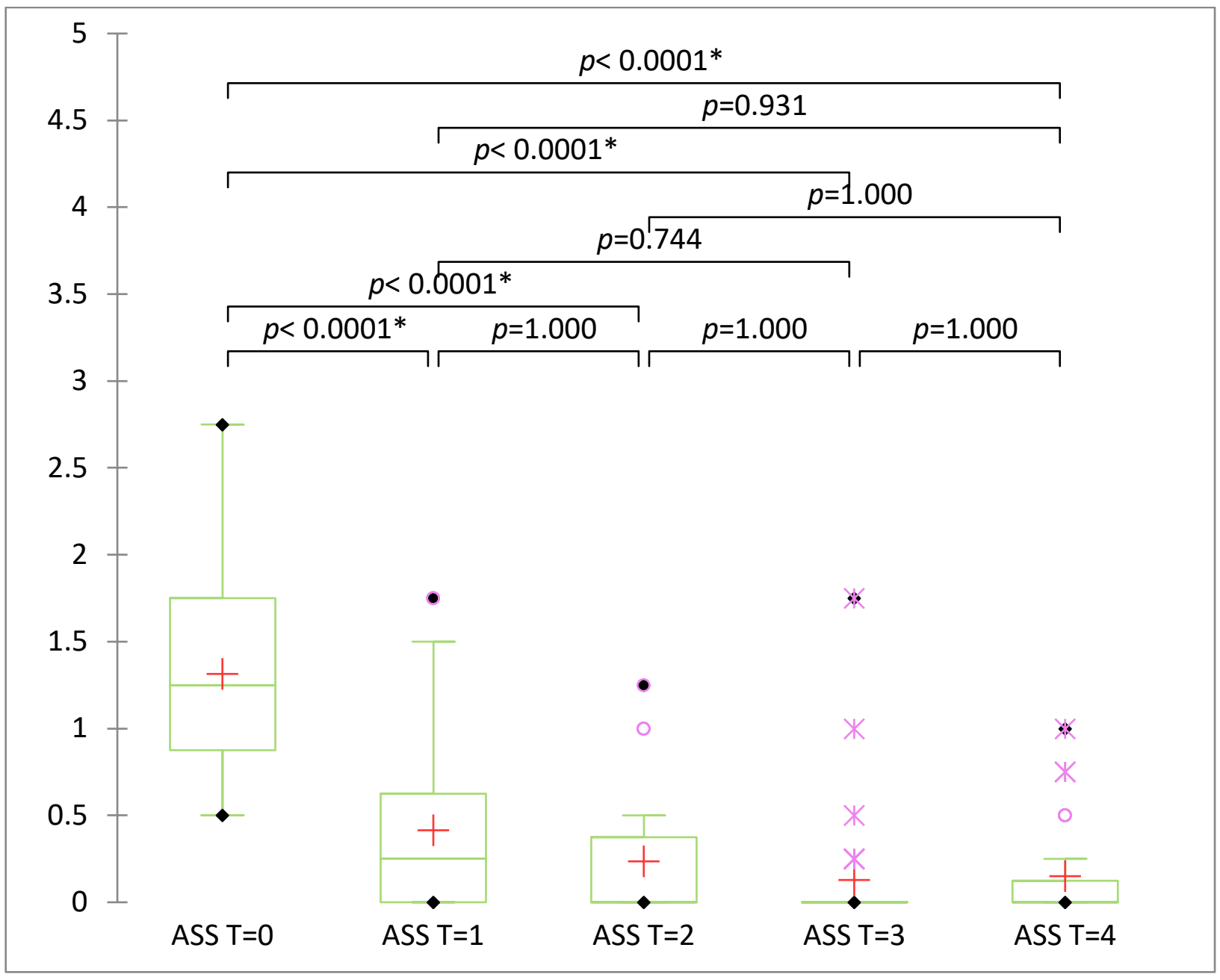

Figure S9. Box plot of the ASS at baseline and after one to four years of immunotherapy in patients with complete four-year AIT data.

Boxes represent the interquartile range (IQR), horizontal lines indicate medians, and whiskers denote minimum and maximum values. Red crosses represent mean values. Pink symbols represent atypical values. *Reaching statistical significance at level α≤0.05. ASS: Asthma Symptom Score.

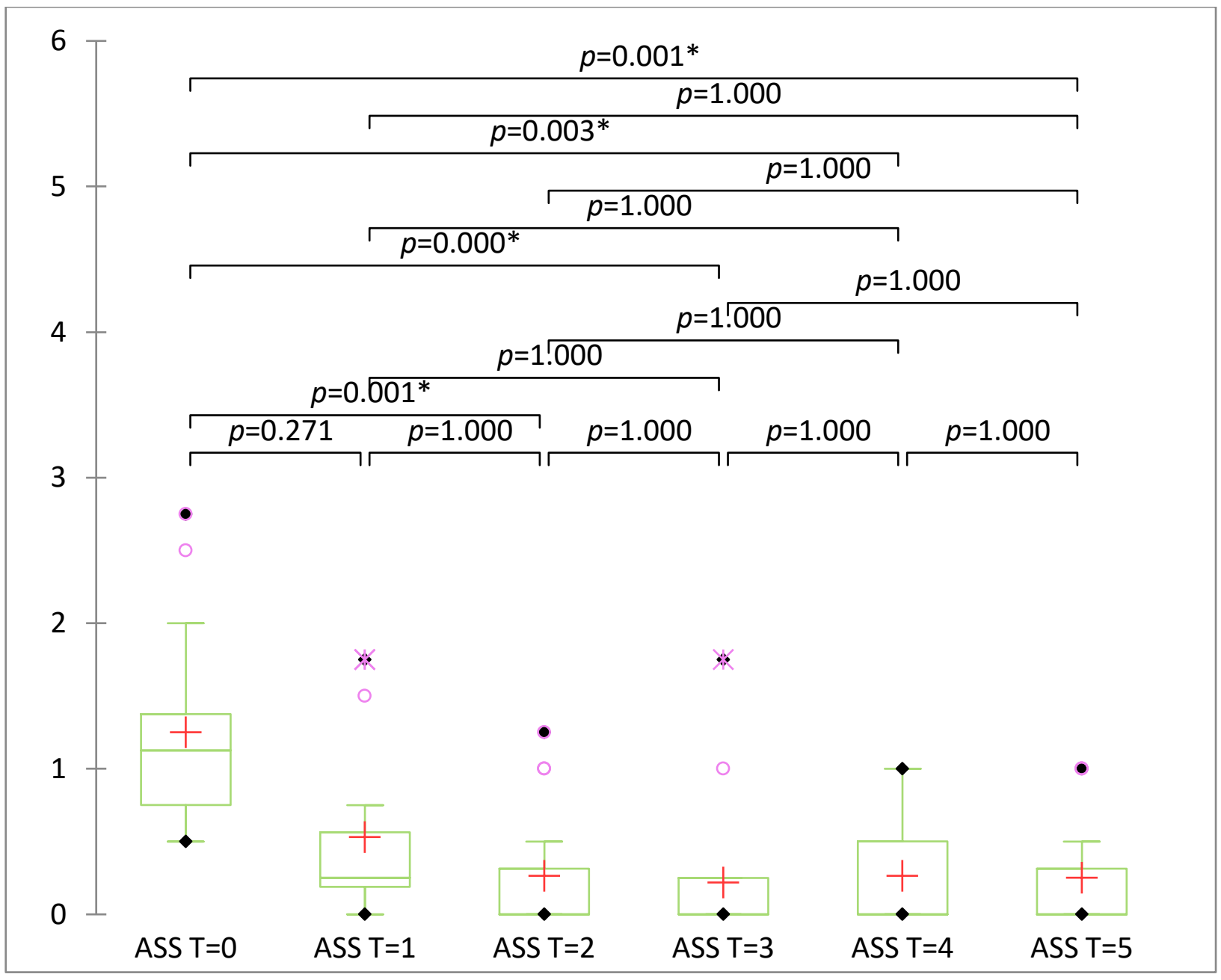

Figure S10. Box plot of the ASS from baseline to five years of immunotherapy, in patients with complete five-year AIT data.

Boxes represent the interquartile range (IQR), horizontal lines indicate medians, and whiskers denote minimum and maximum values. Red crosses represent mean values. Pink symbols represent atypical values. *Reaching statistical significance at level α≤0.05. ASS: Asthma Symptom Score.

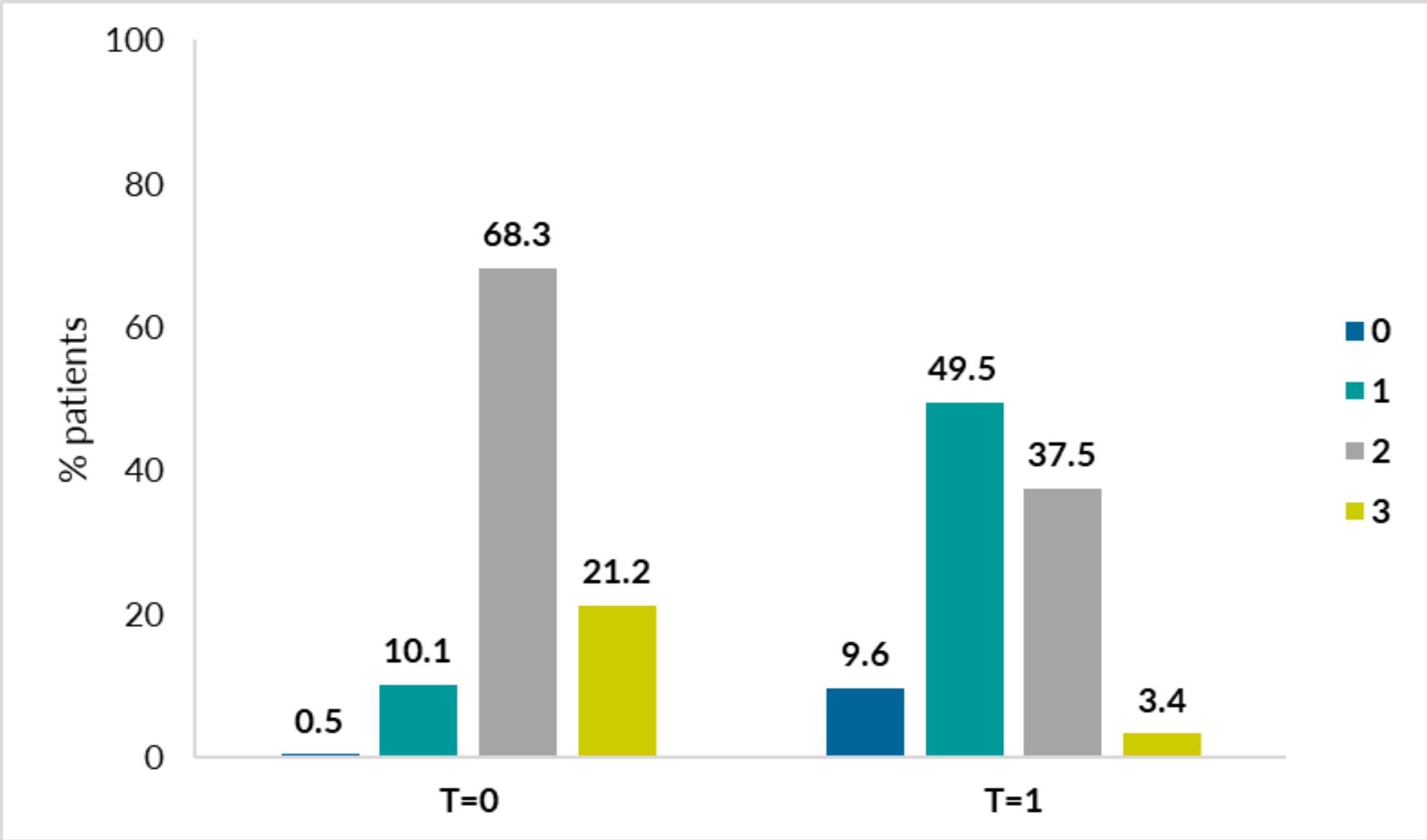

Figure S11. Distribution of patients according to RMS at baseline and after one year of treatment.

Table S4. Distribution of patients across RMS levels and statistical comparison between baseline and one year of AIT.

		RMS	
	**Score**	**T=0**	**T=1**
	**0**	1	20
	**>0**	207	188
**χ^2**	-	-	18.104
p	-	-	<0.001
**ϕ**	-	-	0.209
**Effect Size**	-	-	Small
	**0 - 1**	22	123
	**>1**	186	85
**χ^2**	-	-	107.99
p	-	-	<0.001
**ϕ**	-	-	0.51
**Effect Size**	-	-	Large
	**<3**	164	201
	**3**	44	7
**χ^2**	-	-	30.594
p	-	-	<0.001
**ϕ**	-	-	0.271
**Effect Size**	-	-	Small

RMS: Rhinoconjunctivitis Medication Score. χ^2: chi-square test; ϕ: Phi coefficient.
Effect sizes were as small (ϕ = 0.10), moderate (ϕ = 0.30) or large (ϕ ≥ 0.50). All comparisons were performed using Pearson's χ^2 test.

Table S5. Descriptive statistics of the RMS in the subset of patients who completed two years of AIT.

	RMS		
	**T=0**	**T=1**	**T=2**
**Mean**	2.20	1.43	1.11
**95% lower CI**	2.10	1.31	1.01
**95% upper CI**	2.30	1.55	1.21
**SD**	0.58	0.67	0.58
**Median**	2	1	1
**Q1**	2	1	1
**Q3**	3	2	1
**Maximum**	3	3	3
**Minimum**	0	0	0
**n**	126	126	126

RMS: Rhinoconjunctivitis Medication Score. CI: Confidence Interval. SD: Standard Deviation. Q1: First quartile. Q3: Third quartile.

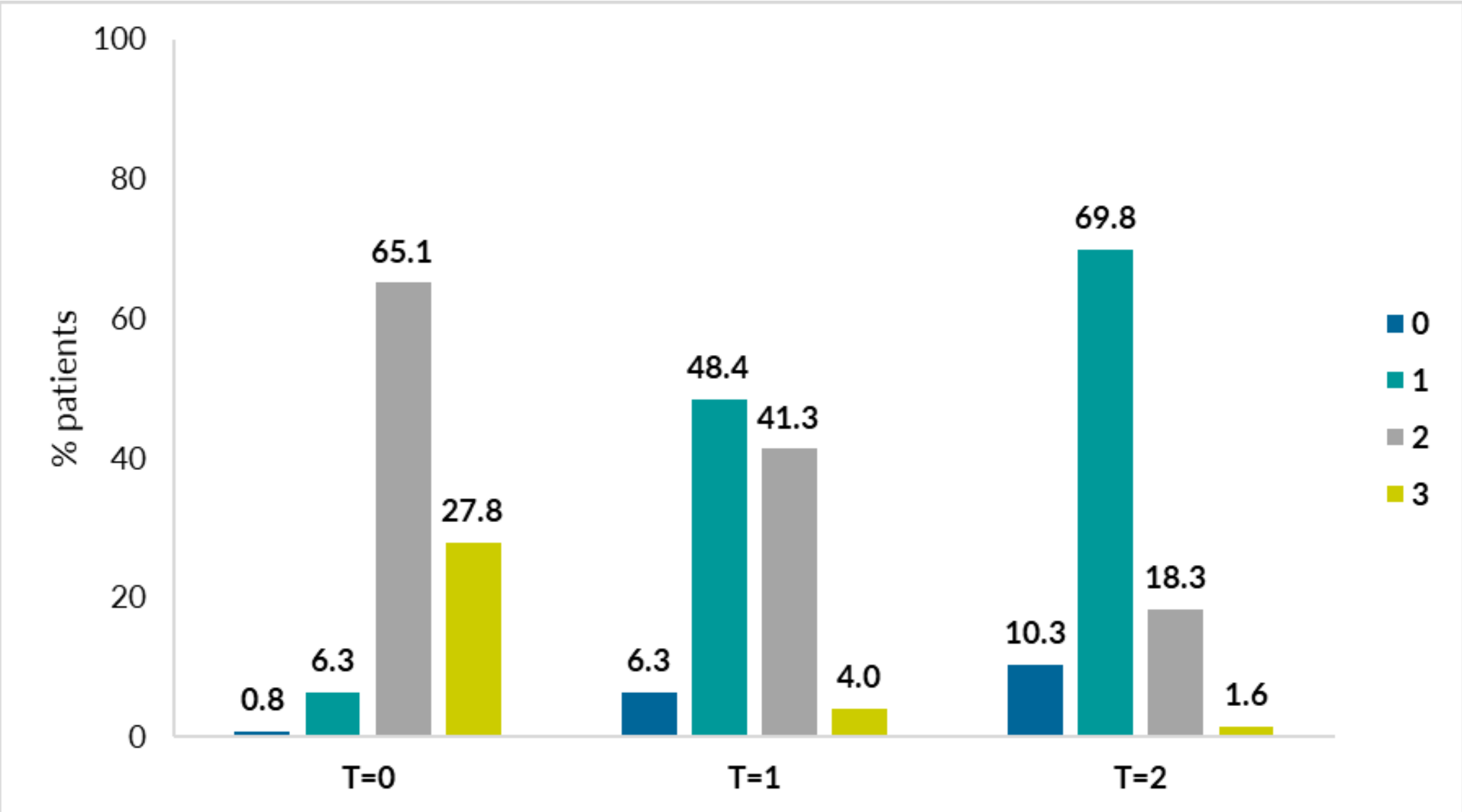

Figure S12. Distribution of patients according to RMS at baseline and after one and years of treatment.

Table S6. Distribution of patients across RMS levels and statistical comparison between baseline and follow-up at one and two years of AIT.

	RMS			
	**Score**	**T=0**	**T=1**	**T=2**
	**0**	1	8	13
	**>0**	125	118	113
**χ^2**	-	-	5.646	10.891
p	-	-	0.017	<0.001
**ϕ**	-	-	0.15	0.208
**Effect Size**	-	-	Small	Small
	**0 - 1**	9	69	101
	**>1**	117	57	25
**χ^2**	-	-	66.84	136.55
p	-	-	<0.001	<0.001
**ϕ**	-	-	0.515	0.736
**Effect Size**	-	-	Large	Large
	**<3**	91	121	124
	**3**	35	5	2
**χ^2**	-	-	26.745	34.498
p	-	-	<0.001	<0.001
**ϕ**	-	-	0.326	0.37
**Effect Size**	-	-	Moderate	Moderate

RMS: Rhinoconjunctivitis Medication Score. χ^2: chi-square test; ϕ: Phi coefficient.
Effect sizes were as small (ϕ = 0.10), moderate (ϕ = 0.30) or large (ϕ ≥ 0.50). All comparisons were performed using Pearson's χ^2 test.

Table S7. Descriptive statistics of the RMS in the subset of patients who completed three years of AIT.

	RMS			
	T=0	T=1	T=2	T=3
**Mean**	2.24	1.39	1.06	0.89
**95% lower CI**	2.12	1.27	0.95	0.78
**95% upper CI**	2.36	1.52	1.17	1.01
**SD**	0.60	0.66	0.56	0.59
**Median**	2	1	1	1
**Q1**	2	1	1	1
**Q3**	3	2	1	1
**Maximum**	3	3	3	3
**Minimum**	0	0	0	0
**n**	104	104	101	104

RMS: Rhinoconjunctivitis Medication Score. CI: Confidence Interval. SD: Standard Deviation. Q1: First quartile. Q3: Third quartile.

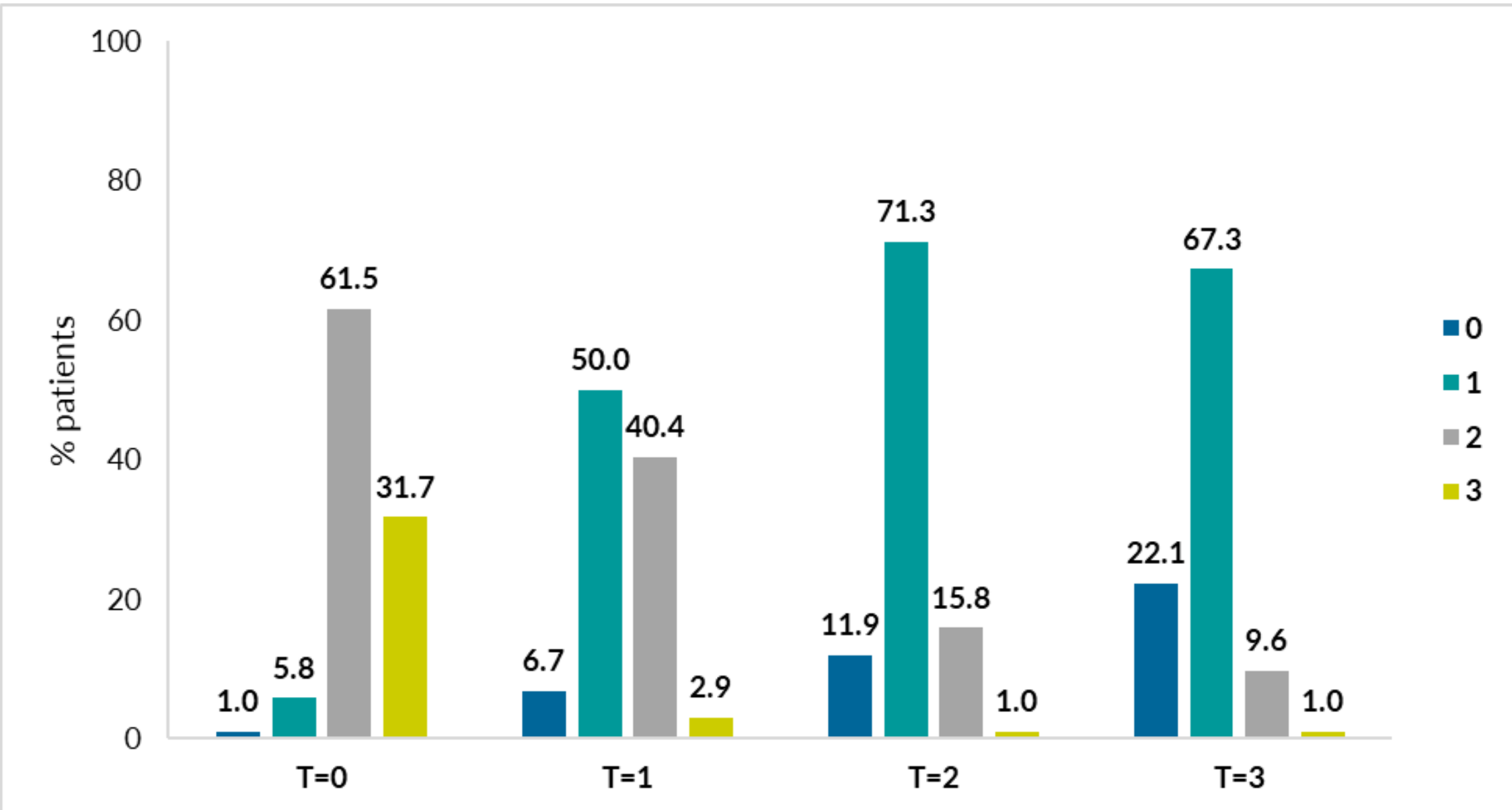

Figure S13. Distribution of patients according to RMS at baseline and after up to three years of treatment in patients with complete three-year AIT data.

Table S8. Distribution of patients across RMS levels and statistical comparison between baseline and follow-up at one, two and three years of AIT.

		RMS			
	**Score**	**T=0**	**T=1**	**T=2**	**T=3**
	**0**	1	7	12	23
	**>0**	103	97	89	81
**χ^2**	-	-	4.68	10.287	22.797
p	-	-	0.031	0.001	<0.001
**ϕ**	-	-	0.15	0.224	0.331
**Effect Size**	-	-	Small	Small	Moderate
	**0 - 1**	-	59	84	93
	**>1**	-	45	17	11
**χ^2**	-	-	60.01	121.28	142.44
p	-	-	<0.001	<0.001	<0.001
**ϕ**	-	-	0.537	0.769	0.828
**Effect Size**	-	-	Large	Large	Large
	**<3**	71	101	100	103
	**3**	33	3	1	1
**χ^2**	-	-	30.233	34.999	36.003
p	-	-	<0.001	<0.001	<0.001
**ϕ**	-	-	0.381	0.413	0.416
**Effect Size**	-	-	Moderate	Moderate	Moderate

RMS: Rhinoconjunctivitis Medication Score. χ^2: chi-square test; ϕ: Phi coefficient.
Effect sizes were as small (ϕ = 0.10), moderate (ϕ = 0.30) or large (ϕ ≥ 0.50). All comparisons were performed using Pearson's χ^2 test.

Table S9. Descriptive statistics of the RMS in the subset of patients who completed four years of AIT.

	RMS				
	T=0	T=1	T=2	T=3	T=4
**Mean**	2.29	1.38	1.05	0.86	0.68
**95% lower CI**	2.14	1.21	0.89	0.72	0.52
**95% upper CI**	2.45	1.56	1.20	1.00	0.84
**SD**	0.63	0.70	0.61	0.56	0.64
**Median**	2	1	1	1	1
**Q1**	2	1	1	1	0
**Q3**	3	2	1	1	1
**Maximum**	3	3	3	3	3
**Minimum**	1	0	0	0	0
**n**	65	65	63	64	65

RMS: Rhinoconjunctivitis Medication Score. CI: Confidence Interval. SD: Standard Deviation. Q1: First quartile. Q3: Third quartile.

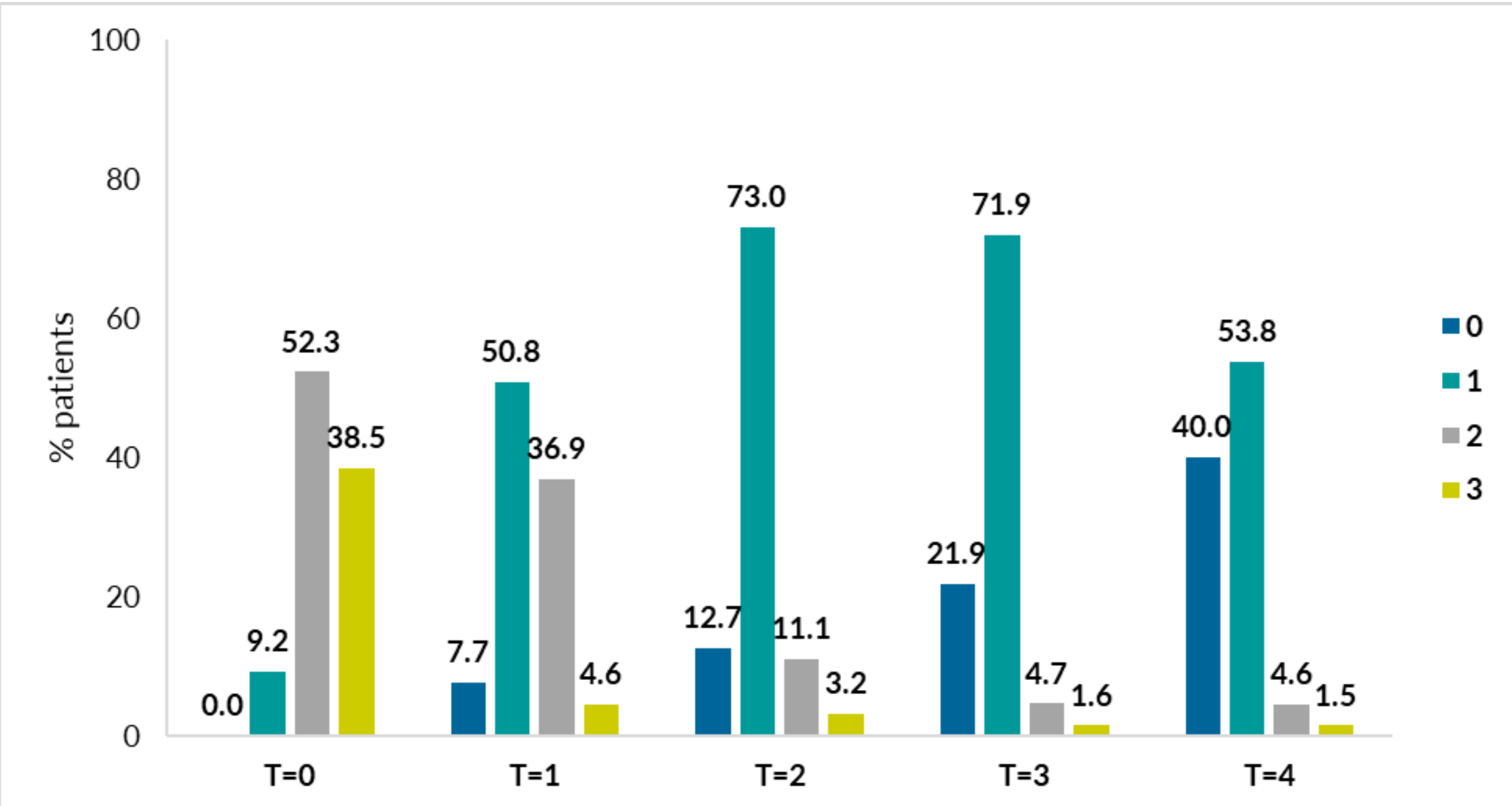

Figure S14. Distribution of patients according to RMS at baseline and after up to four years of treatment in patients with complete four-year AIT data.

Table S10. Distribution of patients across RMS levels and statistical comparison between baseline and follow-up at one, two, three and four years of AIT.

		RMS				
	**Score**	**T=0**	**T=1**	**T=2**	**T=3**	**T=4**
	**0**	0	5	8	14	26
	**>0**	65	60	55	50	39
**χ^2**	-	-	5.2	8.804	15.95	32.5
p	-	-	0.023	0.003	<0.001	<0.001
**ϕ**	-	-	0.2	0.262	0.352	0.5
**Effect Size**	-	-	Small	Small	Moderate	Large
	**0 - 1**	6	38	54	60	61
	**>1**	59	27	9	4	4
**χ^2**	-	-	35.18	75.15	92.20	93.17
p	-	-	<0.001	<0.001	<0.001	<0.001
**ϕ**	-	-	0.52	0.766	0.845	0.847
**Effect Size**	-	-	Large	Large	Large	Large
	**<3**	40	62	61	63	64
	**3**	25	3	2	1	1
**χ^2**	-	-	22.031	23.934	27.284	27.692
p	-	-	<0.001	<0.001	<0.001	<0.001
**ϕ**	-	-	0.412	0.432	0.46	0.462
**Effect Size**	-	-	Moderate	Moderate	Moderate	Moderate

RMS: Rhinoconjunctivitis Medication Score. χ^2: chi-square test; ϕ: Phi coefficient.
Effect sizes were as small (ϕ = 0.10), moderate (ϕ = 0.30) or large (ϕ ≥ 0.50). All comparisons were performed using Pearson's χ^2 test.

Table S11. Descriptive statistics of the RMS in the subset of patients who completed five years of AIT.

	RMS					
	T=0	T=1	T=2	T=3	T=4	T=5
**Mean**	2.33	1.58	1.00	0.78	0.67	0.79
**95% lower CI**	2.01	1.31	0.75	0.56	0.46	0.62
**95% upper CI**	2.65	1.86	1.25	1.01	0.87	0.97
**SD**	0.76	0.65	0.59	0.52	0.48	0.41
**Median**	2.5	1.5	1	1	1	1
**Q1**	2	1	1	0	0	1
**Q3**	3	2	1	1	1	1
**Maximum**	3	3	3	2	1	1
**Minimum**	1	1	0	0	0	0
**n**	24	24	24	23	24	24

RMS: Rhinoconjunctivitis Medication Score. CI: Confidence Interval. SD: Standard Deviation. Q1: First quartile. Q3: Third quartile.

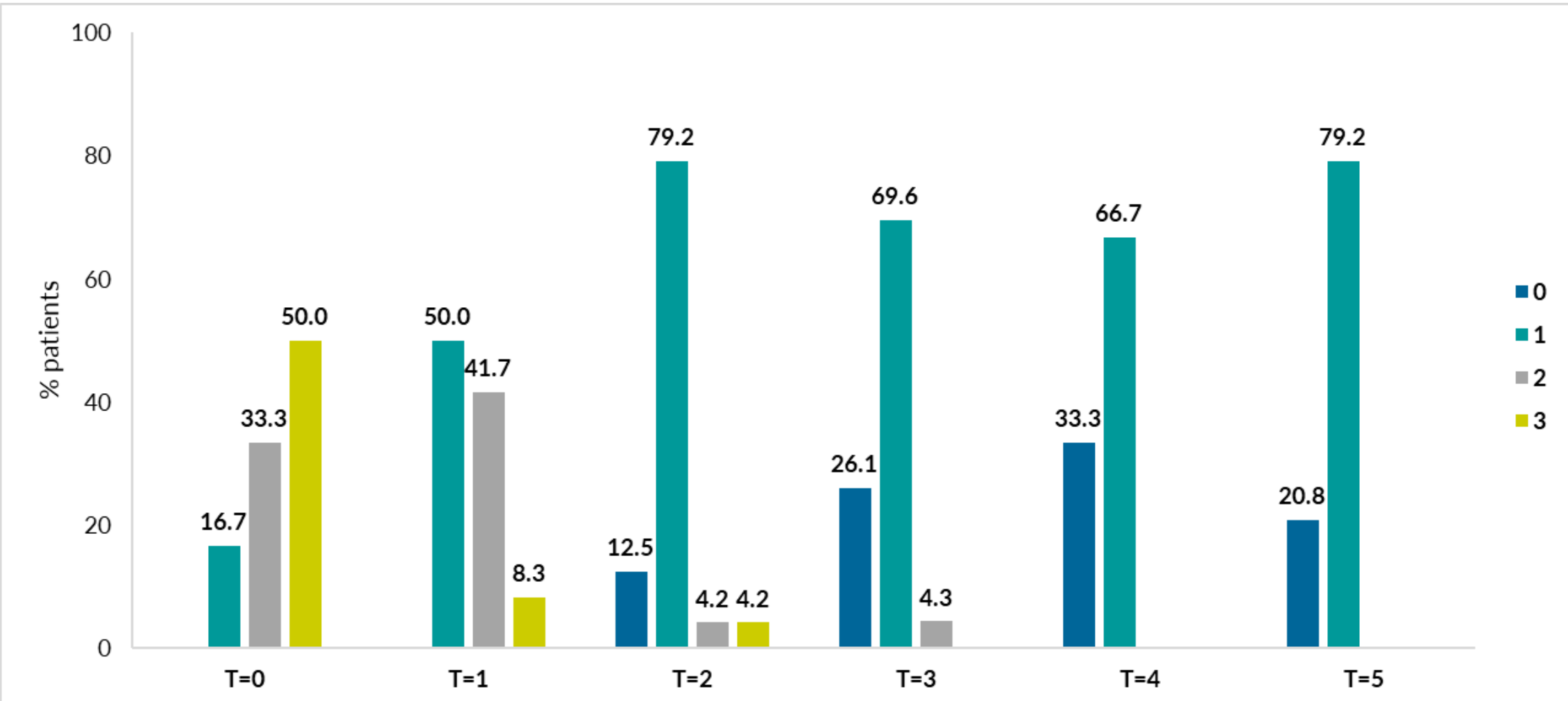

Figure S15. Distribution of patients according to RMS at baseline and after up to five years of treatment in patients with complete five-year AIT data.

Table S12. Distribution of patients across RMS levels and statistical comparison between baseline and follow-up at one, two, three, four and five years of AIT.

		RMS					
	**Score**	**T=0**	**T=1**	**T=2**	**T=3**	**T=4**	**T=5**
	**0**	0	0	3	6	8	5
	**>0**	24	24	21	17	16	19
**χ^2**	-	-	NA	3.2	7.177	9.6	5.581
p	-	-	NA	0.074	0.007	0.002	0.018
**ϕ**	-	-	NA	0.258	0.391	0.447	0.341
**Effect Size**	-	-	NA	Small	Moderate	Moderate	Moderate
	**0 - 1**	4	12	22	22	24	24
	**>1**	20	12	2	1	0	0
**χ^2**	-	-	6.00	27.19	29.64	34.29	34.29
p	-	-	0	<0.001	<0.001	<0.001	<0.001
**ϕ**	-	-	0.354	0.753	0.794	0.845	0.845
**Effect Size**	-	-	Moderate	Large	Large	Large	Large
	**<3**	12	22	23	23	24	24
	**3**	12	2	1	0	0	0
**χ^2**	-	-	10.084	12.765	15.443	16	16
p	-	-	0.001	<0.001	<0.001	<0.001	<0.001
**ϕ**	-	-	0.458	0.516	0.573	0.577	0.577
**Effect Size**	-	-	Moderate	Large	Large	Large	Large

RMS: Rhinoconjunctivitis Medication Score. χ^2: chi-square test; ϕ: Phi coefficient. NA: Not applicable. Effect sizes were as small (ϕ = 0.10), moderate (ϕ = 0.30) or large (ϕ ≥ 0.50). All comparisons were performed using Pearson's χ^2 test.

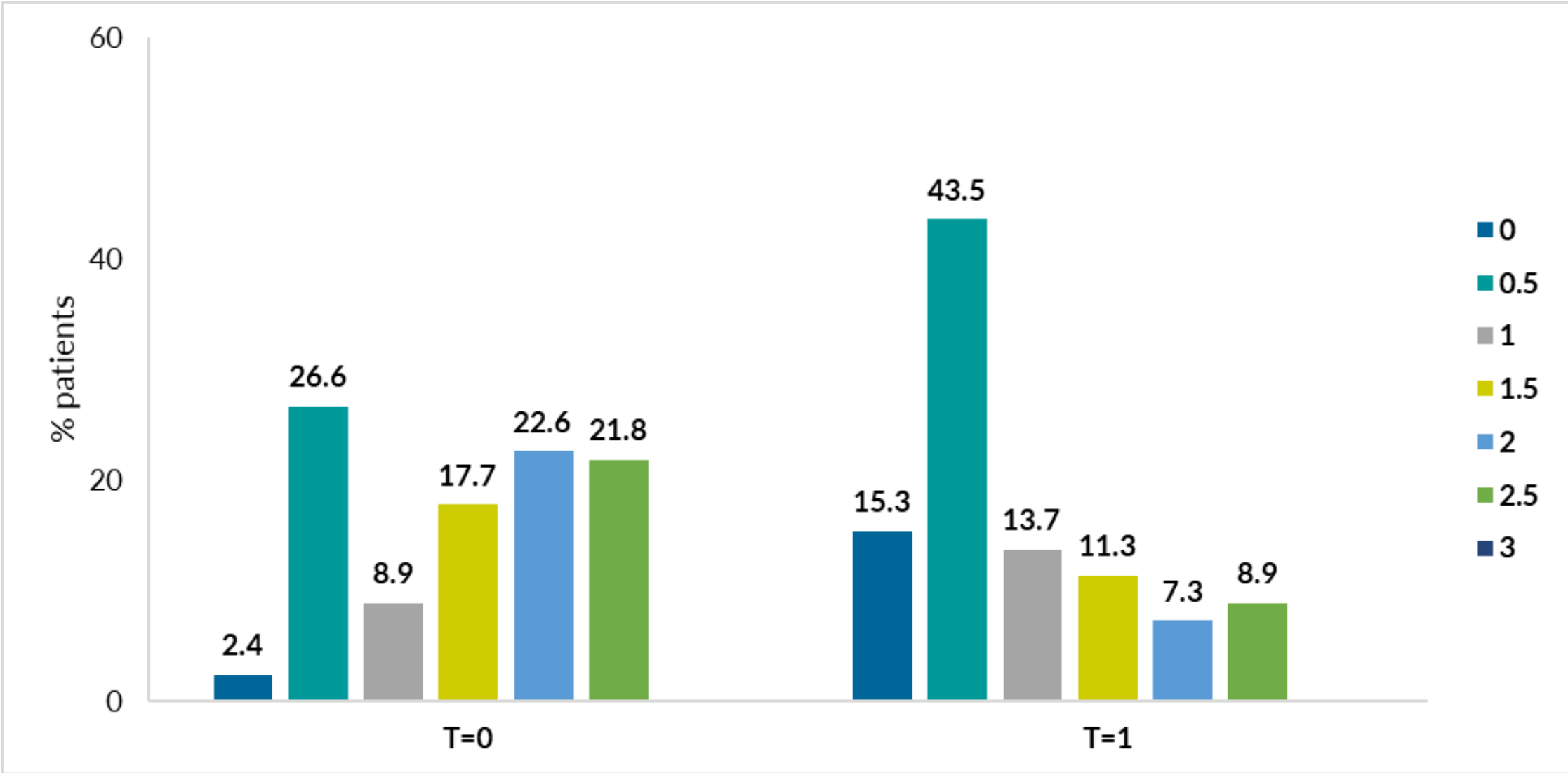

Figure S16. Distribution of patients according to AMS at baseline and after one year of AIT.

Table S13. Distribution of patients across AMS levels and statistical comparison between baseline and follow-up at one year of AIT.

		AMS	
	**Score**	**T=0**	**T=1**
	**0**	3	19
	**>0**	121	105
**χ^2**	-	-	12.769
p	-	-	<0.001
**ϕ**	-	-	0.227
**Effect Size**	-	-	Small
	**0 - 0.5**	36	73
	**>0.5**	88	51
**χ^2**	-	-	22.41
p	-	-	<0.001
**ϕ**	-	-	0.301
**Effect Size**	-	-	Moderate
	**0 - 1**	47	90
	**1.5 - 3**	77	34
**χ^2**	-	-	30.154
p	-	-	<0.001
**ϕ**	-	-	0.349
**Effect Size**	-	-	Moderate
	**0 - 2**	97	113
	**2.5 - 3**	27	11
**χ^2**	-	-	7.96
p	-	-	0
**ϕ**	-	-	0.179
**Effect Size**	-	-	Small

AMS: Asthma Medication Score. χ^2: chi-square test; ϕ: Phi coefficient.
Effect sizes were as small (ϕ = 0.10), moderate (ϕ = 0.30) or large (ϕ ≥ 0.50). All comparisons were performed using Pearson's χ^2 test.

Table S14. Descriptive statistics of the AMS in the subset of patients who completed two years of AIT.

	AMS		
	T=0	T=1	T=2
**Mean**	1.51	0.92	0.60
**95% lower CI**	1.32	0.74	0.45
**95% upper CI**	1.69	1.11	0.74
**SD**	0.79	0.81	0.60
**Median**	1.50	0.50	0.50
**Q1**	0.75	0.50	0.00
**Q3**	2.00	1.50	0.75
**Maximum**	2.50	2.50	2.50
**Minimum**	0.00	0.00	0.00
**n**	73	73	73

AMS: Asthma Medication Score. CI: Confidence Interval. SD: Standard Deviation. Q1: First quartile. Q3: Third quartile.

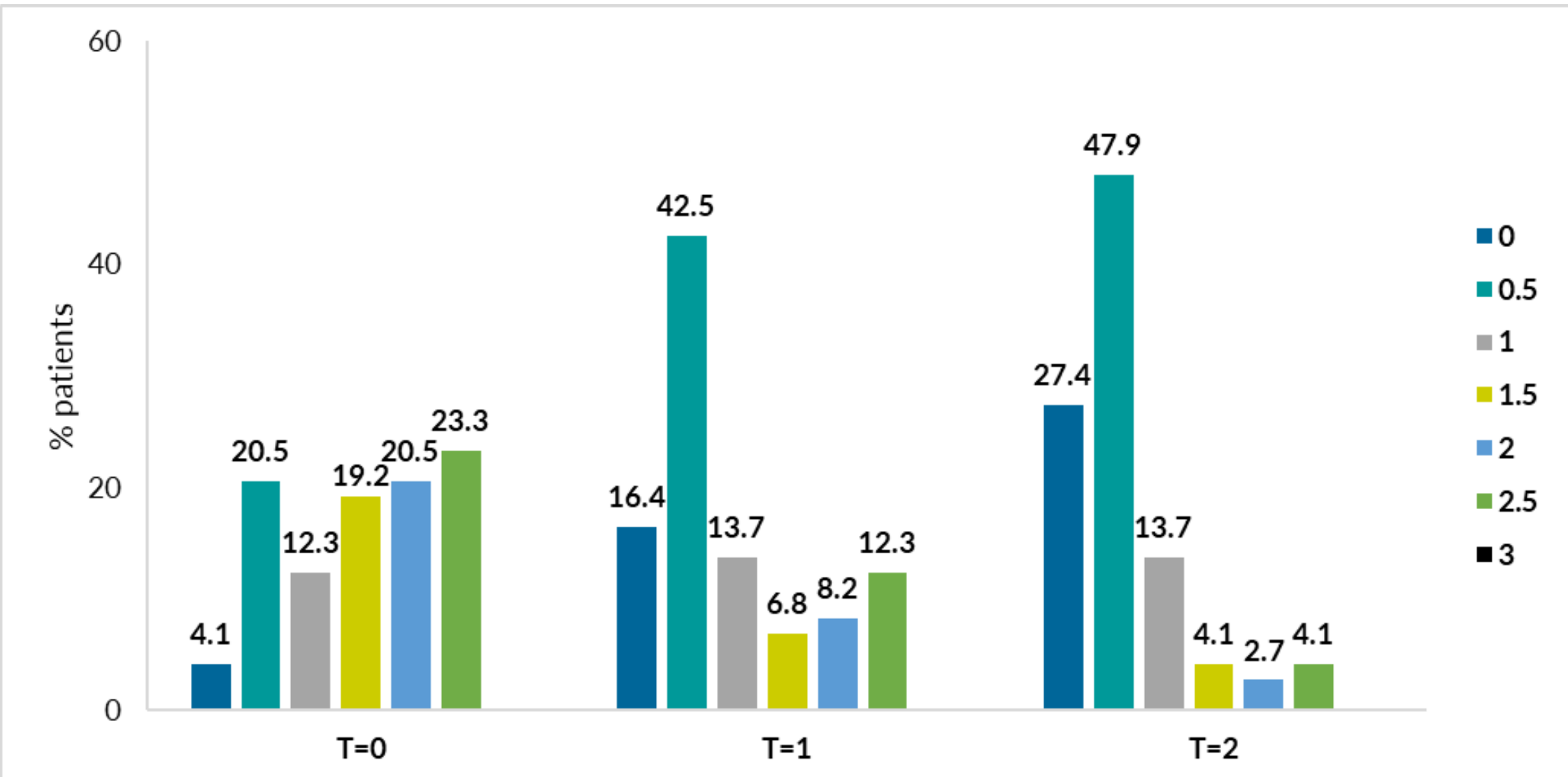

Figure S17. Distribution of patients according to AMS at baseline and after one and two years of AIT, in patients with complete two-year AIT data.

Table S15. Distribution of patients across AMS levels and statistical comparison between baseline and follow-up at one and two years of AIT.

		AMS		
	**Score**	**T=0**	**T=1**	**T=2**
	**0**	3	12	20
	**>0**	70	61	53
**χ^2**	-	-	6.018	14.915
p	-	-	0.014	<0.001
**ϕ**	-	-	0.203	0.32
**Effect Size**	-	-	Small	Moderate
	**0 - 0.5**	18	43	55
	**>0.5**	55	30	18
**χ^2**	-	-	17.60	37.51
p	-	-	<0.001	<0.001
**ϕ**	-	-	0.347	0.507
**Effect Size**	-	-	Moderate	Large
	**0 - 1**	27	53	65
	**1.5 - 3**	46	20	8
**χ^2**	-	-	18.692	42.436
p	-	-	<0.001	<0.001
**ϕ**	-	-	0.358	0.539
**Effect Size**	-	-	Moderate	Large
	**0 - 2**	56	64	70
	**2.5 - 3**	17	9	3
**χ^2**	-	-	3.00	11.36
p	-	-	0	<0.001
**ϕ**	-	-	0.143	0.279
**Effect Size**	-	-	Small	Small

AMS: Asthma Medication Score. χ^2: chi-square test; ϕ: Phi coefficient.
Effect sizes were as small (ϕ = 0.10), moderate (ϕ = 0.30) or large (ϕ ≥ 0.50). All comparisons were performed using Pearson's χ^2 test.

Table S16. Descriptive statistics of the AMS in the subset of patients who completed three years of AIT.

	AMS			
	**T=0**	**T=1**	**T=2**	**T=3**
**Mean**	1.47	0.89	0.59	0.49
**95% lower CI**	1.27	0.69	0.44	0.33
**95% upper CI**	1.66	1.08	0.75	0.66
**SD**	0.77	0.74	0.60	0.65
**Median**	1.5	0.5	0.5	0.5
**Q1**	0.5	0.5	0	0
**Q3**	2	1.25	0.875	0.5
**Maximum**	2.5	2.5	2.5	2.5
**Minimum**	0	0	0	0
**n**	61	61	60	61

AMS: Asthma Medication Score. CI: Confidence Interval. SD: Standard Deviation. Q1: First quartile. Q3: Third quartile.

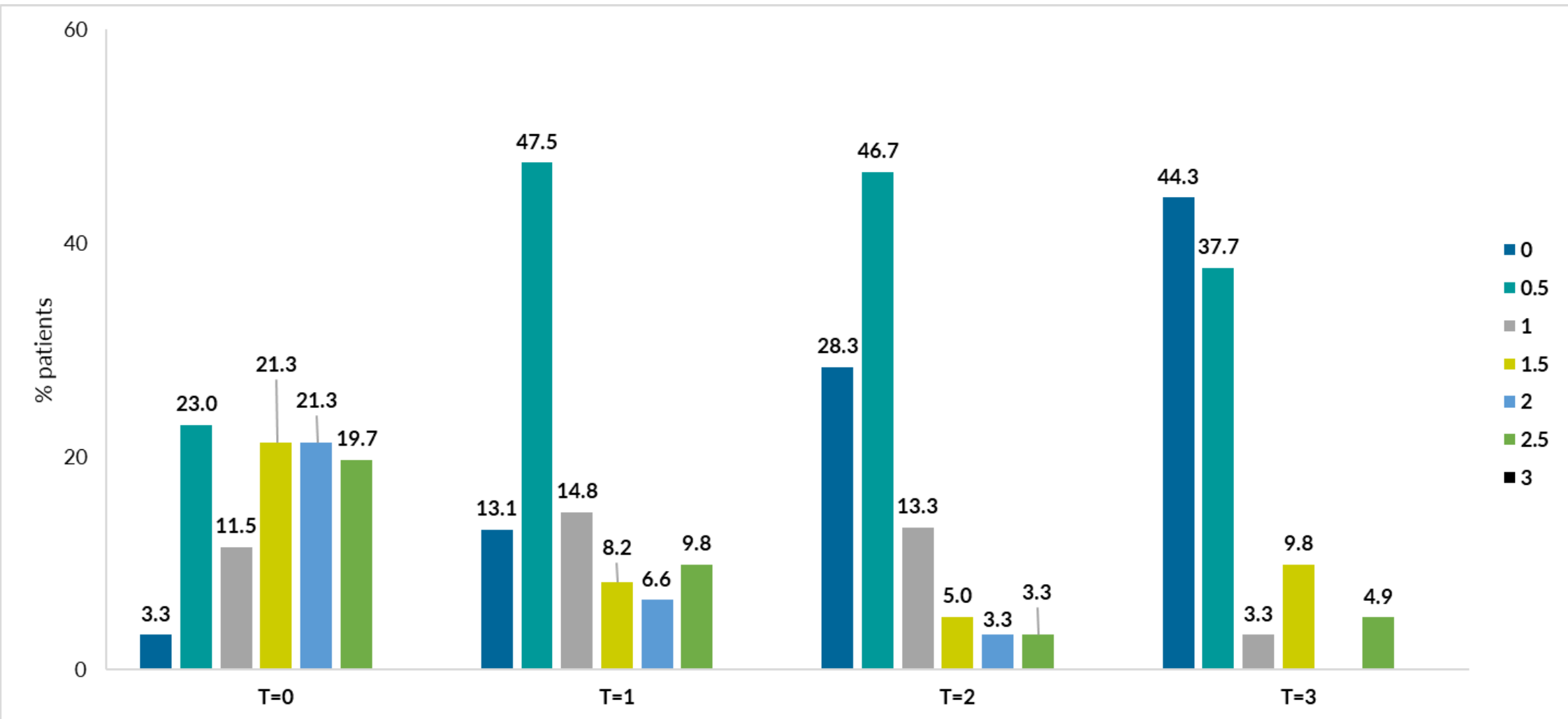

Figure S18. Distribution of patients according to AMS at baseline and after up to three years of AIT in patients with complete three-year AIT data.

Table S17. Distribution of patients across AMS levels and statistical comparison between baseline and follow-up at one, two and three years of AIT.

		AMS			
	**Score**	**T=0**	**T=1**	**T=2**	**T=3**
	**0**	2	8	17	27
	**>0**	59	53	43	34
**χ^2**	-	-	3.921	14.345	28.272
p	-	-	0.048	<0.001	<0.001
**ϕ**	-	-	0.179	0.344	0.481
**Effect Size**	-	-	Small	Moderate	Moderate
	**0 - 0.5**	16	37	45	50
	**>0.5**	45	24	15	11
**χ^2**	-	-	14.71	28.78	38.16
p	-	-	<0.001	<0.001	<0.001
**ϕ**	-	-	0.347	0.488	0.559
**Effect Size**	-	-	Moderate	Moderate	Large
	**0 - 1**	23	46	53	52
	**1.5 - 3**	38	15	7	9
**χ^2**	-	-	17.648	33.192	29.107
p	-	-	<0.001	<0.001	<0.001
**ϕ**	-	-	0.38	0.524	0.488
**Effect Size**	-	-	Moderate	Large	Moderate
	**0 - 2**	49	55	58	58
	**2.5 - 3**	12	6	2	3
**χ^2**	-	-	2.35	7.89	6.16
p	-	-	0	0	0
**ϕ**	-	-	0.139	0.255	0.225
**Effect Size**	-	-	Small	Small	Small

AMS: Asthma Medication Score. χ^2: chi-square test; ϕ: Phi coefficient.
Effect sizes were as small (ϕ = 0.10), moderate (ϕ = 0.30) or large (ϕ ≥ 0.50). All comparisons were performed using Pearson's χ^2 test.

Table S18. Descriptive statistics of the AMS in the subset of patients who completed four years of AIT.

	AMS				
	T=0	T=1	T=2	T=3	T=4
**Mean**	1.47	0.72	0.56	0.47	0.31
**95% lower CI**	1.24	0.53	0.39	0.27	0.14
**95% upper CI**	1.70	0.91	0.72	0.67	0.47
**SD**	0.68	0.55	0.49	0.60	0.48
**Median**	1.5	0.5	0.5	0.5	0
**Q1**	1	0.5	0	0	0
**Q3**	2	1	1	0.5	0.5
**Maximum**	2.5	2	2	2.5	1.5
**Minimum**	0.5	0	0	0	0
**n**	36	36	36	36	36

AMS: Asthma Medication Score. CI: Confidence Interval. SD: Standard Deviation. Q1: First quartile. Q3: Third quartile.

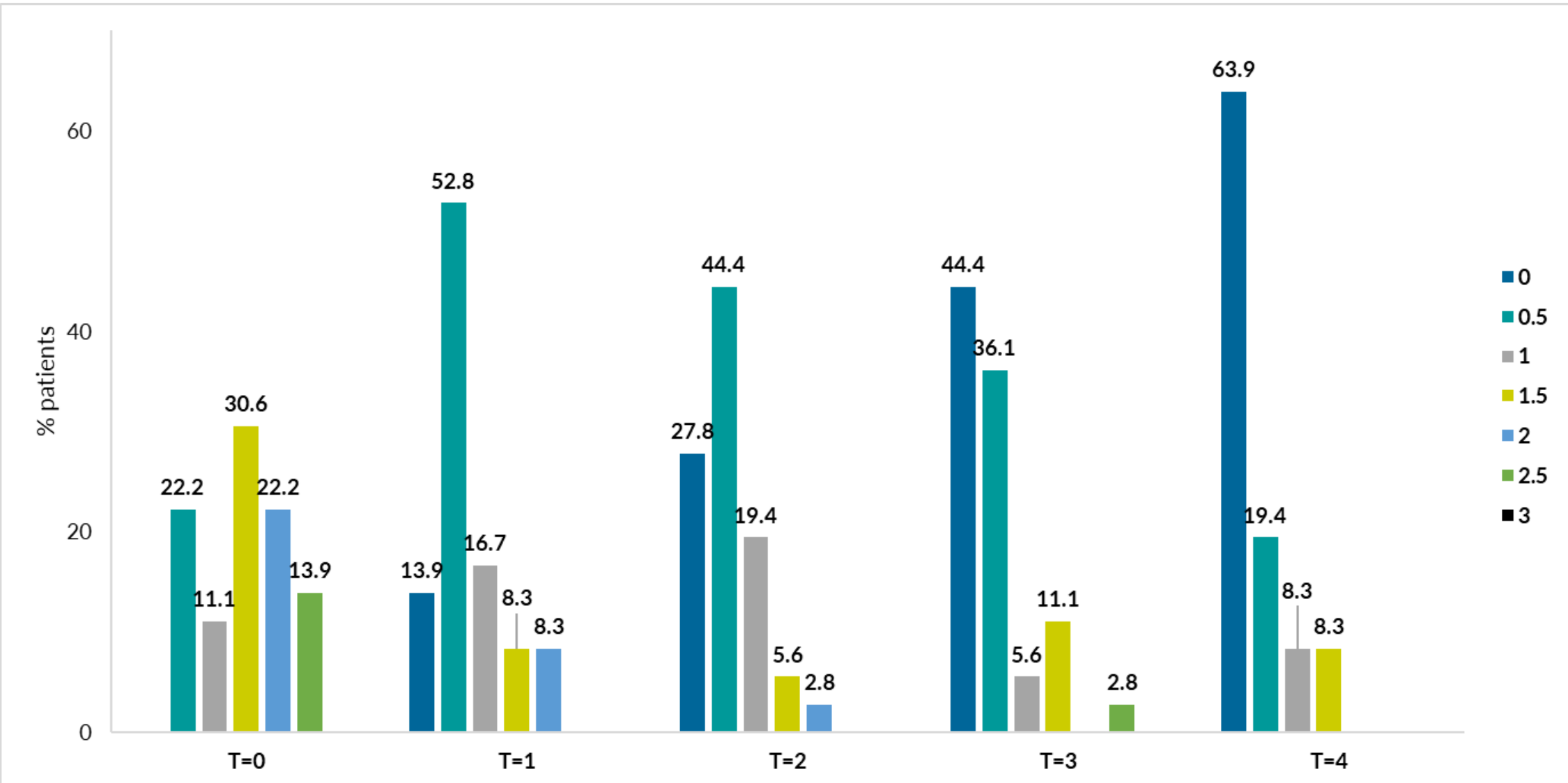

Figure S19. Distribution of patients according to AMS at baseline and after up to four years of AIT in patients with complete four-year AIT data.

Table S19. Distribution of patients across AMS levels and statistical comparison between baseline and follow-up at one, two, three and four years of AIT.

		AMS				
	**Score**	**T=0**	**T=1**	**T=2**	**T=3**	**T=4**
	**0**	0	5	10	16	23
	**>0**	36	31	26	20	13
**χ^2**	-	-	5.373	11.613	20.571	33.796
p	-	-	0.02	<0.001	<0.001	<0.001
**ϕ**	-	-	0.273	0.402	0.535	0.685
**Effect Size**	-	-	Small	Moderate	Large	Large
	**0 - 0.5**	8	24	26	29	30
	**>0.5**	28	12	10	7	6
**χ^2**	-	-	14.40	18.06	24.52	26.97
p	-	-	<0.001	<0.001	<0.001	<0.001
**ϕ**	-	-	0.447	0.501	0.584	0.612
**Effect Size**	-	-	Moderate	Large	Large	Large
	**0 - 1**	12	30	33	31	33
	**1.5 - 3**	24	6	3	5	3
**χ^2**	-	-	18.514	26.133	20.844	26.133
p	-	-	<0.001	<0.001	<0.001	<0.001
**ϕ**	-	-	0.507	0.602	0.538	0.602
**Effect Size**	-	-	Large	Large	Large	Large
	**0 - 2**	31	36	36	35	36
	**2.5 - 3**	5	0	0	1	0
**χ^2**	-	-	5.37	5.37	2.91	5.37
p	-	-	0	0	0	0
**ϕ**	-	-	0.273	0.273	0.201	0.273
**Effect Size**	-	-	Small	Small	Small	Small

AMS: Asthma Medication Score. χ^2: chi-square test; ϕ: Phi coefficient.
Effect sizes were as small (ϕ = 0.10), moderate (ϕ = 0.30) or large (ϕ ≥ 0.50). All comparisons were performed using Pearson's χ^2 test.

Table S20. Descriptive statistics of the AMS in the subset of patients who completed five years of AIT.

	AMS					
	T=0	T=1	T=2	T=3	T=4	T=5
**Mean**	1.41	0.82	0.74	0.71	0.50	0.41
**95% lower CI**	1.07	0.50	0.45	0.32	0.20	0.12
**95% upper CI**	1.75	1.15	1.02	1.09	0.80	0.70
**SD**	0.67	0.64	0.56	0.75	0.59	0.57
**Median**	1.5	0.5	0.5	0.5	0.5	0
**Q1**	0.75	0.5	0.5	0	0	0
**Q3**	2	1.5	1	1.5	1	1
**Maximum**	2.5	2	2	2.5	1.5	1.5
**Minimum**	0.5	0	0	0	0	0
**n**	17	17	17	17	17	17

AMS: Asthma Medication Score. CI: Confidence Interval. SD: Standard Deviation. Q1: First quartile. Q3: Third quartile.

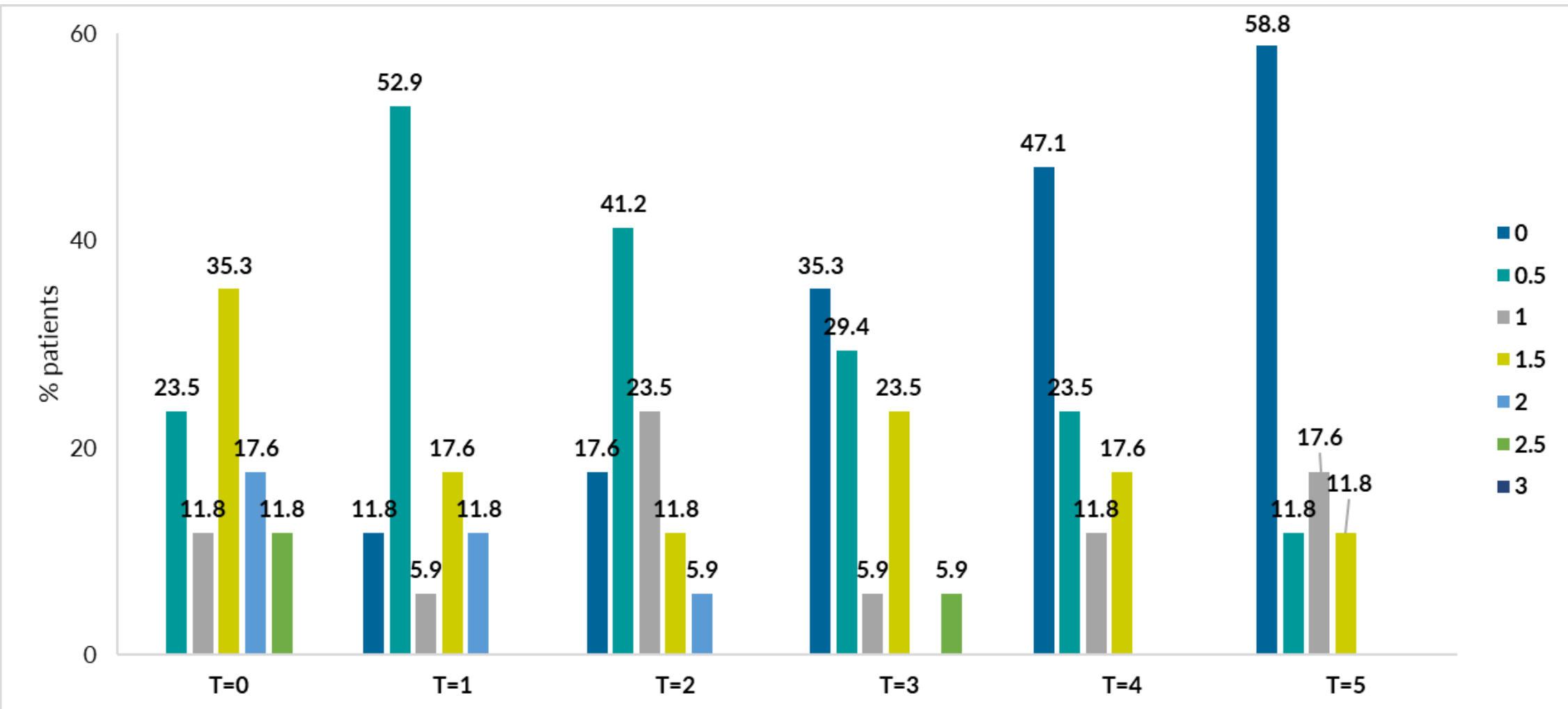

Figure S20. Distribution of patients according to AMS at baseline and after up to five years of AIT in patients with complete five-year AIT data.

Table S21. Distribution of patients across AMS levels and statistical comparison between baseline and follow-up at one, two, three, four and five years of AIT.

		AMS					
	**Score**	**T=0**	**T=1**	**T=2**	**T=3**	**T=4**	**T=5**
	**0**	0	2	3	6	8	10
	**>0**	17	15	14	11	9	7
**χ^2**	-	-	2.125	3.29	7.286	10.462	14.167
p	-	-	0.145	0.07	0.007	0.001	<0.001
**ϕ**	-	-	0.25	0.311	0.463	0.555	0.645
**Effect Size**	-	-	Small	Moderate	Moderate	Large	Large
	**0 - 0.5**	4	11	10	11	12	12
	**>0.5**	13	6	7	6	5	5
**χ^2**	-	-	5.846	4.371	5.846	7.556	7.556
p	-	-	0.016	0.037	0.016	0.006	0.006
**ϕ**	-	-	0.415	0.359	0.415	0.471	0.471
**Effect Size**	-	-	Moderate	Moderate	Moderate	Moderate	Moderate
	**0 - 1**	6	12	14	12	14	15
	**1.5 - 3**	11	5	3	5	3	2
**χ^2**	-	-	4.25	7.771	4.25	7.771	10.088
p	-	-	0.039	0.005	0.039	0.005	0.001
**ϕ**	-	-	0.354	0.478	0.354	0.478	0.545
**Effect Size**	-	-	Moderate	Moderate	Moderate	Moderate	Large
	**0 - 2**	15	17	17	16	17	17
	**2.5 - 3**	2	0	0	1	0	0
**χ^2**	-	-	2.125	2.125	0.366	2.125	2.125
p	-	-	0.145	0.145	0.545	0.145	0.145
**ϕ**	-	-	0.25	0.25	0.104	0.25	0.25
**Effect Size**	-	-	Small	Small	Small	Small	Small

AMS: Asthma Medication Score. χ^2: chi-square test; ϕ: Phi coefficient.
Effect sizes were as small (ϕ = 0.10), moderate (ϕ = 0.30) or large (ϕ ≥ 0.50). All comparisons were performed using Pearson's χ^2 test.

Table S22. Descriptive statistics of the RCSMS after one year of AIT.

	RCSMS	
	**T0**	**T1**
**Mean**	3.94	2.23
**95% lower CI**	3.82	2.09
**95% upper CI**	4.05	2.37
**SD**	0.84	1.02
**Median**	4.00	2.17
**Q1**	3.50	1.67
**Q3**	4.67	2.83
**Maximum**	5.83	5.50
**Minimum**	0.17	0.00
**n**	208	208
**Reduction vs. $T0_{Median}$**	–	45.8%

CI: Confidence Interval. SD: Standard Deviation. Q1: First quartile. Q3: Third quartile. RCSMS: Rhinoconjunctivitis Combined Symptom and Medication Score.

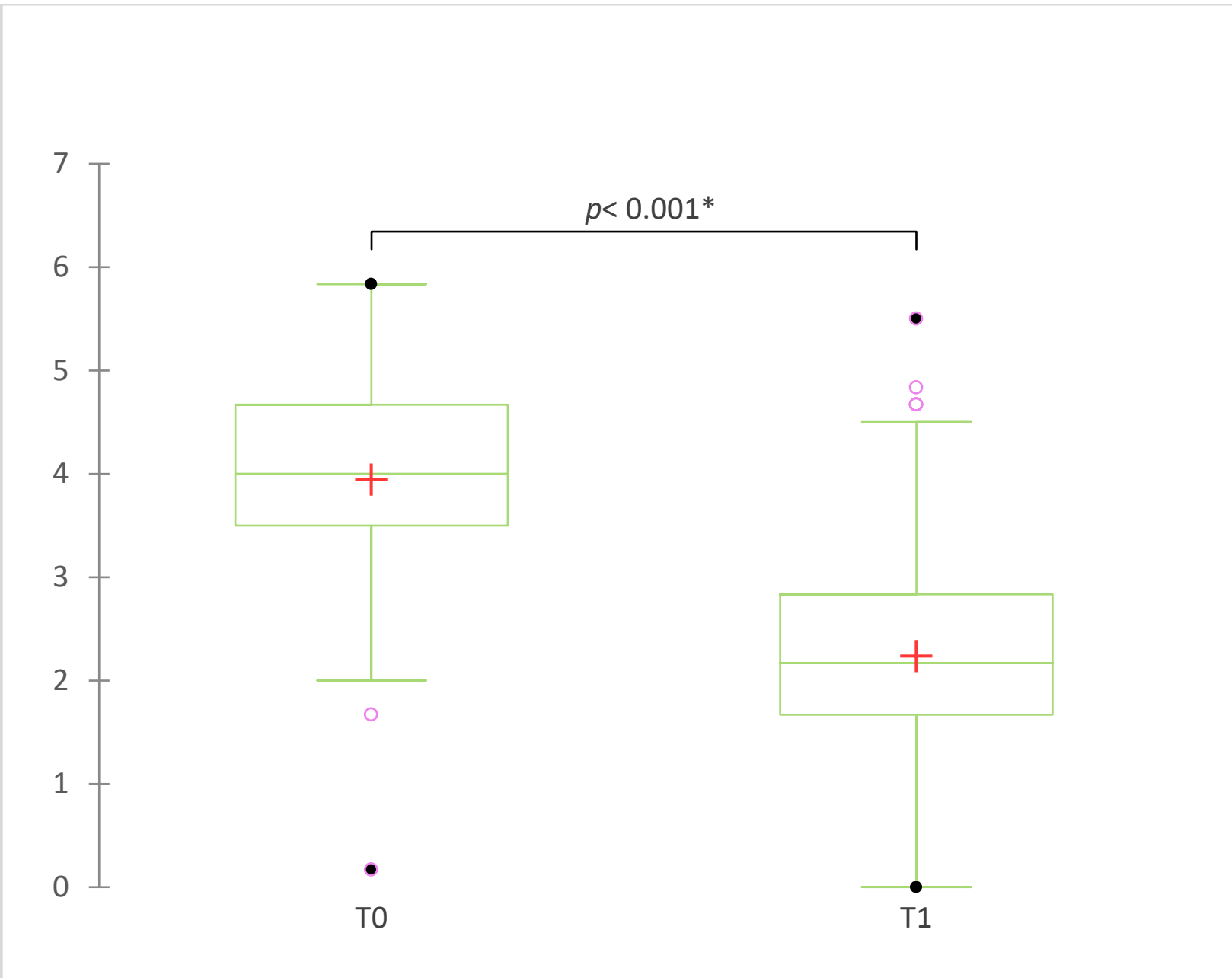

Figure S21. Evolution of the RCSMS during the first year of AIT.
Boxes represent the interquartile range (IQR), horizontal lines indicate medians, and whiskers denote minimum and maximum values. Red crosses represent mean values. Pink symbols represent atypical values. *Reaching statistical significance at level α≤0.05. RCSMS: Rhinoconjunctivitis Combined Symptom and Medication Score.

Table S23. Distribution of patients across RCSMS levels and statistical comparison between baseline and follow-up at one year of AIT.

		RCSMS	
	**Score**	**T=0**	**T=1**
	**0**	0	6
	**>0**	208	202
**χ^2**	-	-	6.09
p	-	-	0.014
**ϕ**	-	-	0.121
**Effect Size**	-	-	Small
	**≤1**	1	20
	**>1**	207	188
**χ^2**	-	-	18.10
p	-	-	<0.001
**ϕ**	-	-	0.209
**Effect Size**	-	-	Small
	**≤1.5**	1	43
	**>1.5**	207	165
**χ^2**	-	-	44.833
p	-	-	<0.001
**ϕ**	-	-	0.328
**Effect Size**	-	-	Moderate
	**≤2**	5	103
	**>2**	203	105
**χ^2**	-	-	120.11
p	-	-	<0.001
**ϕ**	-	-	0.537
**Effect Size**	-	-	Large

RCSMS: Rhinoconjunctivitis Combined Symptom and Medication Score. χ^2: chi-square test; ϕ: Phi coefficient.
Effect sizes were as small (ϕ = 0.10), moderate (ϕ = 0.30) or large (ϕ ≥ 0.50). All comparisons were performed using Pearson's χ^2 test.

Table S24. Descriptive statistics of the RCSMS in the subset of patients who completed two years of AIT.

	RCSMS		
	**T0**	**T1**	**T2**
**Mean**	4.07	2.37	1.73
**95% lower CI**	3.91	2.19	1.57
**95% upper CI**	4.23	2.54	1.90
**SD**	0.89	1.00	0.93
**Median**	4.00	2.25	1.58
**Q1**	3.50	1.67	1.33
**Q3**	4.83	3.00	2.00
**Maximum**	5.83	5.50	4.67
**Minimum**	0.17	0.00	0.00
**n**	126	126	126
**Reduction vs. $T0_{Median}$**	–	43.8%	60.4%

CI: Confidence Interval. SD: Standard Deviation. Q1: First quartile. Q3: Third quartile. RCSMS: Rhinoconjunctivitis Combined Symptom and Medication Score.

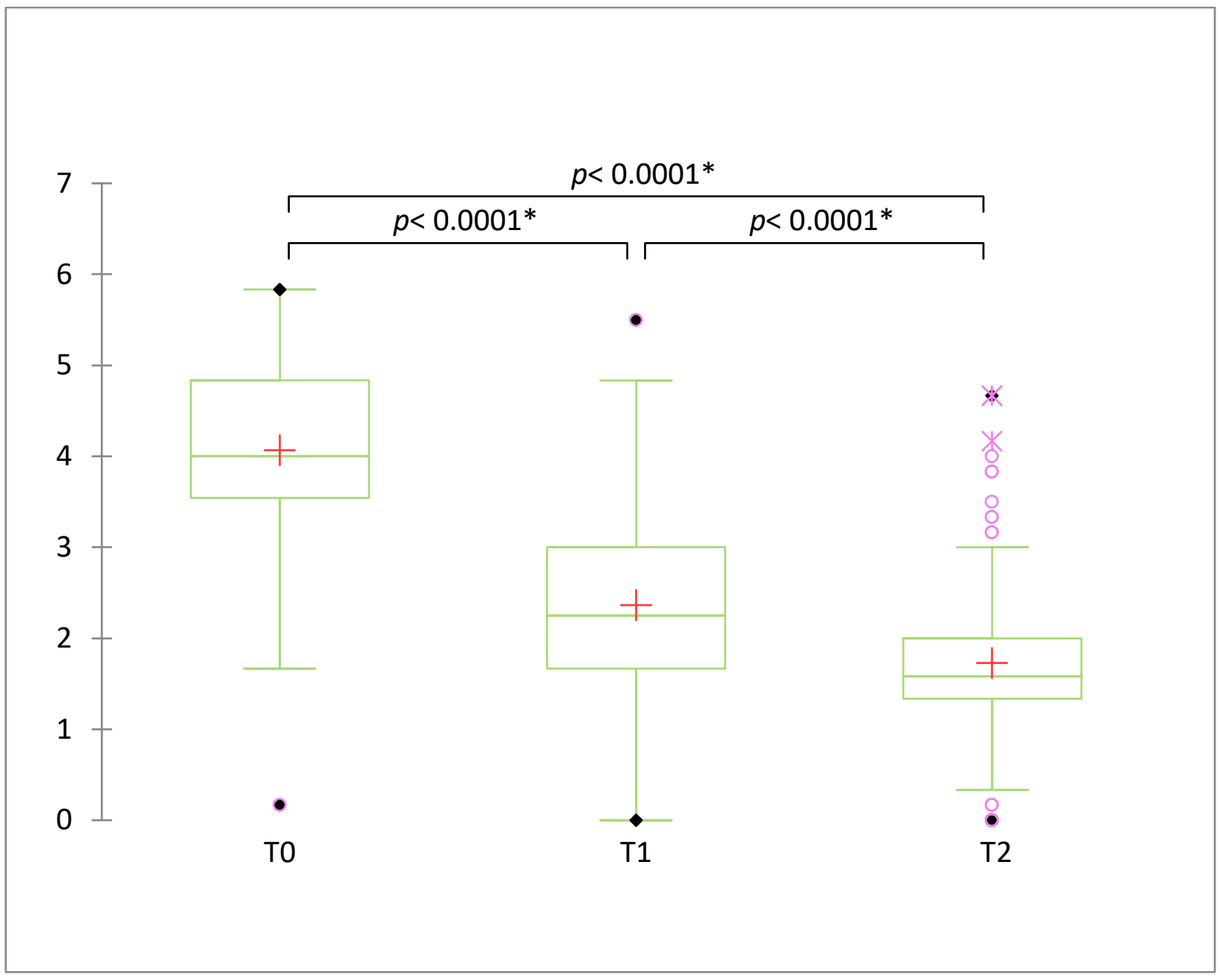

Figure S22. Evolution of the RCSMS over two years of AIT.
Boxes represent the interquartile range (IQR), horizontal lines indicate medians, and whiskers denote minimum and maximum values. Red crosses represent mean values. Pink symbols represent atypical values. *Reaching statistical significance at level α≤0.05. RCSMS: Rhinoconjunctivitis Combined Symptom and Medication Score.

Table S25. Distribution of patients across RCSMS levels and statistical comparison between baseline and follow-up at one and two years of AIT.

		RCSMS		
	**Score**	**T=0**	**T=1**	**T=2**
	**0**	0	2	7
	**>0**	126	124	119
**χ²**	-	-	2.016	7.2
p	-	-	0.156	0.007
**ϕ**	-	-	0.089	0.169
**Effect Size**	-	-	Ineligible	Small
	**≤1**	1	8	17
	**>1**	125	118	109
**χ²**	-	-	5.65	15.32
p	-	-	0	<0.001
**ϕ**	-	-	0.15	0.247
**Effect Size**	-	-	Small	Small
	**≤1.5**	1	23	63
	**>1.5**	125	103	63
**χ²**	-	-	22.289	80.509
p	-	-	<0.001	<0.001
**ϕ**	-	-	0.297	0.565
**Effect Size**	-	-	Small	Large
	**≤2**	3	58	96
	**>2**	123	68	30
**χ²**	-	-	65.43	143.89
p	-	-	<0.001	<0.001
**ϕ**	-	-	0.51	0.756
**Effect Size**	-	-	Large	Large

RCSMS: Rhinoconjunctivitis Combined Symptom and Medication Score. χ^2: chi-square test; ϕ: Phi coefficient.
Effect sizes were as small (ϕ = 0.10), moderate (ϕ = 0.30) or large (ϕ ≥ 0.50). All comparisons were performed using Pearson's χ^2 test.

Table S26. Descriptive statistics of the RCSMS in the subset of patients who completed three years of AIT.

	RCSMS			
	T0	T1	T2	T3
**Mean**	4.14	2.31	1.63	1.31
**95% lower CI**	3.96	2.12	1.46	1.14
**95% upper CI**	4.32	2.50	1.80	1.48
**SD**	0.90	0.96	0.86	0.89
**Median**	4.17	2.17	1.50	1.33
**Q1**	3.67	1.67	1.25	1.00
**Q3**	4.83	3.00	2.00	1.50
**Maximum**	5.83	5.50	4.67	4.33
**Minimum**	0.17	0.00	0.00	0.00
**n**	104	104	101	104
**Reduction vs. $T0_{Median}$**	–	48.0%	64.0%	68.0%

CI: Confidence Interval. SD: Standard Deviation. Q1: First quartile. Q3: Third quartile. RCSMS: Rhinoconjunctivitis Combined Symptom and Medication Score.

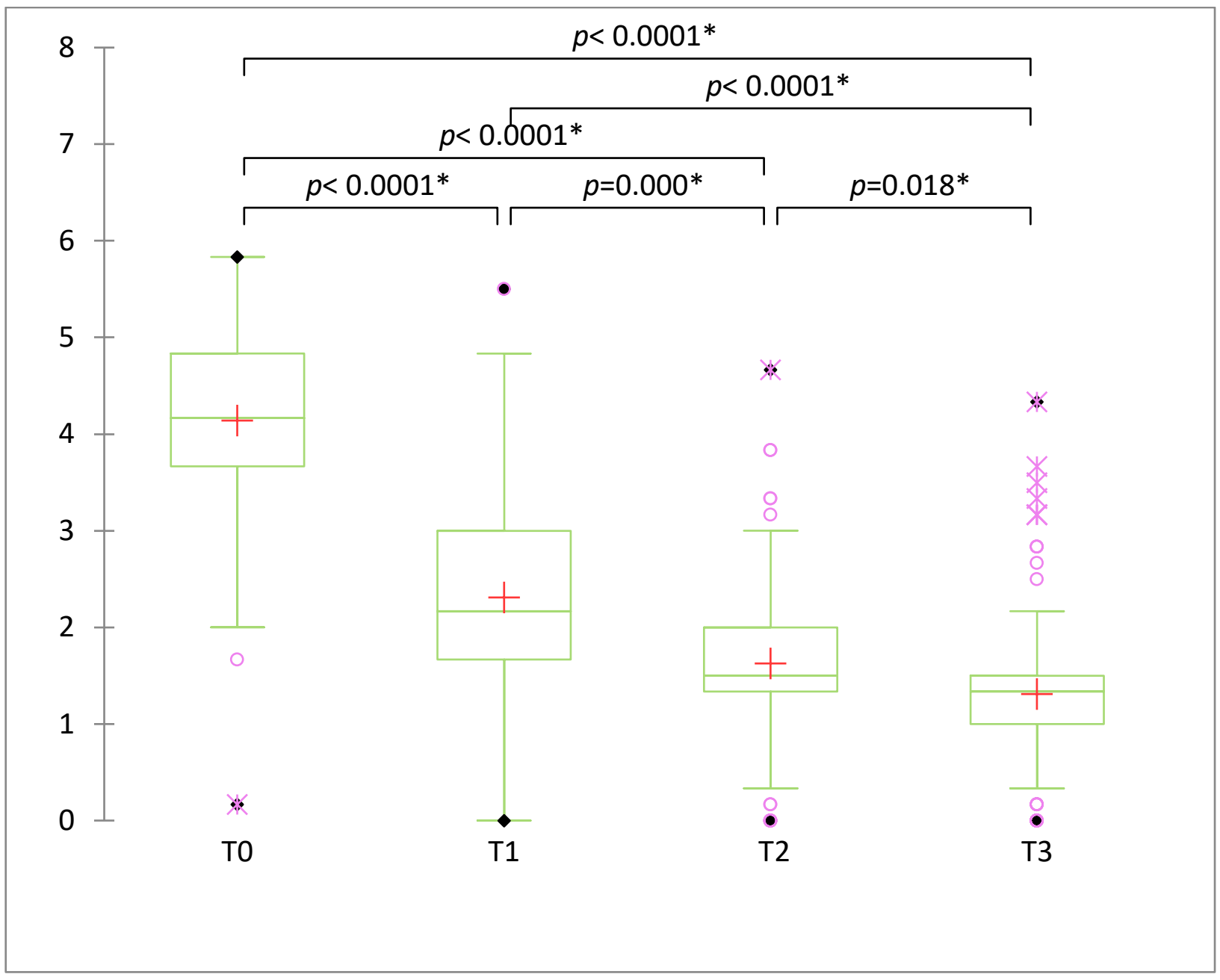

Figure S23. Evolution of the RCSMS over three years of AIT.
Boxes represent the interquartile range (IQR), horizontal lines indicate medians, and whiskers denote minimum and maximum values. Red crosses represent mean values. Pink symbols represent atypical values. *Reaching statistical significance at level α≤0.05. RCSMS: Rhinoconjunctivitis Combined Symptom and Medication Score.

Table S27. Distribution of patients across RCSMS levels and statistical comparison between baseline and follow-up at one, two and three years of AIT.

		RCSMS			
	**Score**	**T=0**	**T=1**	**T=2**	**T=3**
	**0**	0	2	6	12
	**>0**	104	102	98	92
**χ^2**	-	-	2.019	6.178	12.735
p	-	-	0.155	0.013	<0.001
**ϕ**	-	-	0.099	0.172	0.247
**Effect Size**	-	-	Ineligible	Small	Small
	**≤1**	1	7	16	29
	**>1**	103	97	88	75
**χ^2**	-	-	4.68	14.41	30.54
p	-	-	0	<0.001	<0.001
**ϕ**	-	-	0.15	0.263	0.383
**Effect Size**	-	-	Small	Small	Moderate
	**≤1.5**	1	20	55	79
	**>1.5**	103	84	49	25
**χ^2**	-	-	19.121	71.256	123.581
p	-	-	<0.001	<0.001	<0.001
**ϕ**	-	-	0.303	0.585	0.771
**Effect Size**	-	-	Moderate	Large	Large
	**≤2**	3	50	81	91
	**>2**	101	54	23	13
**χ^2**	-	-	55.93	121.49	150.31
p	-	-	<0.001	<0.001	<0.001
**ϕ**	-	-	0.519	0.764	0.85
**Effect Size**	-	-	Large	Large	Large

RCSMS: Rhinoconjunctivitis Combined Symptom and Medication Score. χ^2: chi-square test; ϕ: Phi coefficient.
Effect sizes were as small (ϕ = 0.10), moderate (ϕ = 0.30) or large (ϕ ≥ 0.50). All comparisons were performed using Pearson's χ^2 test.

Table S28. Descriptive statistics of the RCSMS in the subset of patients who completed four years of AIT.

	RCSMS				
	**T0**	**T1**	**T2**	**T3**	**T4**
**Mean**	4.21	2.29	1.60	1.25	1.02
**95% lower CI**	3.99	2.03	1.38	1.05	0.79
**95% upper CI**	4.44	2.54	1.82	1.45	1.25
**SD**	0.91	1.02	0.89	0.81	0.94
**Median**	4.33	2.00	1.50	1.33	1.17
**Q1**	3.67	1.67	1.33	1.04	0.17
**Q3**	5.00	3.00	1.83	1.50	1.42
**Maximum**	5.83	5.50	4.67	4.33	5.17
**Minimum**	1.67	0.00	0.00	0.00	0.00
**n**	65	65	63	64	65
**Reduction vs. $T0_{Median}$**	–	53.8%	65.4%	69.2%	73.1%

CI: Confidence Interval. SD: Standard Deviation. Q1: First quartile. Q3: Third quartile. RCSMS: Rhinoconjunctivitis Combined Symptom and Medication Score.

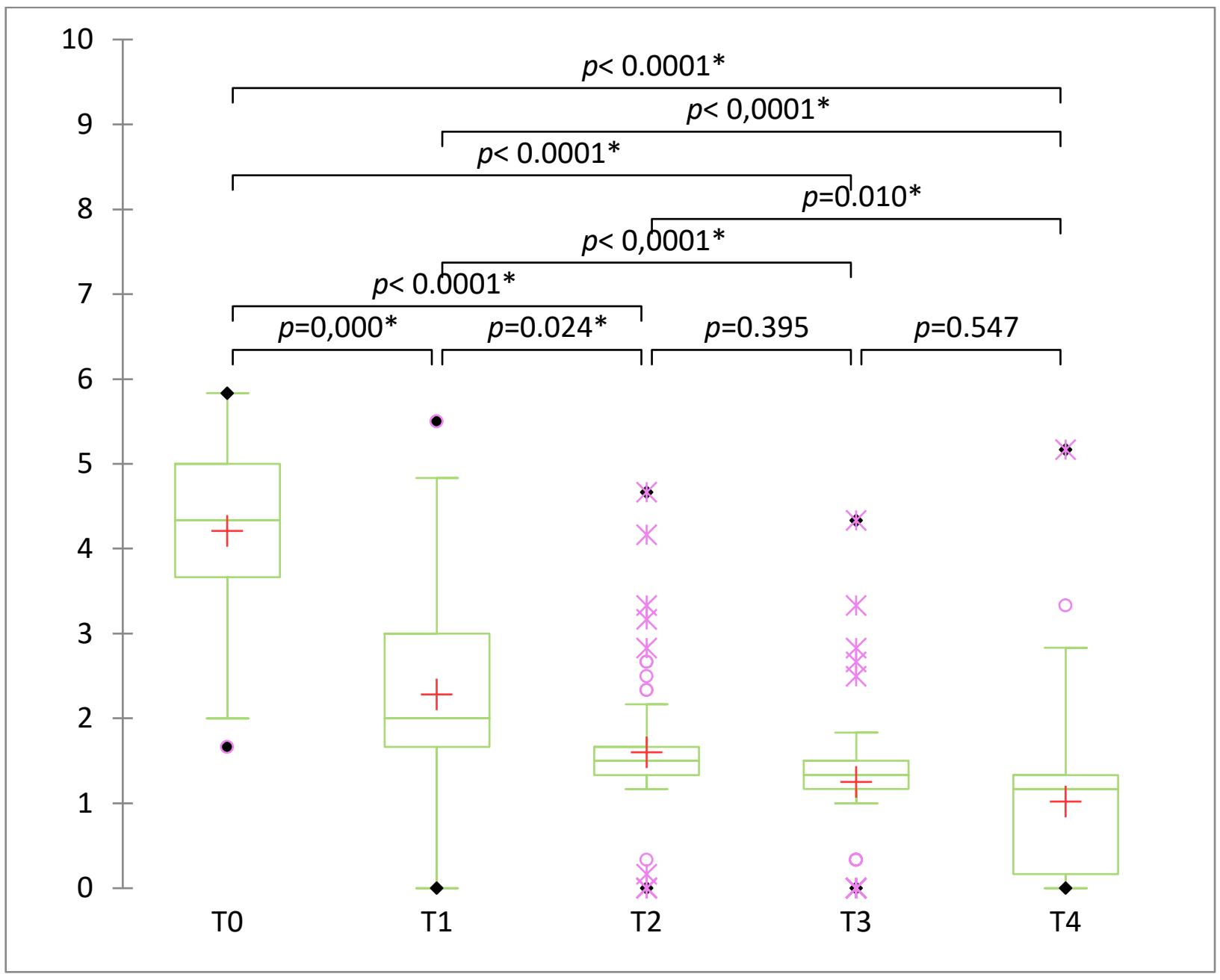

Figure S24. Evolution of the RCSMS over four years of AIT.
Boxes represent the interquartile range (IQR), horizontal lines indicate medians, and whiskers denote minimum and maximum values. Red crosses represent mean values. Pink symbols represent atypical values. *Reaching statistical significance at level α≤0.05. RCSMS: Rhinoconjunctivitis Combined Symptom and Medication Score.

Table S29. Distribution of patients across RCSMS levels and statistical comparison between baseline and follow-up at one, two, three and four years of AIT.

		RCSMS				
	**Score**	**T=0**	**T=1**	**T=2**	**T=3**	**T=4**
	**0**	0	2	6	9	10
	**>0**	65	63	59	56	55
**χ^2**	-	-	2.031	6.29	9.669	10.833
p	-	-	0.154	0.012	0.002	<0.001
**ϕ**	-	-	0.125	0.22	0.273	0.289
**Effect Size**	-	-	Small	Small	Small	Small
	**≤1**	0	4	8	16	29
	**>1**	65	61	57	49	36
**χ^2**	-	-	4.13	8.53	18.25	37.33
p	-	-	0	0	<0.001	<0.001
**ϕ**	-	-	0.178	0.256	0.375	0.536
**Effect Size**	-	-	Small	Small	Moderate	Large
	**≤1.5**	0	14	34	51	56
	**>1.5**	65	51	31	14	9
**χ^2**	-	-	15.69	46.042	83.924	98.378
p	-	-	<0.001	<0.001	<0.001	<0.001
**ϕ**	-	-	0.347	0.595	0.803	0.87
**Effect Size**	-	-	Moderate	Large	Large	Large
	**≤2**	2	33	52	59	60
	**>2**	63	32	13	6	5
**χ^2**	-	-	37.57	79.19	100.35	103.73
p	-	-	<0.001	<0.001	<0.001	<0.001
**ϕ**	-	-	0.538	0.78	0.879	0.893
**Effect Size**	-	-	Large	Large	Large	Large

RCSMS: Rhinoconjunctivitis Combined Symptom and Medication Score. χ^2: chi-square test; ϕ: Phi coefficient.
Effect sizes were as small (ϕ = 0.10), moderate (ϕ = 0.30) or large (ϕ ≥ 0.50). All comparisons were performed using Pearson's χ^2 test.

Table S30. Descriptive statistics of the RCSMS in the subset of patients who completed five years of AIT.

	RCSMS					
	**T0**	**T1**	**T2**	**T3**	**T4**	**T5**
**Mean**	4.15	2.47	1.54	1.13	1.00	1.17
**95% lower CI**	3.67	2.02	1.18	0.82	0.74	0.88
**95% upper CI**	4.63	2.93	1.91	1.44	1.26	1.45
**SD**	1.13	1.08	0.86	0.72	0.61	0.68
**Median**	4.67	2.42	1.58	1.33	1.33	1.33
**Q1**	3.54	1.54	1.33	0.33	0.21	1.04
**Q3**	5.00	3.13	1.67	1.67	1.46	1.50
**Maximum**	5.50	5.50	4.17	2.50	1.67	2.50
**Minimum**	1.67	1.17	0.00	0.00	0.00	0.00
**n**	24	24	24	23	24	24
**Reduction vs. T0$_{\text{Median}}$**	–	48.2%	66.1%	71.4%	71.4%	71.4%

CI: Confidence Interval. SD: Standard Deviation. Q1: First quartile. Q3: Third quartile. RCSMS: Rhinoconjunctivitis Combined Symptom and Medication Score.

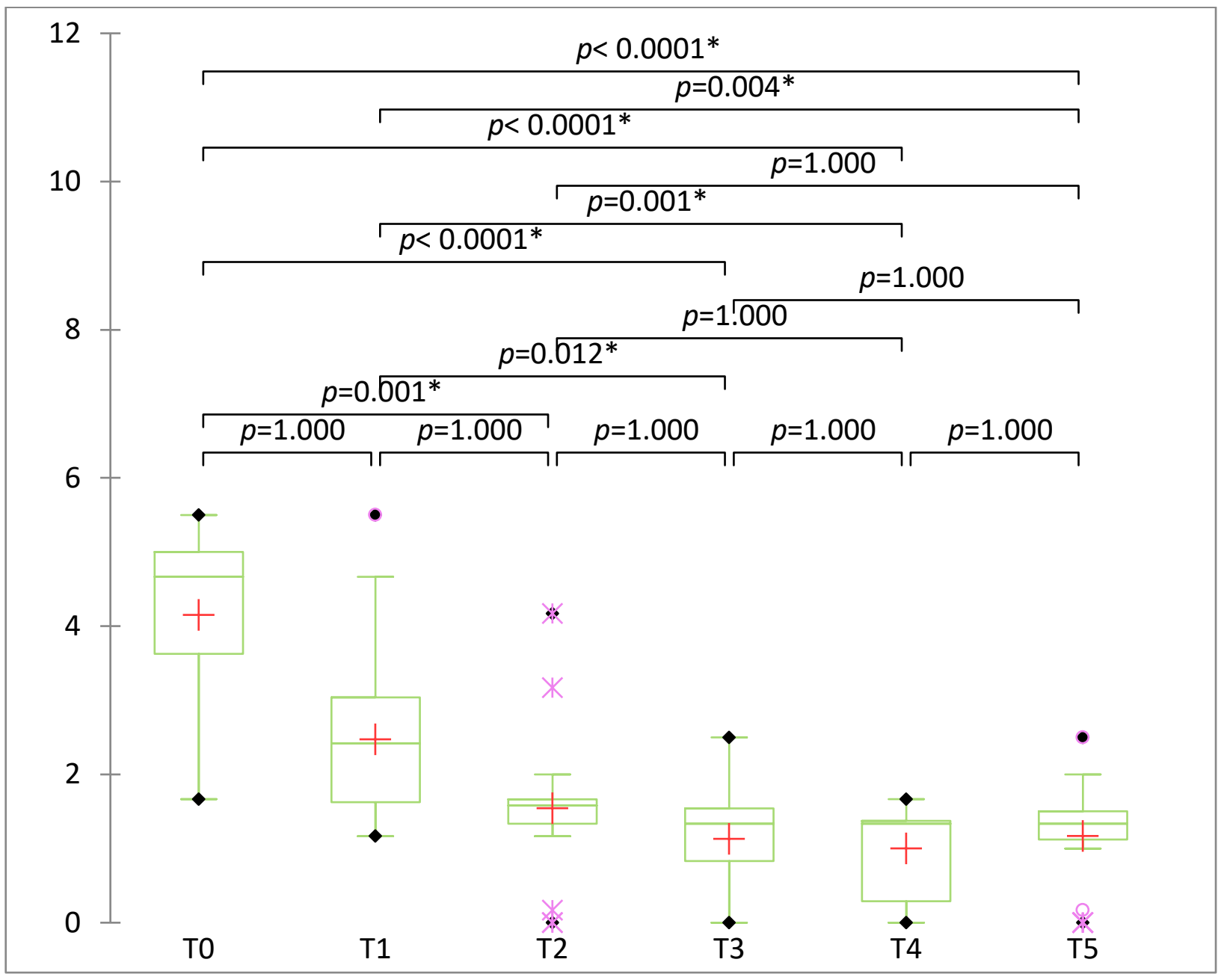

Figure S25. Evolution of the RCSMS over five years of AIT.
Boxes represent the interquartile range (IQR), horizontal lines indicate medians, and whiskers denote minimum and maximum values. Red crosses represent mean values. Pink symbols represent atypical values. *Reaching statistical significance at level α≤0.05. RCSMS: Rhinoconjunctivitis Combined Symptom and Medication Score.

Table S31. Distribution of patients across RCSMS levels and statistical comparison between baseline and follow-up at one, two, three, four and five years of AIT.

		RCSMS					
	**Score**	**T=0**	**T=1**	**T=2**	**T=3**	**T=4**	**T=5**
	**0**	0	0	2	5	3	4
	**>0**	24	24	22	19	21	20
**χ^2**	-	-	NA	2.087	5.581	3.2	4.364
p	-	-	NA	0.149	0.018	0.074	0.037
**ϕ**	-	-	NA	0.209	0.341	0.258	0.302
**Effect Size**	-	-	NA	Small	Moderate	Small	Moderate
	**≤1**	0	0	3	7	9	6
	**>1**	24	24	21	17	15	18
**χ^2**	-	-	NA	3.20	8.20	11.08	6.86
p	-	-	NA	0	0	<0.001	0
**ϕ**	-	-	NA	0.258	0.413	0.48	0.378
**Effect Size**	-	-	NA	Small	Moderate	Moderate	Moderate
	**≤1.5**	0	6	12	17	21	19
	**>1.5**	24	18	12	7	3	5
**χ^2**	-	-	6.857	16	26.323	37.333	31.448
p	-	-	0.009	<0.001	<0.001	<0.001	<0.001
**ϕ**	-	-	0.378	0.577	0.741	0.882	0.809
**Effect Size**	-	-	Moderate	Large	Large	Large	Large
	**≤2**	2	10	22	22	24	23
	**>2**	22	14	2	2	0	1
**χ^2**	-	-	7.11	33.33	33.33	40.62	36.81
p	-	-	0	<0.001	<0.001	<0.001	<0.001
**ϕ**	-	-	0.385	0.833	0.833	0.92	0.876
**Effect Size**	-	-	Moderate	Large	Large	Large	Large

RCSMS: Rhinoconjunctivitis Combined Symptom and Medication Score. χ^2: chi-square test; ϕ: Phi coefficient. NA: Not Applicable.
Effect sizes were as small (ϕ = 0.10), moderate (ϕ = 0.30) or large (ϕ ≥ 0.50). All comparisons were performed using Pearson's χ^2 test.

Table S32. Descriptive statistics of the ACSMS after one year of AIT.

	ACSMS	
	**T0**	**T1**
Mean	2.74	1.35
95% lower CI	2.52	1.15
95% upper CI	2.95	1.55
SD	1.22	1.14
Median	3.00	1.00
Q1	1.50	0.50
Q3	3.75	2.00
Maximum	4.50	4.50
Minimum	0.00	0.00
n	124	124
Reduction vs. $T0_{Median}$	–	66.7%

CI: Confidence Interval. SD: Standard Deviation. Q1: First quartile. Q3: Third quartile. ACSMS: Asthma Combined Symptom and Medication Score.

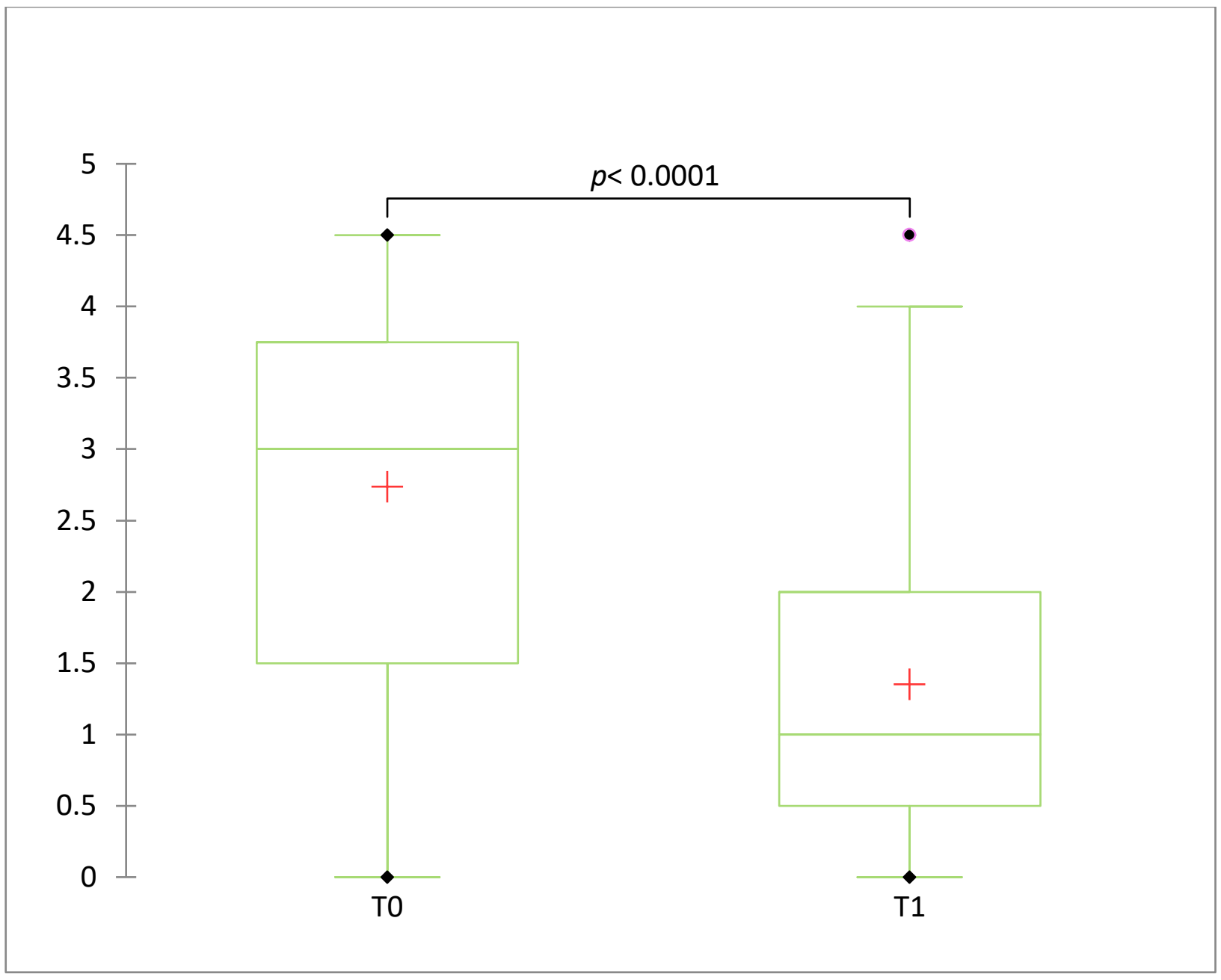

Figure S26. Evolution of the ACSMS during the first year of AIT.
Boxes represent the interquartile range (IQR), horizontal lines indicate medians, and whiskers denote minimum and maximum values. Red crosses represent mean values. Pink symbols represent atypical values. *Reaching statistical significance at level α≤0.05.

Table S33. Distribution of patients across ACSMS levels and statistical comparison between baseline and follow-up at one year of AIT.

		ACSMS	
	**Score**	**T=0**	**T=1**
	**0**	2	16
	**>0**	122	108
**χ^2**	-	-	11.741
p	-	-	<0.001
**ϕ**	-	-	0.218
**Effect Size**	-	-	Small
	**≤1**	12	70
	**>1**	112	54
**χ^2**	-	-	61.29
p	-	-	<0.001
**ϕ**	-	-	0.497
**Effect Size**	-	-	Moderate
	**≤1.5**	33	85
	**>1.5**	91	39
**χ^2**	-	-	43.715
p	-	-	<0.001
**ϕ**	-	-	0.42
**Effect Size**	-	-	Moderate
	**≤2**	44	97
	**>2**	80	27
**χ^2**	-	-	46.17
p	-	-	<0.001
**ϕ**	-	-	0.431
**Effect Size**	-	-	Moderate

ACSMS: Asthma Combined Symptom and Medication Score. χ^2: chi-square test; ϕ: Phi coefficient. Effect sizes were as small (ϕ = 0.10), moderate (ϕ = 0.30) or large (ϕ ≥ 0.50). All comparisons were performed using Pearson's χ^2 test.

Table S34. Descriptive statistics of the ACSMS in the subset of patients who completed two years of AIT.

	ACSMS		
	**T0**	**T1**	**T2**
**Mean**	2.81	1.41	0.84
**95% lower CI**	2.52	1.13	0.63
**95% upper CI**	3.10	1.69	1.04
**SD**	1.25	1.20	0.86
**Median**	3.00	1.00	0.75
**Q1**	1.63	0.50	0.13
**Q3**	4.00	2.25	1.00
**Maximum**	4.50	4.00	3.50
**Minimum**	0.00	0.00	0.00
**n**	73	73	73
**Reduction vs. $T0_{Median}$**	–	66.7%	75.0%

CI: Confidence Interval. SD: Standard Deviation. Q1: First quartile. Q3: Third quartile. ACSMS: Asthma Combined Symptom and Medication Score.

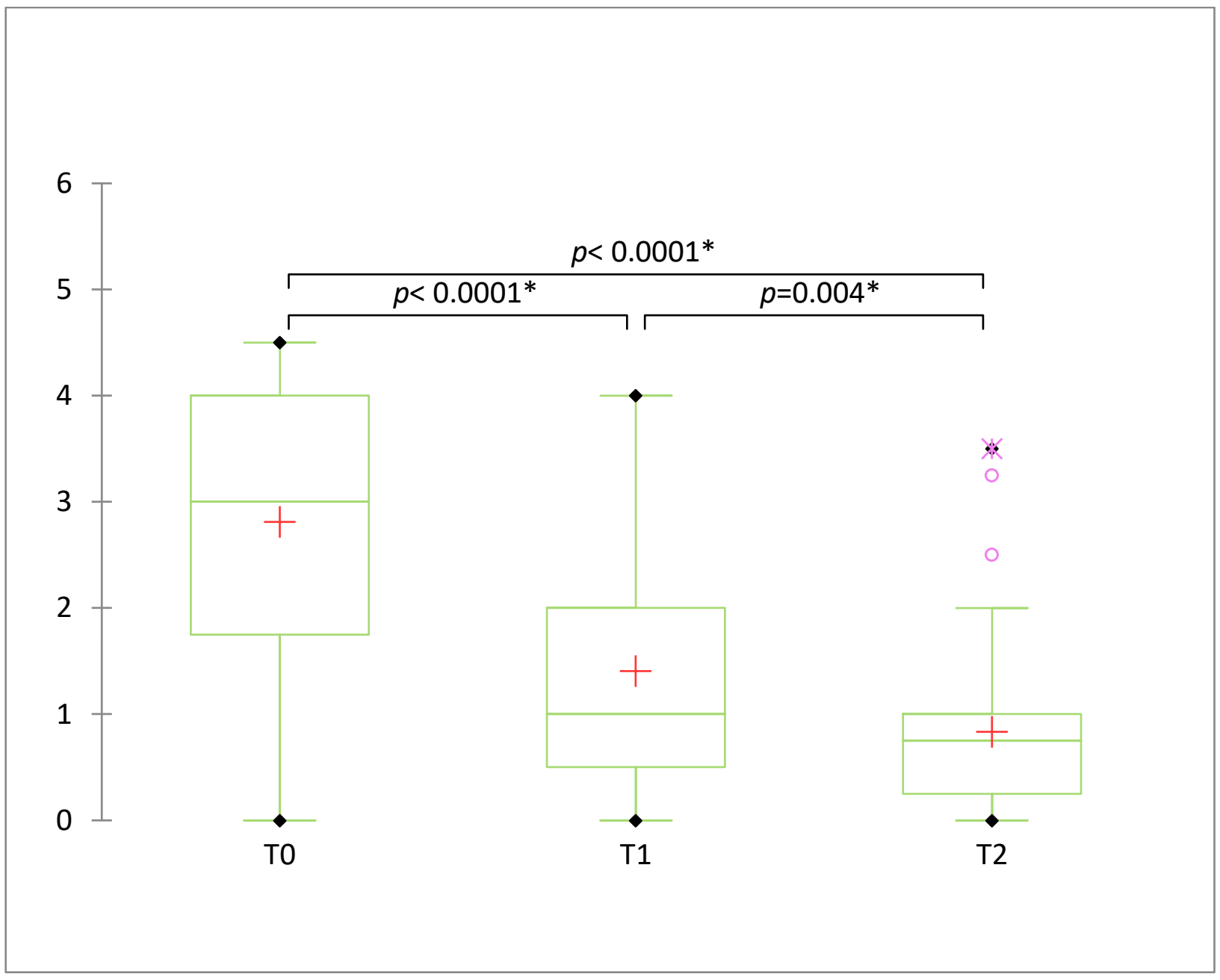

Figure S27. Evolution of the ACSMS over two years of AIT.
Boxes represent the interquartile range (IQR), horizontal lines indicate medians, and whiskers denote minimum and maximum values. Red crosses represent mean values. Pink symbols represent atypical values. *Reaching statistical significance at level α≤0.05.

Table S35. Distribution of patients across ACSMS levels and statistical comparison between baseline and follow-up at one and two years of AIT.

		ACSMS		
	**Score**	**T=0**	**T=1**	**T=2**
	**0**	2	10	18
	**>0**	71	63	55
**χ^2**	-	-	5.811	14.832
p	-	-	0.016	<0.001
**ϕ**	-	-	0.2	0.319
**Effect Size**	-	-	Small	Moderate
	**≤1**	8	41	58
	**>1**	65	32	15
**χ^2**	-	-	33.45	69.13
p	-	-	<0.001	<0.001
**ϕ**	-	-	0.479	0.688
**Effect Size**	-	-	Moderate	Large
	**≤1.5**	18	48	63
	**>1.5**	55	25	10
**χ^2**	-	-	24.886	56.154
p	-	-	<0.001	<0.001
**ϕ**	-	-	0.413	0.62
**Effect Size**	-	-	Moderate	Large
	**≤2**	24	55	67
	**>2**	49	18	6
**χ^2**	-	-	26.51	53.94
p	-	-	<0.001	<0.001
**ϕ**	-	-	0.426	0.608
**Effect Size**	-	-	Moderate	Large

ACSMS: Asthma Combined Symptom and Medication Score. χ^2: chi-square test; ϕ: Phi coefficient.
Effect sizes were as small (ϕ = 0.10), moderate (ϕ = 0.30) or large (ϕ ≥ 0.50). All comparisons were performed using Pearson's χ^2 test.

Table S36. Descriptive statistics of the ACSMS in the subset of patients who completed three years of AIT.

	ACSMS			
	**T0**	**T1**	**T2**	**T3**
**Mean**	2.79	1.37	0.83	0.64
**95% lower CI**	2.48	1.08	0.60	0.41
**95% upper CI**	3.10	1.66	1.05	0.88
**SD**	1.22	1.14	0.85	0.93
**Median**	3.00	1.00	0.50	0.50
**Q1**	1.50	0.50	0.06	0.00
**Q3**	3.88	2.00	1.00	0.75
**Maximum**	4.50	4.00	3.50	4.25
**Minimum**	0.00	0.00	0.00	0.00
**n**	61	61	60	61
**Reduction vs. $T0_{Median}$**	–	66.7%	83.3%	83.3%

CI: Confidence Interval. SD: Standard Deviation. Q1: First quartile. Q3: Third quartile. ACSMS: Asthma Combined Symptom and Medication Score.

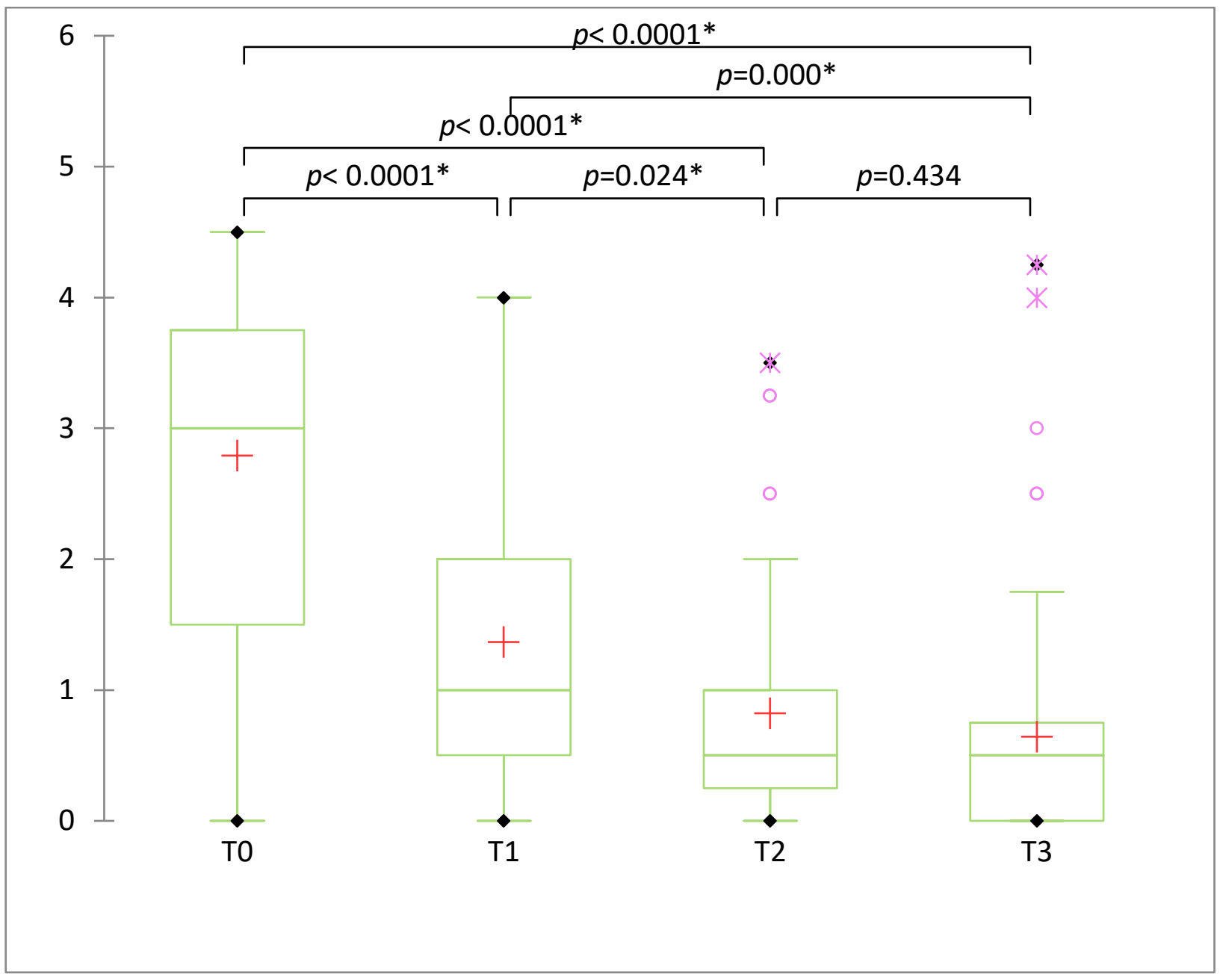

Figure S28. Evolution of the ACSMS over three years of AIT.
Boxes represent the interquartile range (IQR), horizontal lines indicate medians, and whiskers denote minimum and maximum values. Red crosses represent mean values. Pink symbols represent atypical values. *Reaching statistical significance at level α≤0.05.

Table S37. Distribution of patients across ACSMS levels and statistical comparison between baseline and follow-up at one, two and three years of AIT.

		ACSMS			
	**Score**	**T=0**	**T=1**	**T=2**	**T=3**
	**0**	1	6	15	26
	**>0**	60	55	46	35
**χ^2**	-	-	3.789	14.099	29.727
p	-	-	0.052	<0.001	<0.001
**ϕ**	-	-	0.176	0.34	0.494
**Effect Size**	-	-	Small	Moderate	Moderate
	**≤1**	6	35	47	52
	**>1**	55	26	14	9
**χ^2**	-	-	30.90	56.08	69.55
p	-	-	<0.001	<0.001	<0.001
**ϕ**	-	-	0.503	0.678	0.755
**Effect Size**	-	-	Large	Large	Large
	**≤1.5**	16	41	52	55
	**>1.5**	45	20	9	6
**χ^2**	-	-	20.58	43.059	51.246
p	-	-	<0.001	<0.001	<0.001
**ϕ**	-	-	0.411	0.594	0.648
**Effect Size**	-	-	Moderate	Large	Large
	**≤2**	20	48	55	56
	**>2**	41	13	6	5
**χ^2**	-	-	26.05	42.40	45.23
p	-	-	<0.001	<0.001	<0.001
**ϕ**	-	-	0.462	0.59	0.609
**Effect Size**	-	-	Moderate	Large	Large

ACSMS: Asthma Combined Symptom and Medication Score. χ^2: chi-square test; ϕ: Phi coefficient.
Effect sizes were as small (ϕ = 0.10), moderate (ϕ = 0.30) or large (ϕ ≥ 0.50). All comparisons were performed using Pearson's χ^2 test.

Table S38. Descriptive statistics of the ACSMS in the subset of patients who completed four years of AIT.

	ACSMS				
	T0	T1	T2	T3	T4
**Mean**	2.81	1.15	0.78	0.61	0.46
**95% lower CI**	2.43	0.82	0.54	0.32	0.22
**95% upper CI**	3.18	1.48	1.03	0.91	0.69
**SD**	1.11	0.98	0.74	0.87	0.69
**Median**	2.88	0.75	0.75	0.50	0.00
**Q1**	1.75	0.50	0.00	0.00	0.00
**Q3**	3.69	1.88	1.00	0.94	1.00
**Maximum**	4.50	3.75	3.25	4.25	2.25
**Minimum**	1.00	0.00	0.00	0.00	0.00
**n**	36	36	36	36	36
**Reduction vs. $T0_{Median}$**	–	73.9%	73.9%	82.6%	100.0%

CI: Confidence Interval. SD: Standard Deviation. Q1: First quartile. Q3: Third quartile. ACSMS: Asthma Combined Symptom and Medication Score.

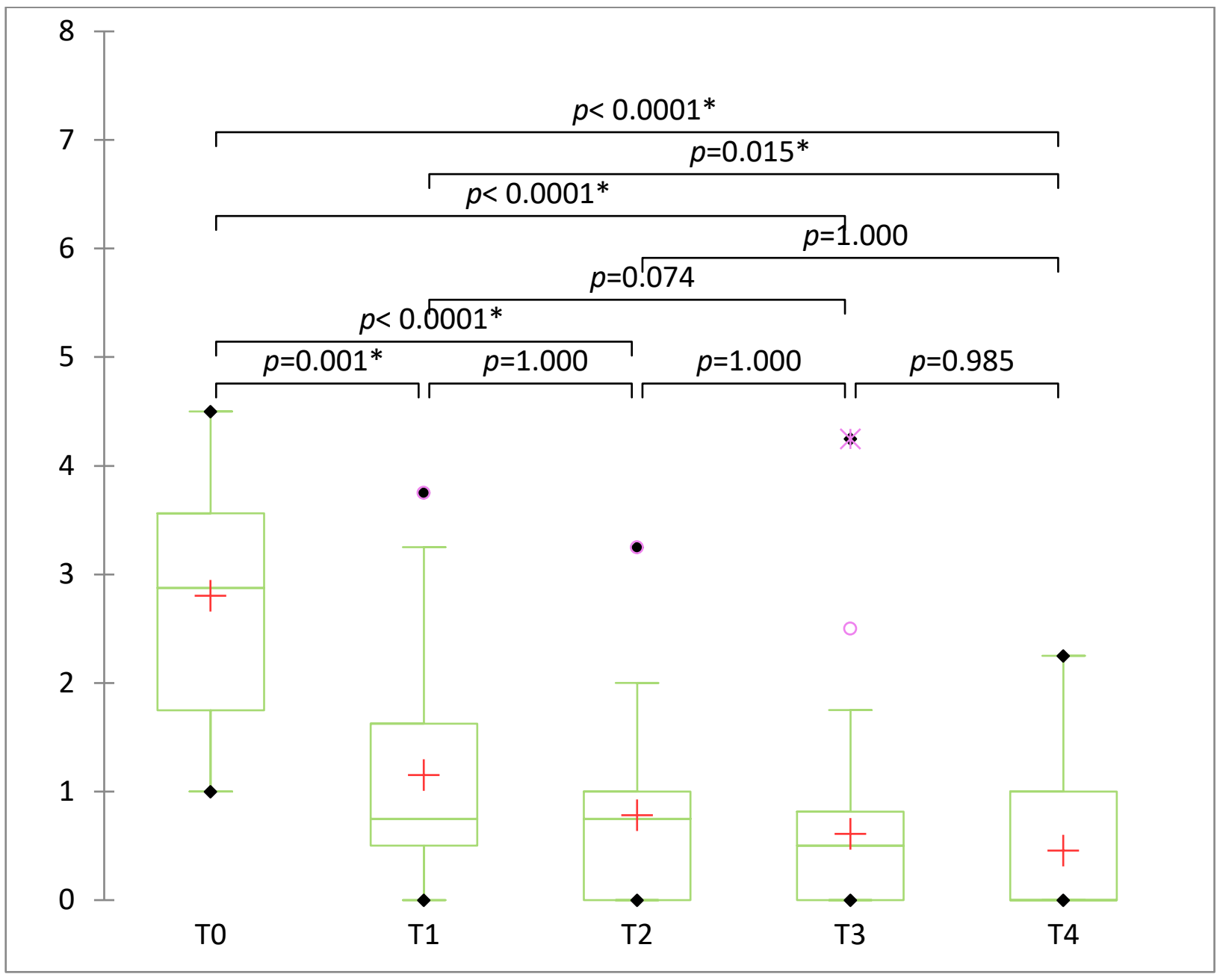

Figure S29. Evolution of the ACSMS over four years of AIT.
Boxes represent the interquartile range (IQR), horizontal lines indicate medians, and whiskers denote minimum and maximum values. Red crosses represent mean values. Pink symbols represent atypical values. *Reaching statistical significance at level α≤0.05.

Table S39. Distribution of patients across ACSMS levels and statistical comparison between baseline and follow-up at one, two, three and four years of AIT.

		ACSMS				
	**Score**	**T=0**	**T=1**	**T=2**	**T=3**	**T=4**
	**0**	0	4	10	16	23
	**>0**	36	32	26	20	13
**χ^2**	-	-	4.235	11.613	20.571	33.796
p	-	-	0.04	<0.001	<0.001	<0.001
**ϕ**	-	-	0.243	0.402	0.535	0.685
**Effect Size**	-	-	Small	Moderate	Large	Large
	**≤1**	3	23	28	31	30
	**>1**	33	13	8	5	6
**χ^2**	-	-	24.08	35.41	43.69	40.78
p	-	-	<0.001	<0.001	<0.001	<0.001
**ϕ**	-	-	0.578	0.701	0.779	0.753
**Effect Size**	-	-	Large	Large	Large	Large
	**≤1.5**	8	27	32	33	34
	**>1.5**	28	9	4	3	2
**χ^2**	-	-	20.071	32.4	35.405	38.629
p	-	-	<0.001	<0.001	<0.001	<0.001
**ϕ**	-	-	0.528	0.671	0.701	0.732
**Effect Size**	-	-	Large	Large	Large	Large
	**≤2**	11	31	35	34	34
	**>2**	25	5	1	2	2
**χ^2**	-	-	22.86	34.68	31.35	31.35
p	-	-	<0.001	<0.001	<0.001	<0.001
**ϕ**	-	-	0.563	0.694	0.66	0.66
**Effect Size**	-	-	Large	Large	Large	Large

ACSMS: Asthma Combined Symptom and Medication Score. χ^2: chi-square test; ϕ: Phi coefficient. Effect sizes were as small (ϕ = 0.10), moderate (ϕ = 0.30) or large ($\phi \geq$ 0.50). All comparisons were performed using Pearson's χ^2 test.

Table S40. Descriptive statistics of the ACSMS in the subset of patients who completed five years of AIT.

	ACSMS					
	T0	T1	T2	T3	T4	T5
**Mean**	2.71	1.38	0.99	0.94	0.76	0.66
**95% lower CI**	2.09	0.81	0.55	0.36	0.34	0.21
**95% upper CI**	3.32	1.96	1.42	1.52	1.19	1.12
**SD**	1.20	1.12	0.85	1.13	0.83	0.88
**Median**	2.75	1.00	0.75	0.75	0.75	0.00
**Q1**	1.63	0.63	0.50	0.00	0.00	0.00
**Q3**	3.88	2.25	1.50	1.50	1.50	1.50
**Maximum**	4.50	3.75	3.25	4.25	2.25	2.50
**Minimum**	1.00	0.00	0.00	0.00	0.00	0.00
**n**	17	17	17	17	17	17
**Reduction vs. $T0_{Median}$**	–	63.6%	72.7%	72.7%	72.7%	100.0%

CI: Confidence Interval. SD: Standard Deviation. Q1: First quartile. Q3: Third quartile. ACSMS: Asthma Combined Symptom and Medication Score.

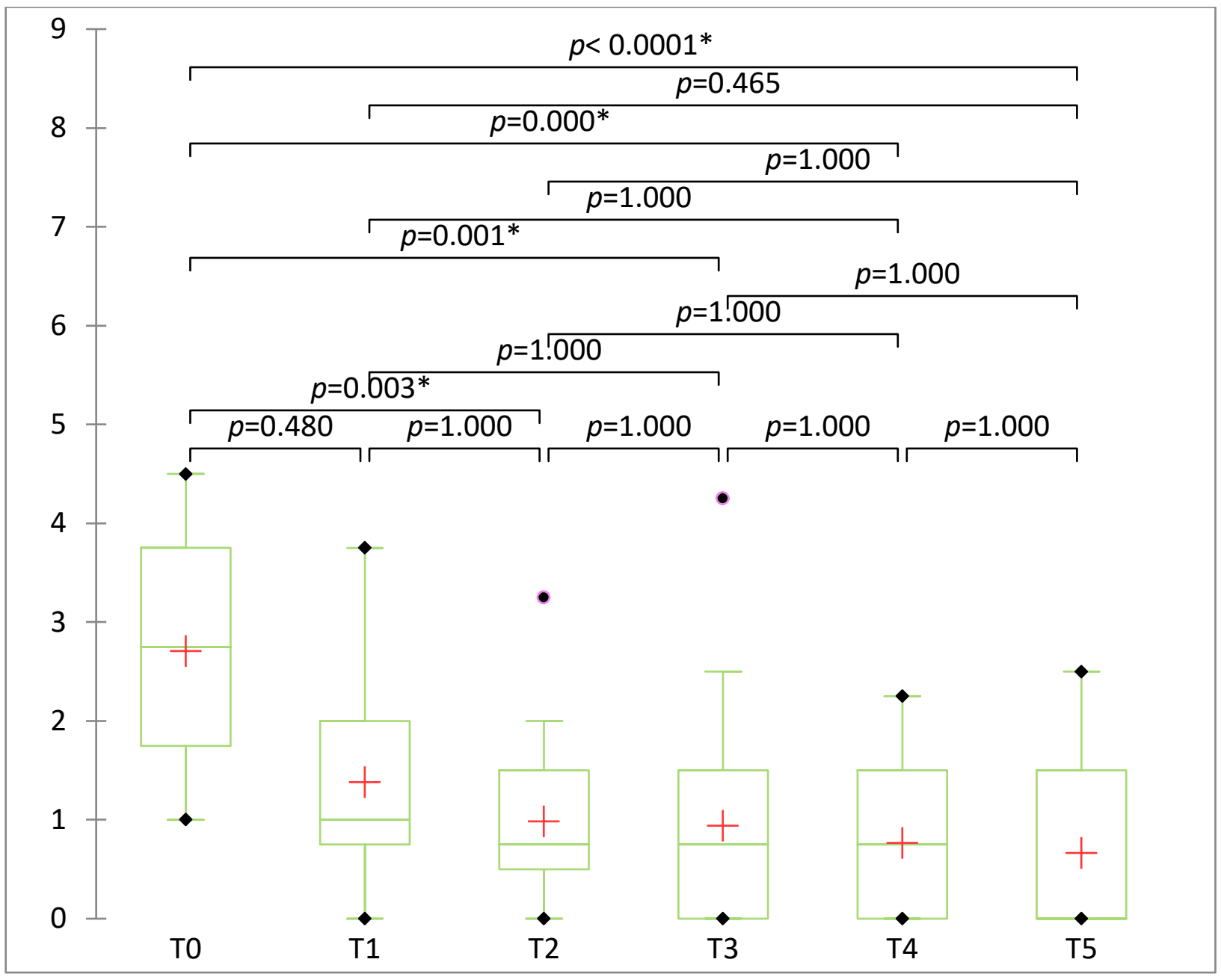

Figure S30. Evolution of the ACSMS over five years of AIT.
Boxes represent the interquartile range (IQR), horizontal lines indicate medians, and whiskers denote minimum and maximum values. Red crosses represent mean values. Pink symbols represent atypical values. *Reaching statistical significance at level α≤0.05.

Table S41. Distribution of patients across ACSMS levels and statistical comparison between baseline and follow-up at one, two, three, four and five years of AIT.

		ACSMS					
	**Score**	**T=0**	**T=1**	**T=2**	**T=3**	**T=4**	**T=5**
	**0**	0	1	3	6	8	9
	**>0**	17	16	14	11	9	8
**χ^2**	-	-	1.03	3.29	7.286	10.462	12.24
p	-	-	0.31	0.07	0.007	0.001	<0.001
**ϕ**	-	-	0.174	0.311	0.463	0.555	0.6
**Effect Size**	-	-	Small	Moderate	Moderate	Large	Large
	**≤1**	1	10	11	12	11	12
	**>1**	16	7	6	5	6	5
**χ^2**	-	-	10.89	12.88	15.07	12.88	15.07
p	-	-	<0.001	<0.001	<0.001	<0.001	<0.001
**ϕ**	-	-	0.566	0.615	0.666	0.615	0.666
**Effect Size**	-	-	Large	Large	Large	Large	Large
	**≤1.5**	4	12	14	14	15	14
	**>1.5**	13	5	3	3	2	3
**χ^2**	-	-	7.556	11.806	11.806	14.435	11.806
p	-	-	0.006	<0.001	<0.001	<0.001	<0.001
**ϕ**	-	-	0.471	0.589	0.589	0.652	0.589
**Effect Size**	-	-	Moderate	Large	Large	Large	Large
	**≤2**	7	13	16	15	15	16
	**>2**	10	4	1	2	2	1
**χ^2**	-	-	4.37	10.89	8.24	8.24	10.89
p	-	-	0	<0.001	0	0	<0.001
**ϕ**	-	-	0.359	0.566	0.492	0.492	0.566
**Effect Size**	-	-	Moderate	Large	Moderate	Moderate	Large

ACSMS: Asthma Combined Symptom and Medication Score. χ^2: chi-square test; ϕ: Phi coefficient. Effect sizes were as small (ϕ = 0.10), moderate (ϕ = 0.30) or large (ϕ ≥ 0.50). All comparisons were performed using Pearson's χ^2 test.
